# Specific enrichment of somatic single nucleotide variants in Alzheimer-associated tetraploid neurons

**DOI:** 10.3389/fnmol.2026.1929505

**Published:** 2026-10-21

**Authors:** Noelia López-Sánchez, Alberto Rábano, José María Frade

**Affiliations:** 1 Department of Molecular and Cellular Neuroscience, CSIC, Centro de Neurociencias Cajal, Alcalá de Henares, Spain; 2 Tetraneuron, Madrid, Spain; 3 Department of Neuropathology and Tissue Bank, Fundacion CIEN, Instituto de Salud Carlos III, Madrid, Spain

**Keywords:** Alzheimer-associated genes, cancer-related genes, DNA repair genes, molecular pathogenicity score, oxidative stress response genes, Parkinson-associated genes, senescence-associated genes, two-hit hypothesis

## Abstract

An early pathological process observed in the brains of patients with Alzheimer’s disease (AD) is the reactivation of the cell cycle in neurons, followed by somatic neuronal tetraploidization (NT). NT increases with age, and aging has been shown to correlate with the accumulation of DNA somatic mutations in neurons. In this study, we evaluated the presence of somatic mutations, including single nucleotide variants (SNVs) and short insertions or deletions (INDELs), in genomic DNA from tetraploid neurons obtained from the parietal cortex of patients with AD, as compared with diploid neurons from control individuals and patients with AD. Here we show that, in contrast to somatic INDELs, somatic SNVs (sSNVs) are significantly increased in the exome of tetraploid neurons, with elevated levels of T to C (A to G) transitions, a type of mutation associated with oxidative stress. This finding correlates with the overrepresentation of sSNVs in genes involved in the oxidative stress response and DNA repair, with DNA repair genes showing significantly enhanced molecular pathogenicity scores (MPS). Moreover, sSNVs affecting cancer- and senescence-related genes show a greater MPS than those from a random sample of genes. We propose a model in which neuronal tetraploidy may be stochastically triggered in neurons with mutated DNA repair genes under oxidative stress, potentially exacerbating oxidative DNA damage. This could facilitate cell cycle progression, followed by tetraploidization and senescence in the affected neurons, a process that may contribute to the pathogenesis of AD.

## 1 Introduction

Alzheimer’s disease (AD) is the most prominent cause of dementia and a major health threat, as it affects a substantial proportion of the elderly in Western countries. One of the earliest pathological events observed in AD is the re-entry of neurons into the cell cycle (Frade and Ovejero-Benito, 2015), which may be followed by DNA synthesis. This results in neuronal hyperploidy (including full DNA replication generating somatic tetraploid neurons with four times the haploid DNA content: 4C) (Yang et al., 2001; Mosch et al., 2007; Arendt et al., 2010; López-Sánchez et al., 2017) and the specific death of hyperploid neurons at later stages (Arendt et al., 2010; Barrio-Alonso et al., 2018). Compelling evidence indicates that neuronal tetraploidy (NT) precedes and recapitulates AD (López-Sánchez et al., 2017), with more than 2% of neurons exhibiting a tetraploid phenotype in both the parietal and prefrontal cortices of Braak V/VI patients, compared with the remaining diploid neuronal population. This proportion was markedly increased in specific neuronal populations, with more than 6% of MEF2C + neurons and approximately 4% of Etv1 + neurons exhibiting a tetraploid phenotype in these cortical regions of Braak V/VI patients. Importantly, NT prevention in the cerebral cortex of 5xFAD mice, a murine model of AD (Oakley et al., 2006), is correlated with enhanced cognition (López-Sánchez et al., 2021; López-Sánchez et al., 2022). NT has been associated with alterations in neuronal structure and function (Frade and López-Sánchez, 2010) and correlates with synaptic dysfunction (Barrio-Alonso et al., 2018), which may disturb the proper functioning of neural networks and potentially contribute to cognitive deficits (Barrio-Alonso et al., 2020). Furthermore, forced cell cycle re-entry in neurons results in the classical hallmarks of AD, including tau protein hyperphosphorylation and neurofibrillary tangles (NFT), increased APP processing and extracellular deposits of Aβ, delayed neuronal cell death, gliosis, and cognitive deficits (Lee et al., 2009; McShea et al., 2007; Park and Barrett, 2020; Park et al., 2007). Musi et al. (2018) have shown that genes involved in cell cycle progression are upregulated in human NFT-containing neurons, likely being senescent cells (Wong and Chow, 2023; Wu et al., 2024). Moreover, a detailed proteomic analysis performed in six different brain regions from AD patients, compared with control individuals, identified several signaling pathways involved in cell cycle regulation as being widely dysregulated in severely affected regions of the AD brain (Xu et al., 2019). Overall, this evidence indicates that NT participates in the etiology of AD.

The mechanism leading to neuronal cell cycle re-entry and somatic neuronal tetraploidization (SNT) in AD is currently unknown. Since aging is the main risk factor for sporadic AD (Guerreiro and Bras, 2015) and is associated with the accumulation of somatic mutations in neurons (Lodato et al., 2018), we hypothesized that AD-associated tetraploid neurons would be enriched in age-related somatic mutations. In addition to large genomic structural variants, two types of small somatic mutations accumulate during aging: single nucleotide variants (SNVs) and short insertions or deletions (INDELs) (Vijg, 2021). We therefore sought to determine whether the SNVs and INDELs that accumulate in the aging human brain (Lodato et al., 2018) are enriched in AD-associated tetraploid neurons, which progressively accumulate before the appearance of the neuropathological hallmarks of sporadic AD (López-Sánchez et al., 2017).

To test the hypothesis that somatic neuronal tetraploidization in AD is associated with specific patterns of somatic DNA mutations, we performed whole-exome sequencing of tetraploid (4C) and diploid (2C) neurons isolated from the parietal cortex of patients with AD at Braak stages V/VI (Braak and Braak, 1991), when NT reaches its highest levels in this brain region (López-Sánchez et al., 2017). As a control, we used the cell nuclei of diploid neurons isolated from the parietal cortex of control individuals. Since SNT is absent in the parietal cortex of control individuals (López-Sánchez et al., 2017), we decided not to isolate tetraploid neurons from this tissue in control individuals. This analysis indicated that an increase in oxidative stress-associated somatic SNVs (sSNVs), mostly T > C transitions, can be found in tetraploid neurons from AD patients compared to diploid (AD) or control (control) neurons. This effect is associated with the enhanced accumulation of sSNVs in genes regulating the oxidative stress response and DNA repair. The accumulation of sSNVs in AD-risk genes could also be observed in tetraploid neurons, but its involvement in the etiology of AD remains unclear, as an increase in sSNVs in risk genes for Parkinson’s disease was also evident. The high molecular pathogenicity score (MPS) of the sSNVs observed in genes involved in both cancer and senescence suggests that somatic neuronal tetraploidization can be assimilated to an oncogenic-like process, as previously suggested by Zhu et al. (2004, 2007), which may totally or partially generate the senescent neurons that have been observed in the AD brain (Dehkordi et al., 2021; Jurk et al., 2012; Wong et al., 2023; Wu et al., 2024).

## 2 Materials and methods

### 2.1 Human samples

Samples of parietal cortex from patients with Alzheimer’s disease (*n* = 3) and healthy controls (*n* = 3) were provided by the Banco de Tejidos Fundación CIEN (Madrid, Spain) and Banco de Cerebros de la Región de Murcia (Hospital Virgen de la Arrixaca, Murcia, Spain). Procedures have been approved by the Scientific Committee of BT-CIEN and the Ethics Committee of Consejo Superior de Investigaciones Científicas (CSIC). Written informed consent for brain removal after death for diagnostic and research purposes was obtained from brain donors and/or next of kin. Immediately after post-mortem brain extraction, each brain was sagittally cut, and slices from the right brain half were frozen in −60 °C isopentane. A full neuropathological examination of each brain was performed on the left half, and samples from either Braak 0 (control) or Braak V–VI patients were chosen for this analysis (Supplementary Table S1). The severity of Alzheimer’s pathology was scored according to the National Institute on Aging–Alzheimer’s Association Guidelines, following the “ABC” protocol (Montine et al., 2012). Consequently, the total amyloid burden (“A” score) was determined according to the Thal staging system; the stage of neurofibrillary pathology was established according to the Braak (“B” score) scheme; and the frequency of neuritic plaques in associative cortex according to the CERAD protocol (“C” score) was registered. The identification codes of patients with AD and control individuals were not known to anyone outside the research group.

### 2.2 Antibodies

Rabbit anti-NeuN polyclonal antibody (Merck Millipore) and mouse anti-NeuN monoclonal antibody (clone A60, Merck Millipore) were both diluted 1/800 for flow cytometry. Secondary anti-mouse or anti-rabbit antibodies, generated in donkey or goat and conjugated to Alexa Fluor 488, were purchased from Invitrogen. These antibodies were used at 1/400 dilution for cytometry.

### 2.3 Cell nuclei isolation and immunostaining

Cell nuclei isolation was performed as previously described (López-Sánchez et al., 2017). Briefly, a cube of 5–8 mm edge of fresh-frozen human parietal cortex was placed in 3.0 mL ice-cold, DNase-free PBS containing 0.1% Triton X-100 (Sigma-Aldrich) (PTx) containing protease inhibitor cocktail (Roche). Cell nuclei were then isolated by mechanical disaggregation using a Dounce homogenizer and diluted to 4.5 mL with PTx. Undissociated tissue was removed by centrifugation in 1.5-mL minifuge tubes at 200 × g for 1.5 min at 4 °C. Supernatants were reserved, and pellets were washed with 1.5 mL PTx and centrifuged at 100 × g for 2 min at 4 °C. The supernatants were collected and added to the previous ones. These samples were 8-fold diluted with PTx and centrifuged at 400 × g for 4 min at 4 °C. Supernatants, which lacked cell nuclei, were discarded, and the pellet was incubated at 4 °C in 800–1,000 μl cold PTx for at least 10–20 min, prior to mechanical disaggregation by gently swirling the vial. The quality and purity of the isolated nuclei were examined microscopically after staining with 100 ng/mL DAPI.

Nuclear immunostaining with anti-NeuN antibodies was performed as described previously (López-Sánchez et al., 2017). Briefly, primary and secondary antibodies were added to the isolated unfixed nuclei containing 5% fetal calf serum (FCS) and 1.25 mg/mL BSA. In the control samples, primary antibodies were excluded. Finally, the reaction was incubated O/N at 4 °C in the dark. Immunostained nuclei were filtered through a 40-μm nylon filter, which retained large aggregates but not nuclei. The volume was adjusted to 800–1,000 μl with DNase-free PTx containing 40 μg/mL PI (Sigma) and 25 μg/mL DNase-free RNase I (Sigma) and incubated for 30 min at RT. The quality of the nuclei and the specificity of the immunostaining signal were checked using fluorescence microscopy.

### 2.4 FACS

Flow cytometry was performed as described previously (López-Sánchez et al., 2017), using a FACSAria cytometer (BD Biosciences, San Diego, CA) equipped with a 488-nm Coherent Sapphire solid-state and 633-nm JDS Uniphase HeNe air-cooled laser. The emission filters used were BP 530/30 for Alexa 488 and BP 616/23 for PI. Data were analyzed using FACSDiva (BD Biosciences) and Weasel 3.0.1 (Walter and Eliza Hall Institute of Medical Research) software and displayed using biexponential scaling. Cellular debris, which was clearly differentiated from the nuclei owing to its inability to incorporate PI, was gated and excluded from the analysis. DNA content histograms were generated by excluding doublets and clumps by gating on the DNA pulse area versus its corresponding pulse height. For maximum doublet resolution, a minimal flow rate was used in all experiments. The exclusion of doublets was confirmed by examining the DNA pulse area versus the pulse width of the selected population. Two rounds of cell sorting were performed to obtain a population of NeuN-positive nuclei enriched in tetraploid neurons (4C samples). Then, an aliquot of each 4C sample was used to estimate the proportion of tetraploid nuclei through flow cytometry, quantified by using the BD FACSDiva Software. The final proportion of tetraploid neurons in the 4C samples was above 70%, except for individual AD166, which was discarded from the analysis.

### 2.5 Whole genome amplification (WGA)

Genomic DNA from 430-550 diploid or tetraploid neuronal nuclei was isolated and amplified using the PicoPLEX WGA kit (New England Biolabs). We acknowledge that WGA can introduce amplification biases and allelic dropout, which may affect the detection of low-frequency variants. Future studies should validate these key findings using orthogonal sequencing methods without amplification. WGA amplification included one cycle at 95 °C (2 min), followed by 16 cycles at 95 °C (15 s), 15 °C (50 s), 25 °C (40 s), 35 °C (30 s), 65 °C (40 s), and 75 °C (40 s). Successful amplification was confirmed using agarose gel electrophoresis. DNA from PicoPLEX was purified using the Gene Elute PCR clean-up kit (Sigma-Aldrich). Cleaned-up products were quantified using a NanoDrop spectrophotometer (Thermo Fisher Scientific).

### 2.6 Libraries preparation, exome sequencing, and bioinformatics analysis

Whole exome libraries were prepared using SureSelectXT Human All Exon V6 (Agilent Technologies, Santa Clara, CA, United States) and sequenced on an Illumina system platform (Illumina, San Diego, CA, United States). The raw FASTQ files generated were first evaluated using FastQC^1^ and Trimmomatic (Bolger et al., 2014) to remove bases, adapters, and other low-quality sequences from the data. Each FASTQ file was then aligned against the GRCh38/hg38 version of the reference human genome using BWA-mem (Li and Durbin, 2010). BAM files were generated using SAMtools (Li et al., 2009), and optical and PCR duplicates were eliminated using Sambamba (Tarasov et al., 2015). SNVs and INDELs calling was performed using the VarScan 2 (Koboldt et al., 2012) and LoFreq (Wilm et al., 2012) algorithms. Variants were annotated with information from the following databases: functional information (Ensembl, Pfam), population information (dbSNP, 1000 Genomes, ESP6500, ExAC, gnomAD), in silico prediction of functional impact (dbNSFP, dbscSNV), cancer-related information (COSMIC, ICGC), and clinical and phenotypic trait information (ClinVar, OMIM, HPO). The tetraploid neuron-specific variants in each AD patient were identified as those with null allele frequency (AF) in the diploid sample and AF > 0 in the tetraploid sample. All statistical analyses were performed using R software v4.1.2. In cases where conditions were compared, the Student’s t-test was used with *p*-value < 0.05 as the significance threshold. SNVs and INDELs were analyzed separately. Both the percentage of variants per chromosome and the number of variants per chromosome size were calculated among the samples and conditions. The density and frequency of these changes are represented across each chromosome. In the case of SNVs variants, the number and frequency of each type of change were also analyzed. Likewise, the same analysis was performed by pre-selecting those variants affecting genes related to Alzheimer’s disease and Parkinson’s disease (based on literature search), cancer (based on 409 proto-oncogenes and tumor suppressor genes that are used for the diagnosis of human cancer [OncoNIM Seq409 diagnostic system (NIM Genetics)]), senescence (Dehkordi et al., 2021), and DNA repair and response to oxidative stress (based on gene ontology search) (see Supplementary Table S2 for a list of the respective genes that were analyzed per group). The molecular pathogenicity of proteins was evaluated using the DG prediction score (Dreamgenics^2^), calculated from the dbNSFP database (Liu et al., 2011, 2013, 2016) that combines the in silico predictions of 12 different algorithms: ALoFT (Balasubramanian et al., 2017), DEOGEN2 (Raimondi et al., 2017), FATHMM (Shihab et al., 2013), FATHMM-MKL,^3^ FATHMM-XF (see text footnote 3), META-LR (Dong et al., 2015), META-SVM (Dong et al., 2015), SIFT (Johnson et al., 2005), PROVEAN (Choi et al., 2012), Mutation Assessor (Frousios et al., 2013), Mutation Taster (Zhang et al., 2012), and LRT (Chun and Fay, 2009). LoFreq calls showing AF minor than 0.05 were selected and filtered for quality (QUAL) > 30 on the Galaxy platform (Galaxy Community, 2022).

To accommodate small sample sizes, a non-parametric bootstrap sensitivity analysis was performed using an estimation-statistics framework focused on effect magnitude and precision. This bootstrap resampling analysis were performed in Python (v3.13) using pandas and NumPy. Bootstrap distributions were generated using 5,000 resampling iterations with replacement (random seed = 42). Two-sided 95% percentile confidence intervals (CIs) were calculated for group means and pairwise differences (AD 2C vs. NoAD, AD 4C vs. NoAD, and AD 4C vs. AD 2C). Differences were considered compatible with a non-zero effect when their 95% CIs did not include zero. These bootstrap estimates served strictly to quantify uncertainty and evaluate result stability, rather than to increase the effective sample size or statistical power. The bootstrap analyses yielded results consistent with those obtained using the original parametric statistical analyses, supporting the robustness of our findings (see Supplementary Tables S3–S42). The full concordance with the traditional parametric results reinforces the reliability and stability of the observed group differences (Kelley, 2005).

Mutational signatures were analyzed using the Signal platform^5^ (Degasperi et al., 2020), which provides an expanded and updated reference database of mutational signatures derived from large-scale cancer sequencing datasets and supports sample-level signature assignment. SNVs identified using the LoFreq variant-calling approach were fitted to the consensus reference single-base substitution (SBS) signatures described by Degasperi et al. (2020). This expanded repertoire of SBS signatures was derived from whole-genome sequencing of 12,222 tumor–normal pairs from the UK 100,000 Genomes Project and validated in independent cohorts (Degasperi et al., 2022). Signature fitting was performed using the FitMS method with the default SBS panel, allowing a maximum of two rare signatures (Max 2). One hundred bootstrap iterations were performed using fixed sparsity, with a 5% sparsity threshold and a *p*-value threshold of 0.05. Signature contributions were expressed as the percentage of fitted mutations, and the similarity between the observed and reconstructed mutational profiles was assessed using cosine similarity.

### 2.7 Use of artificial intelligence (AI)

AI-assisted language editing (Paperpal^6^) was used to improve the grammar, clarity, and readability of the manuscript.

Python scripts for the bootstrap resampling analysis incorporated syntax assistance from AI language models (ChatGPT by OpenAI and Gemini by Google), with all code being manually reviewed, tested, and executed by the authors.

No AI tools were used to generate, analyze, or interpret scientific data, draw conclusions, or make scientific decisions. All manuscript content was critically reviewed, verified, and approved by the authors, who take full responsibility for its accuracy and integrity.

## 3 Results

### 3.1 Increase of SNVs, but not INDELs, in tetraploid neurons from AD patients

To investigate whether AD-associated tetraploid neurons exhibit an increased burden of age-related somatic mutations, we quantified the absolute number of mutations, including SNVs and INDELs, in genomic DNA from tetraploid neurons obtained from the parietal cortex of AD patients compared with diploid neurons from control individuals (NoAD) and AD patients. To this aim, whole genomic DNA from 430–550 neuronal (NeuN+) nuclei per individual were whole-genome amplified (Supplementary Table S1) and subjected to deep next-generation sequencing, as described in the methodological section. This analysis indicated that AD-derived tetraploid neurons contained a statistically significant increase (16%) in mutations compared to diploid neurons from patients with AD (Figure 1A). In contrast, no significant differences were observed between diploid neurons from AD patients and control (NoAD) individuals, consistent with the absence of an apparent age-related bias in our analysis, as previously reported by Li et al. (2023). These mutations in AD-derived tetraploid neurons affected chromosomal regions similar to those detected in diploid neurons from NoAD and AD patients (see circos plots in Figure 1B), consistent with the presence of specific mutation hot spots in the genome of human neurons, as in other cell types, likely due to aberrant repair of breaks in sequence-specific contexts (Nesta et al., 2021). The observed increase in mutations in tetraploid neurons was specific for SNVs, representing a 17% increase compared with diploid neurons from patients with AD (Figure 1C), since the absolute number of INDELs was similar in all neuronal types (Figure 1C).

**Figure 1:**
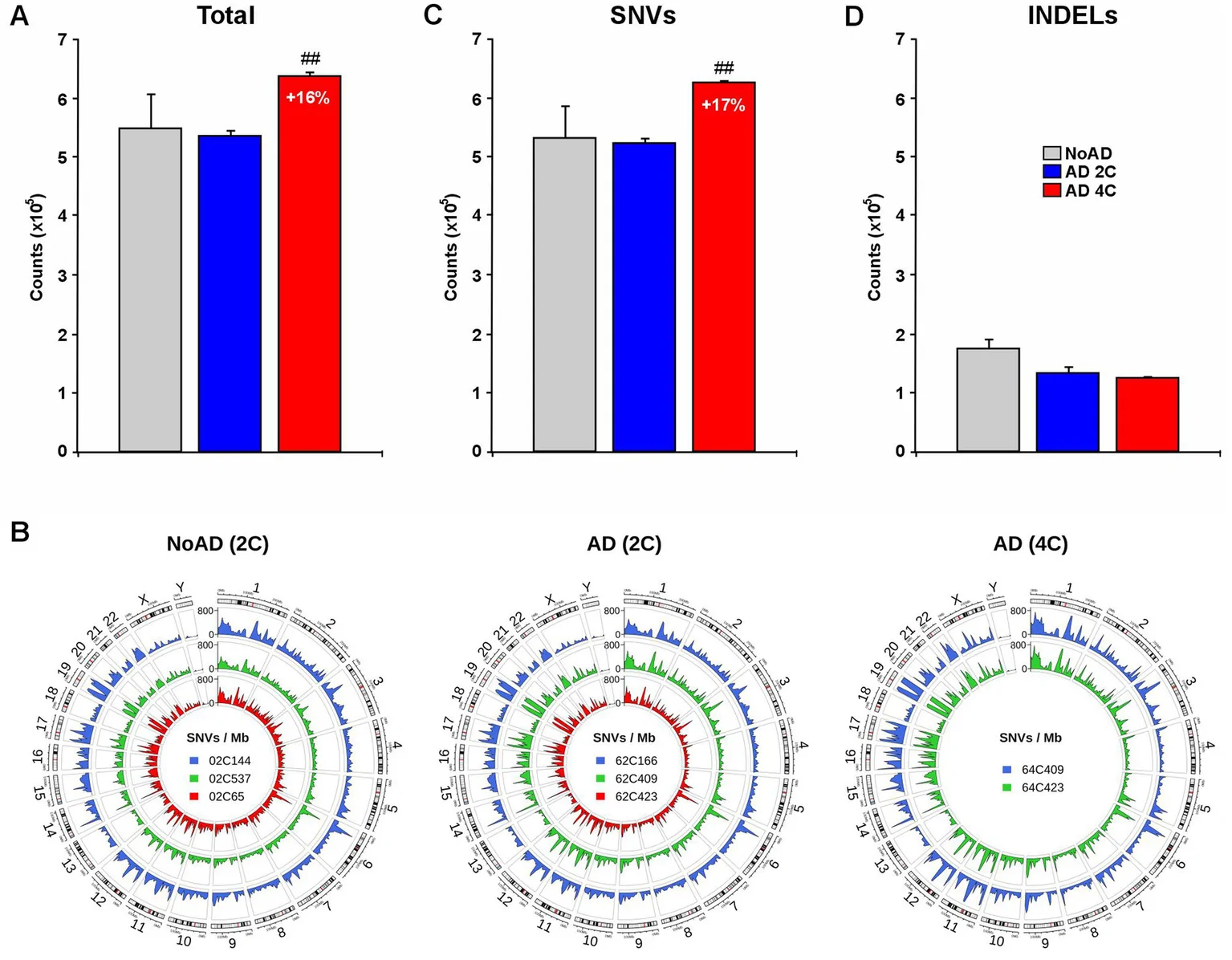
Total mutation counts in diploid neurons from control (NoAD) and AD patients (AD 2C) as well as in tetraploid neurons from AD patients (AD 4C). **(A)** Total somatic mutations observed in NoAD diploid neurons (grey), AD diploid neurons (blue) and AD tetraploid neurons (red). **(B)** Total sSNVs observed in NoAD diploid neurons (grey), AD diploid neurons (blue) and AD tetraploid neurons (red). **(C)** Total INDELs observed in NoAD diploid neurons (grey), AD diploid neurons (blue) and AD tetraploid neurons (red). **(D)** Circos plots illustrating the chromosomal distribution of somatic mutations (quantitative scale bars for variant density, along with normalized mutation counts per Mb) observed in NoAD diploid neurons, AD diploid neurons and AD tetraploid neurons from the individuals analyzed in this study. ^##^*p* < 0.01 (compared with the AD 2C group) (Student’s *t* test).

### 3.2 Specific increase of de novo-generated SNVs in tetraploid neurons from AD patients

We determined the distribution of allele frequencies (AF) at which different SNVs were present in the genomic DNAs. This analysis revealed one major peak of SNVs with low frequency (AF < 0.1) and two minor peaks at AF = 0.5 and AF = 1.0 in the three neuronal populations (Figure 2A). We interpreted these two peaks as germline SNVs (gSNVs) (or allelic variants) in heterozygosity and homozygosity, respectively. In contrast, the major peak with AF < 0.1 represents sSNVs generated de novo as the brain ages (Lodato et al., 2018) and/or when it undergoes neurodegeneration (Miller et al., 2022; Parcerisas et al., 2014; Park et al., 2019). This interpretation is consistent with the results obtained from the analysis of SNVs within the same AD individuals, classified as either common to both diploid and tetraploid neurons (i.e., germline variants) or specific to either diploid or tetraploid neurons (i.e., somatic mutations) (Supplementary Figure S1). Common SNVs showed conspicuous peaks with AF = 0.5 and AF = 1.0, plus an additional peak of SNVs at low frequency, likely derived from clonal somatic mutations during early development (Miller et al., 2021). Early developmental clonal somatic mosaicism can also generate variants with AF > 0.25 (Waldvogel et al., 2024). To assess whether some SNVs classified as gSNVs (i.e., variants with AF > 0.25) could represent developmental somatic mosaic variants, we compared these variants with those reported by Bae et al. (2018) associated with the first five post-zygotic cleavages in the forebrain of three human fetuses. No overlap was observed between the two datasets, and therefore no gSNVs were removed based on this comparison. This finding is consistent with the interpretation that the high-frequency variants identified in our study are predominantly germline variants and suggests that the contribution of the early developmental brain mosaic variants reported by Bae et al. (2018) to our gSNV set is unlikely to be substantial. In contrast, SNVs unique to diploid or tetraploid neurons were dramatically enriched for AF < 0.1.

**Figure 2:**
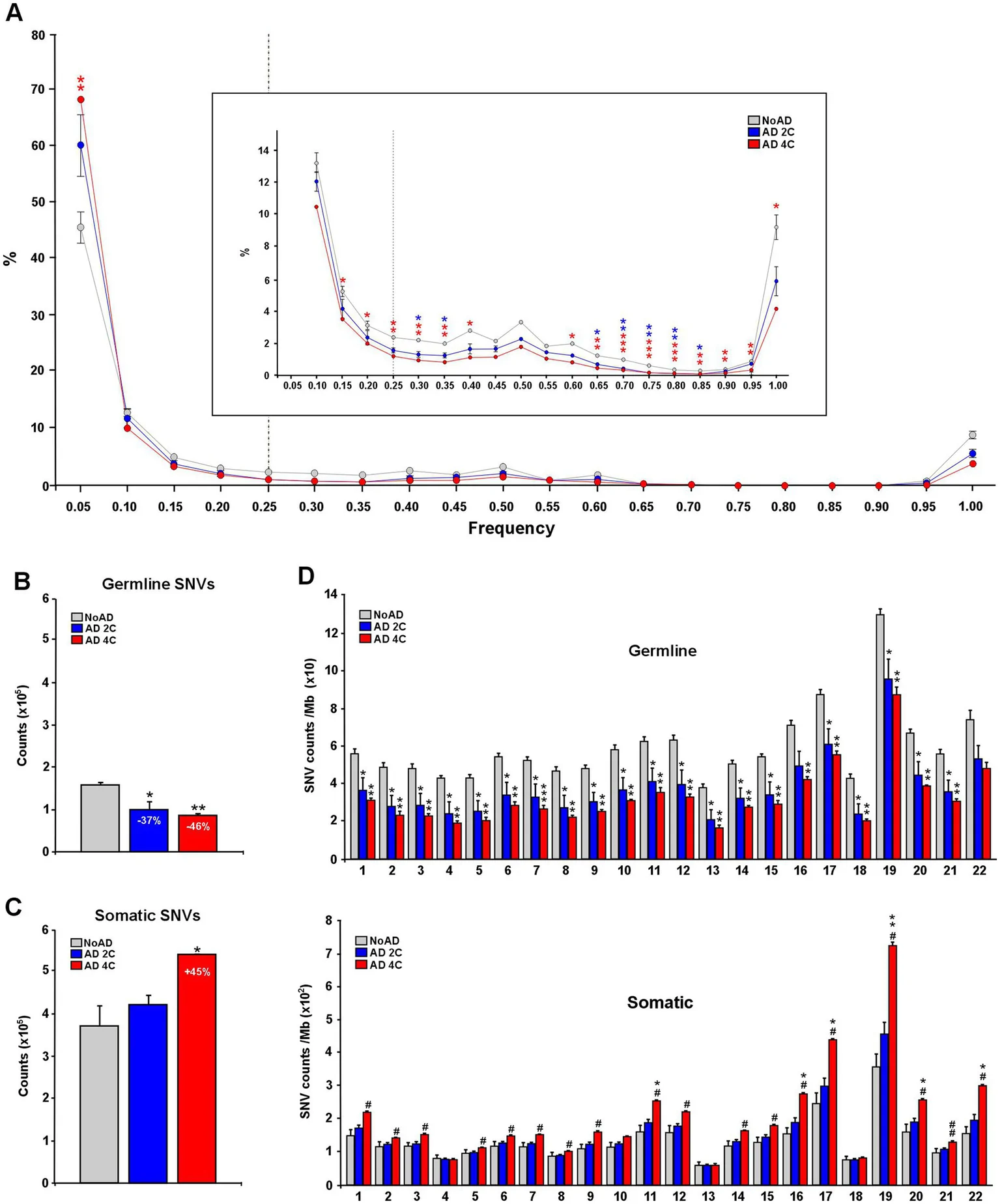
SNVs from germline and somatic origin. **(A)** Relative frequency distribution of the SNVs in NoAD diploid neurons (grey), AD diploid neurons (blue) and AD tetraploid neurons (red). Dotted line represent the threshold that was used to differentiate mutations from germline (right) and somatic (left) origin. Box: zoom of the graph indicating percentages ranging from 0 to 14. **(B)** Germline SNVs observed in NoAD diploid neurons (grey), AD diploid neurons (blue) and AD tetraploid neurons (red). **(C)** Somatic SNVs observed in NoAD diploid neurons (grey), AD diploid neurons (blue) and AD tetraploid neurons (red). **(D)** Germline (top) and somatic (bottom) SNV counts per chromosomal Mb. Numbers identify the chromosomes. *p < 0.05, **p < 0.01; ***p < 0.001 (compared with the NoAD group), ^#^*p* < 0.05, ^##^*p* < 0.01 (compared with the AD 2C group) (Student’s *t* test).

Based on these findings, we set AF = 0.25 as the threshold between SNVs that are present at low frequency (AF < 0.25), which would represent sSNVs, and those that appear at high frequency (AF > 0.25), which would mostly be gSNVs. This setting is consistent with the presence of a substantial proportion of high-frequency SNVs (i.e., gSNVs) in databases containing somatic nucleotide polymorphisms, including the Catalogue of Somatic Mutations in Cancer (COSMIC^7^), the ICGC Data Portal^8^, and the reference single nucleotide polymorphism database (dbSNP^9^), while most low-frequency SNVs (i.e., sSNVs) were absent from these databases (Supplementary Figure S2).

As expected, the absolute number of gSNVs was similar in both diploid and tetraploid neurons from patients with AD (Figure 2B). Nevertheless, this number was significantly greater in NoAD neurons (Figure 2B), which might be interpreted in terms of the genetic predisposition for the etiology of AD (Bellenguez et al., 2022), suggesting that a reduced proportion of gSNVs would be linked to it. Interestingly, this analysis also indicated that the proportion of sSNVs significantly increased (45%) in tetraploid neurons from AD patients compared with diploid neurons from NoAD individuals (Figure 2C).

SNVs differed among the specific chromosomes (Figure 2D). sSNVs, especially those observed in tetraploid neurons, were enriched on chromosome 19, whereas three chromosomes (4, 13, and 18) showed low levels of sSNVs per Mb, which were equivalent in all neuronal types. This contrasts with the distribution of gSNVs, as these mutations showed decreased numbers per Mb in AD-derived neurons, independent of the chromosome that was analyzed.

To verify the specificity of the increase in sSNVs in tetraploid neurons, we performed a similar study on INDELs. INDELs also showed one major peak with low frequency (AF < 0.1) (i.e., somatic INDELs) and two minor peaks at AF = 0.5 and AF = 1.0 (germline INDELs in heterozygosity and homozygosity, respectively) in the three neuronal populations (Supplementary Figure S3A). In addition, another peak of uncertain nature was observed at AF = 0.4. In all cases, the absolute number of germline INDELs did not significantly differ between diploid and tetraploid neurons from AD patients (Supplementary Figure S3B); however, as in the case of gSNVs, this number increased in NoAD neurons (Supplementary Figure S3B). In contrast, the number of somatic INDELs was similar in the three types of neurons analyzed (Supplementary Figure S3C), thus providing evidence that the increase in sSNVs observed in the tetraploid neurons (Figure 2C) was specific for this latter type of mutation.

Quantitative differences between somatic INDELs and sSNVs were also observed when their chromosomal distribution was considered, further supporting the specificity of the observations for sSNVs. In contrast to sSNVs, which were significantly increased in most chromosomes (Figure 2D), the absolute number of somatic INDELs per Mb was either decreased or maintained in most chromosomes, except for chromosome 19, in which a statistically significant increase in the number of somatic INDELs per Mb was observed (right panel in Supplementary Figure S3D). The analysis performed with germline INDELs indicated that their chromosomal distribution was similar to that observed for gSNVs (left panel, Supplementary Figure S3D).

Overall, these findings support the hypothesis that NT is specifically associated with an increase in sSNVs (i.e., somatic mutations characterized by single nucleotide changes).

### 3.3 Selective genome localization and specific function of sSNVs in tetraploid neurons from AD patients

The genomic region and functional protein effect of the identified SNVs were studied, considering whether they were of germline or somatic origin. We found that sSNVs were enriched compared to gSNVs in the coding regions of all types of neurons (Supplementary Figure S4), and the proportion of sSNVs present in introns from tetraploid neurons was significantly reduced (Supplementary Figure S5A). Moreover, all neurons from AD patients showed a significant increase in sSNVs located in 5′-untranslated regions (UTRs) (Supplementary Figure S5B), while the presence of these mutations in promoters was significantly increased in tetraploid neurons (Supplementary Figure S5B). gSNVs were slightly increased in coding regions (Supplementary Figure S4), exons (Supplementary Figure S5A), and promoters (Supplementary Figure S5B) in AD neurons compared to NoAD neurons, suggesting that these mutations could participate in the known genetic predisposition of AD patients (Bellenguez et al., 2022). In accordance with the reduced proportion of sSNVs present in introns, tetraploid neurons contained significantly fewer sSNVs in intron acceptor sequences (Supplementary Figure S5C). These observations are consistent with a reduced functional effect on the splicing of both germline and sSNVs in AD neurons (Supplementary Figure S6A). A similar situation was found for stop-gaining mutations, which dramatically decreased among sSNVs in neurons from individuals with AD, an effect that seems to be potentiated in tetraploid neurons (Supplementary Figure S6B). In addition, all types of neurons showed an enrichment of missense sSNVs compared to the proportion of missense gSNVs in these cells, an enrichment that was significantly enhanced in tetraploid neurons (Supplementary Figure S6C). Overall, these findings suggest that SNT is associated with sSNVs involved in protein malfunction due to missense mutations while largely maintaining intact protein functional domains, as both altered splicing and stop-gaining mutations are reduced.

### 3.4 sSNVs from tetraploid neurons are enriched in T to C (T>C) transitions

Lodato et al. (2018)demonstrated that somatic mutations, mostly T > C (or A > G in the DNA complementary strand) variants, accumulate in neurons during aging. Therefore, we identified the types of mutations that can be found in the studied neuronal types. This analysis indicated that T > C transitions were strongly enriched in tetraploid neurons, mostly within the pool of sSNVs (Figure 3). This result was confirmed when SNVs common to diploid and tetraploid neurons and specific to either diploid or tetraploid neurons were studied. A dramatic increase in T > C transitions was specifically observed in SNVs specific to tetraploid neurons (Supplementary Figure S7).

**Figure 3:**
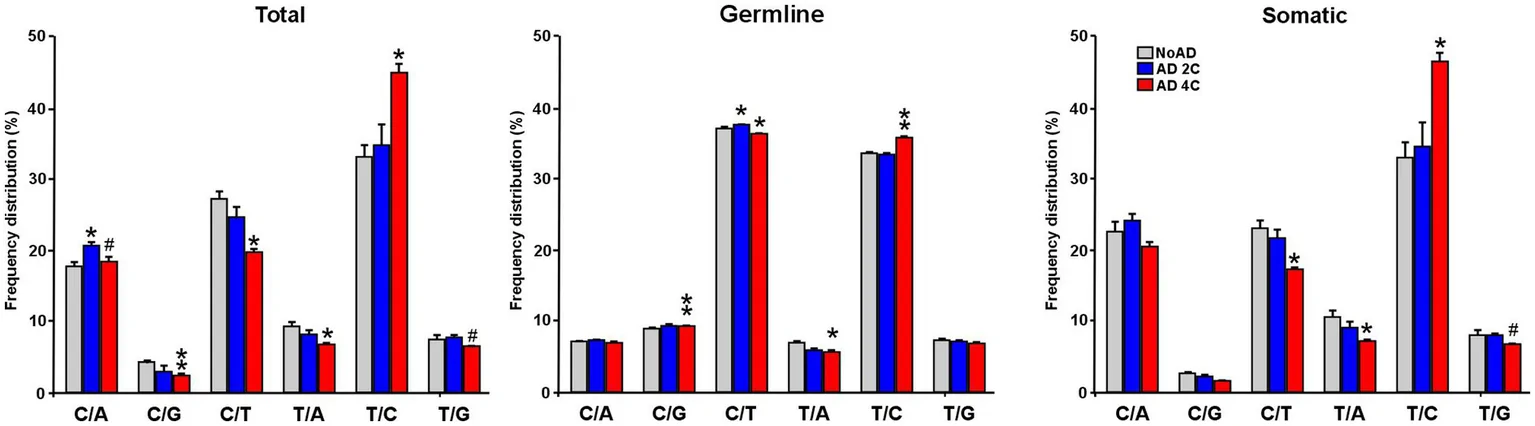
Percentage of the identified sSNVs in diploid neurons from NoAD individuals and AD patients as well as in tetraploid neurons from AD patients. Percentage of the indicated sSNVs observed in NoAD diploid neurons (grey), AD diploid neurons (blue) and AD tetraploid neurons (red). C/A (C > A, G > T), C/G (C > G, G > C), C/T (C > T, G > A), T/A (T > A, A > T), T/C (T > C, A > G), T/G (T > G, A > C). *p < 0.05, **p < 0.01 (compared with the NoAD group), ^#^*p* < 0.05 (compared with the AD 2C group) (Student’s *t* test).

### 3.5 Accumulation of ultra-low frequency sSNVs and enrichment in T>C transitions in AD-associated tetraploid neurons

As shown above, tetraploid neurons obtained from patients with AD contained a statistically significant increase in sSNVs, largely associated with T > C transitions. Because the algorithm used for variant calling in this analysis (VarScan 2) has limitations in the detection of ultra-low frequency variants (Koboldt et al., 2012), we decided to repeat the analysis using the LoFreq variant caller (Wilm et al., 2012). This algorithm, which can uncover SNVs with frequencies as low as 0.05%–1% (Wilm et al., 2012), was used to identify SNVs with AF < 0.05 (i.e., sSNVs). This analysis confirmed that sSNVs were significantly enriched in tetraploid neurons compared to diploid neurons, while no statistically significant differences were observed when diploid neurons from control individuals were compared to those from AD patients (Figure 4A). This analysis also indicated that the frequency of sSNVs in the tetraploid neurons was mostly in the range of 0.005–0.03, with a strong peak at AF = 0.01 (Figure 4B). In contrast, as proof of specificity, the sSNVs present in diploid neurons showed a much flatter profile, with a peak at AF = 0.02 (Figure 4B).

**Figure 4:**
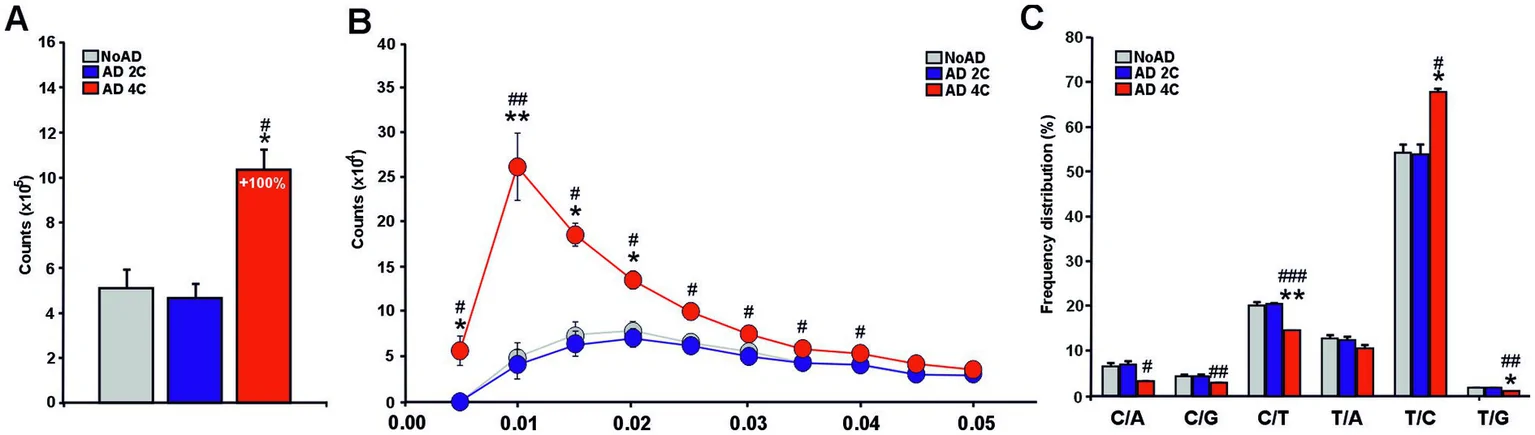
Identification of SNVs with AF < 0.05 (i.e., sSNVs) using the LoFreq variant caller. **(A)** Somatic SNVs identified with the LoFreq variant caller (AF < 0.05) in NoAD diploid neurons (grey), AD diploid neurons (blue) and AD tetraploid neurons (red). **(B)** Distribution of frequencies of the SNVs identified with the LoFreq variant caller (AF < 0.05) in NoAD diploid neurons (grey), AD diploid neurons (blue) and AD tetraploid neurons (red). **(C)** Percentage of the indicated sSNVs, identified with the LoFreq variant caller (AF < 0.05, QUAL > 30) in NoAD diploid neurons (grey), AD diploid neurons (blue) and AD tetraploid neurons (red). C/A (C > A, G > T), C/G (C > G, G > C), C/T (C > T, G > A), T/A (T > A, A > T), T/C (T > C, A > G), T/G (T > G, A > C). *p < 0.05, **p < 0.01 (compared with the NoAD group), ^#^*p* < 0.05, ^##^*p* < 0.01, ^###^*p* < 0.001 (compared with the AD 2C group) (Student’s *t* test).

Analysis with the LoFreq variant caller of the SNVs that are present in the different neuronal populations confirmed our previous finding that T > C transitions are significantly enriched in tetraploid neurons at the expense of other types of single nucleotide mutations (Figure 4C).

To further characterize these very low frequent variants, we performed mutational-signature analysis (Supplementary Figure S8A). The observed spectra showed high cosine similarity to established single-base substitution (SBS) reference signatures (Supplementary Figure S8B,C), with SBS123 and SBS102 accounting for the major contributions in most samples, together with contributions from SBS116 and, in some samples, SBS5. These reference signatures were established from large-scale WGS datasets generated independently of the PicoPLEX amplification procedure used here. In particular, SBS123 and SBS102 were identified by Degasperi et al. (2022) from 18,640 cancer WGS samples, and SBS102 is represented in the Signal database as SBS102 (Degasperi et al., 2022). Thus, the substitution spectrum of neuronal sSNVs showed similarity to mutational patterns independently identified in non-PicoPLEX WGS datasets.

SBS123 contributed to the mutational profiles of all analyzed samples (Supplementary Figure S8B). Although this signature was not specific to the tetraploid state, its relative contribution was higher in AD 4C samples, accounting for 64–66% of fitted mutations, compared with 49%–57% in NoAD samples and 52–59% in AD 2C samples. The difference between NoAD and AD 4C samples was significant by Tukey’s HSD post-hoc test (Supplementary Figure S8B).

The secondary signature profile also differed between groups. In AD 4C samples (409 and 423), SBS116 contributed up to 21% of fitted mutations, while SBS5 contributed 13% and 14%, respectively. In contrast, SBS102 was the predominant secondary signature in all AD 2C samples, accounting for 41%–45% of fitted mutations (Supplementary Figure S8B). SBS5 and SBS116 were not consistently detected in the diploid groups.

The fitted signatures showed high similarity to the observed mutational profiles, with cosine similarity values ranging from 0.938 to 0.958 across all samples, supporting the robustness of the signature fitting (Supplementary Figure S8B).

Overall, tetraploidization in AD neurons was associated with a shift in the relative contribution and composition of mutational signatures rather than the emergence of a tetraploidy-specific signature. This shift was characterized by an increased relative contribution of SBS123 and a distinct secondary signature profile in AD-4C neurons.

### 3.6 Enrichment of sSNVs in neurodegenerative disease-associated genes

Previous reports have indicated that somatic variants in autosomal dominant genes known to be involved in AD can be associated with the etiology of this disease (Nicolas et al., 2018; Park et al., 2019). Therefore, we explored whether sSNVs in AD risk genes (see list in Supplementary Table S2) could be enriched in tetraploid neurons. As a control, we used a list of Parkinson’s disease (PD)-related genes (see list in Supplementary Table S2).

This analysis indicated that tetraploid neurons display a strong increase of total SNVs in AD-risk genes (27% increase as compared to 16% in the whole genome; Figure 1A), showing a greater increase (59% as compared to 45% in the whole genome; Figure 2C) when sSNVs are considered (Figure 5A). This contrasts with the 40% reduction in gSNVs present in AD-risk genes from tetraploid neurons (Figure 5A).

**Figure 5:**
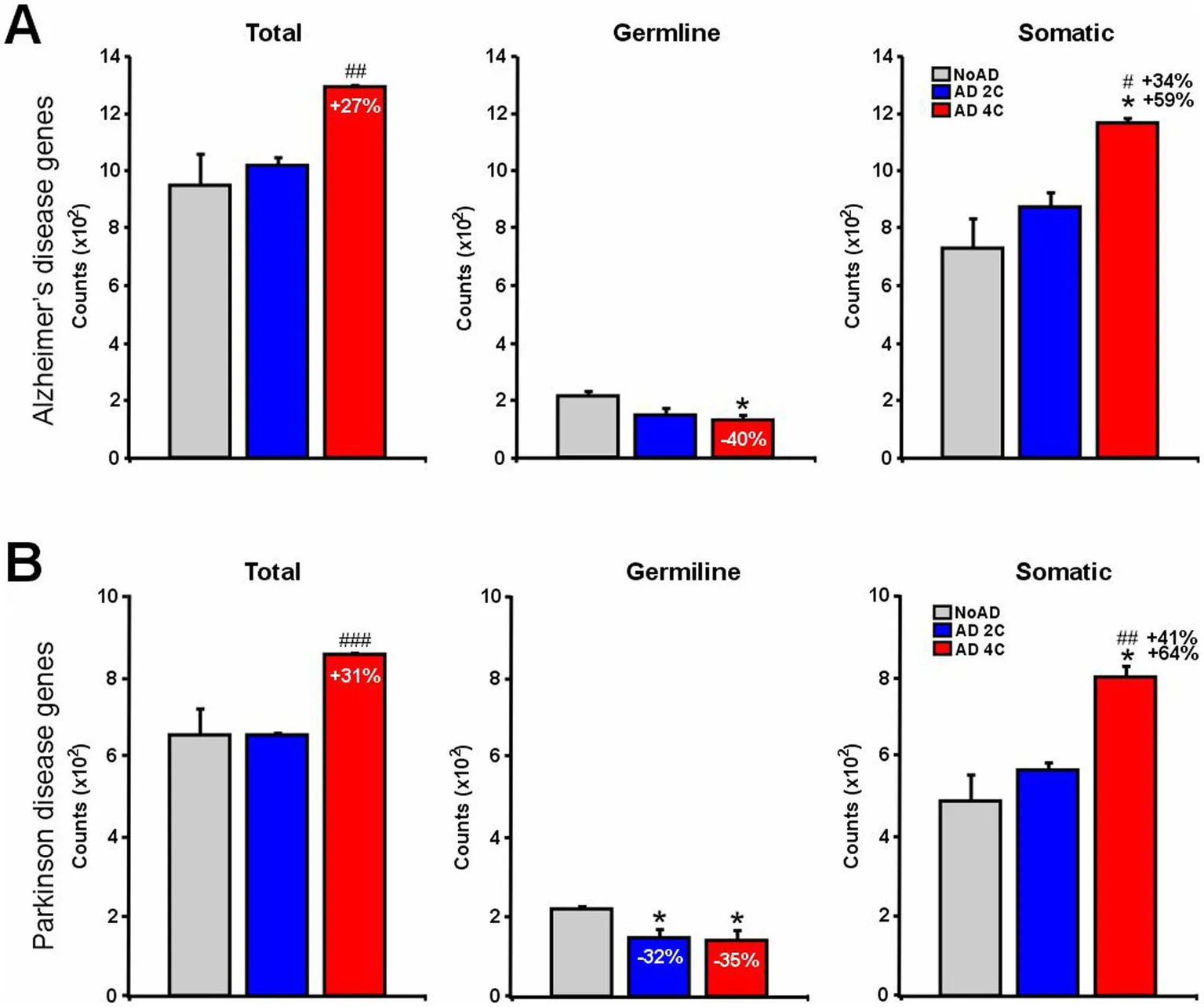
sSNVs counts in AD- and PD-risk gene sets of neurons isolated from NoAD individuals and AD patients as well as in tetraploid neurons from AD patients. **(A)** Total (left), germline (middle) and somatic (right) sSNVs observed in AD-risk genes from NoAD diploid neurons (grey), AD diploid neurons (blue) and AD tetraploid neurons (red). **(B)** As is A for PD genes. *p < 0.05 (compared with the NoAD group), ^#^*p* < 0.05, ^##^*p* < 0.01, ^###^*p* < 0.001 (compared with the AD 2C group) (Student’s t test).

Interestingly, some differences were detected when AD-risk genes were considered individually. While ABCA7, APOE, BIN1, CD33, EPHA1, MAPT, PSEN2, SMARCA4, and PLD3 showed a significant increase in the number of sSNVs from tetraploid neurons, CD2AP and PSEN1 showed the opposite effect (Supplementary Figure S9).

Surprisingly, PD risk genes (Figure 5B) also showed an increase in SNVs (31%), which was enhanced in sSNVs (64% increase), whereas gSNVs were also reduced (35%), as occurred with AD-risk genes.

### 3.7 Enrichment of sSNVs in oxidative stress-associated genes

To begin to understand the possible molecular mechanisms involved in AD-associated NT, we focused on oxidative stress-associated genes (see Supplementary Table S2 for a list of these genes), since oxidative stress is a known stressor participating in AD (Butterfield and Halliwell, 2019).

We found that tetraploid neurons accumulated SNVs in genes involved in antioxidant activity (38%) (Figure 6A), response to reactive oxygen species (ROS) (31%) (Figure 6B), hydrogen peroxide activity (29%) (Figure 6C), and oxidative stress (26%) (Figure 6D), while no statistically significant increase was detected in the proportion of SNVs affecting genes involved in both cell death by oxidative stress (Figure 6E) and superoxide dismutase (SOD) activity (Figure 6F) when tetraploid and diploid neurons were compared. This analysis also indicated a significant increase in sSNVs affecting a subgroup of oxidative stress-associated genes, which was higher than that observed in the global genome (45%, Figure 2C). In this regard, 64% (Figure 6A) and 61% (Figure 6B) of sSNVs were detected in genes involved in antioxidant activity and ROS response, respectively. In contrast, only 36% (Figure 6C) and 33% increase (Figure 6D) of sSNVs was detected in genes involved in hydrogen peroxide activity and oxidative stress, respectively, while no statistically significant increase was detected in the proportion of total sSNVs affecting genes involved in both cell death by oxidative stress (Figure 6E) and superoxide dismutase (SOD) activity (Figure 6F). Interestingly, with the exception of SOD genes, the presence of gSNVs in genes involved in oxidative stress was significantly reduced in the tetraploid neuron population (Figure 6A–E).

**Figure 6:**
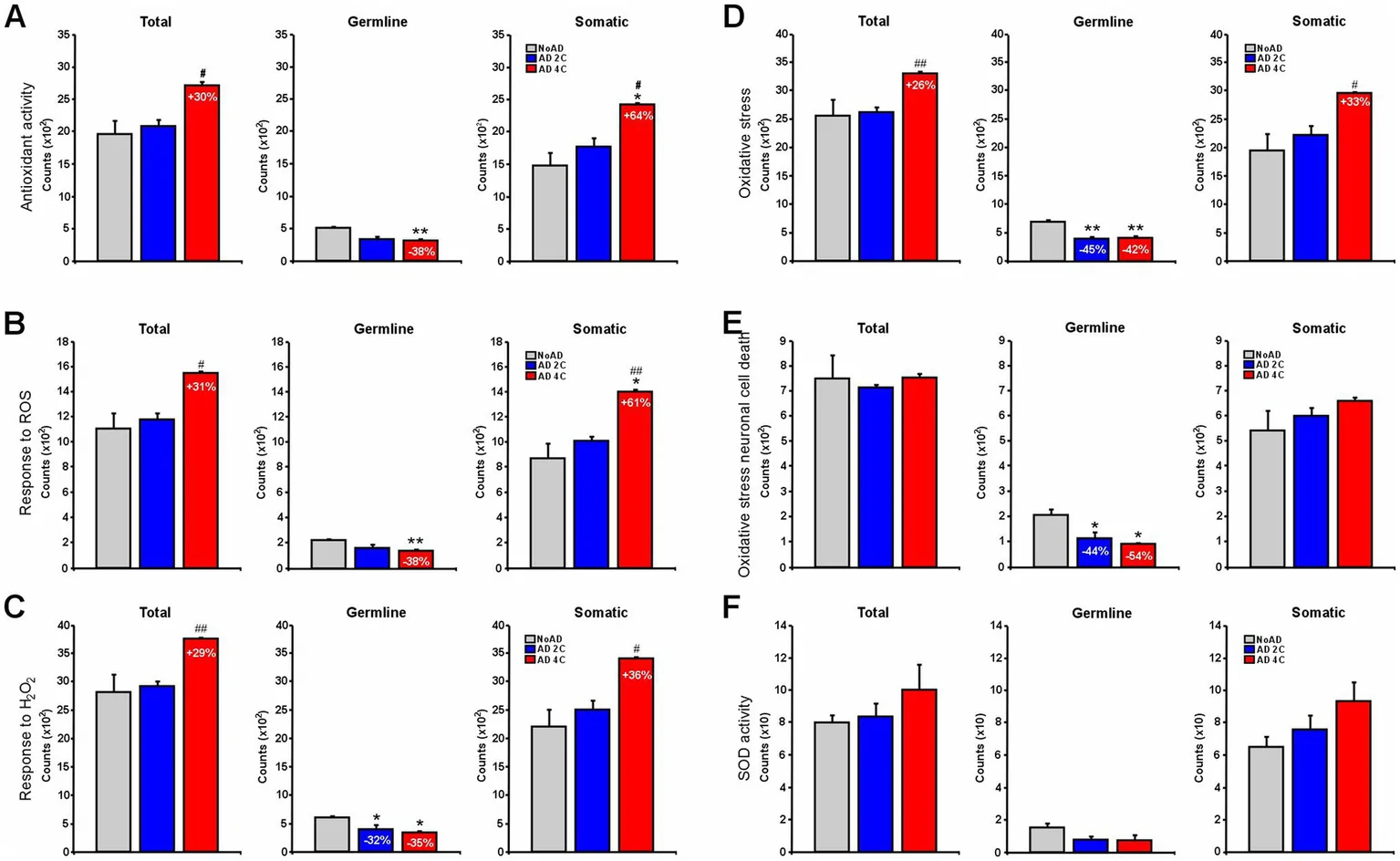
sSNVs counts in oxidative stress-associated gene sets of neurons isolated from NoAD individuals and AD patients as well as in tetraploid neurons from AD patients. **(A)** Total (left), germline (middle) and somatic (right) sSNVs observed in antioxidant activity genes from NoAD diploid neurons (grey), AD diploid neurons (blue) and AD tetraploid neurons (red). **(B)** As is A for response to reactive oxygen species genes. **(C)** As is A for hydrogen peroxide genes. **(D)** As is A for oxidative stress genes. **(E)** As is A for oxidative stress neuronal cell death genes. **(F)** As is A for SOD activity genes. *p < 0.05, **p < 0.01 (compared with the NoAD group), ^#^*p* < 0.05, ^##^*p* < 0.01 (compared with the AD 2C group) (Student’s *t* test).

Overall, these findings may indicate that oxidative stress-associated sSNVs involved in the response to oxidation and ROS accumulation may participate in NT generation in the AD brain.

### 3.8 Enrichment of sSNVs in DNA repair-associated genes

We found that SNVs accumulated (31% increase) in DNA repair genes (see list in Supplementary Table S2) from tetraploid neurons (Figure 7A), which represented a 64% increase in sSNVs compared to a 45% increase in sSNVs in the global genome (Figure 2C). In contrast, gSNVs decreased by 35% in these neurons (Figure 7A).

**Figure 7:**
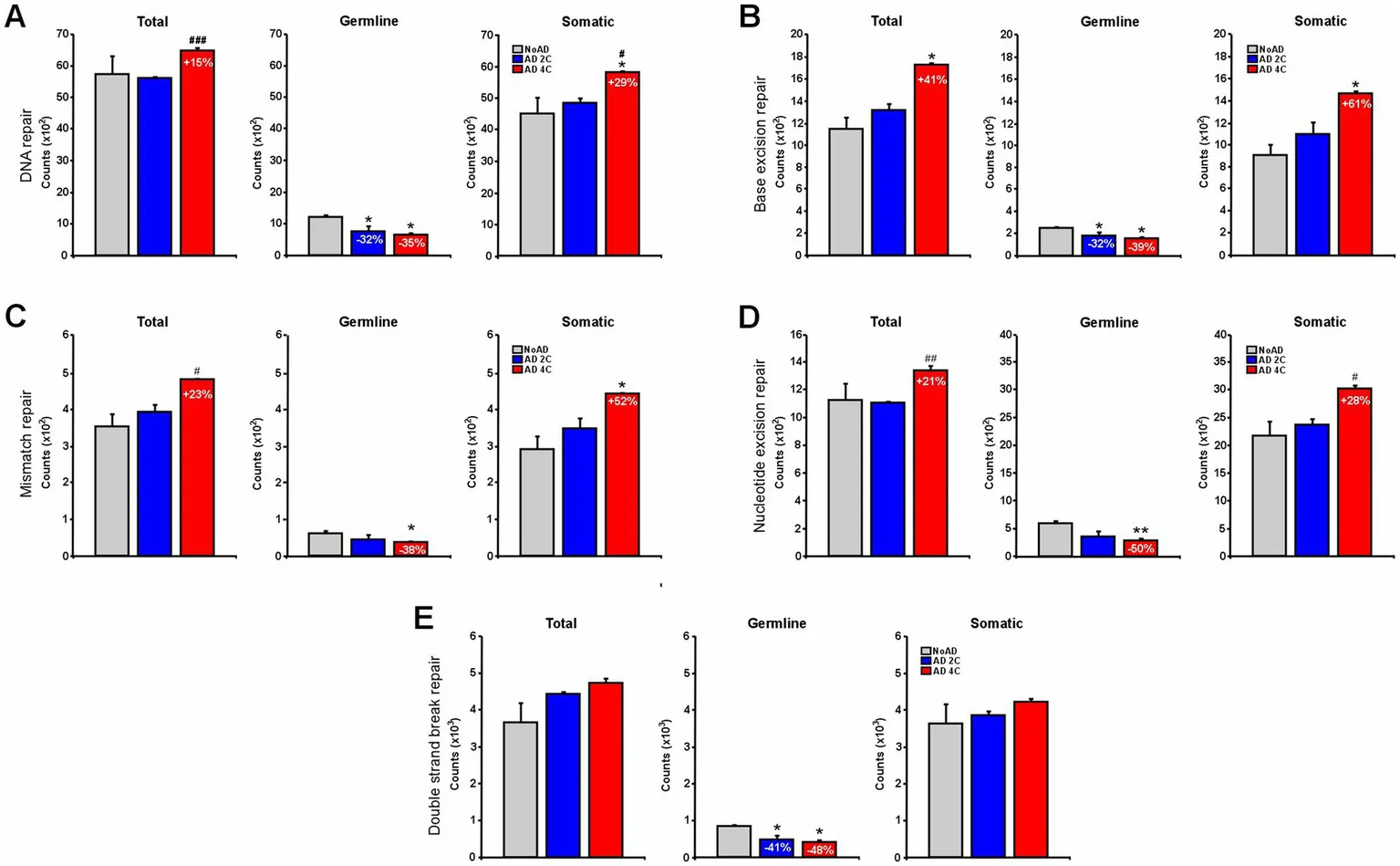
sSNVs counts in DNA repair-associated gene sets of neurons isolated from NoAD individuals and AD patients as well as in tetraploid neurons from AD patients. **(A)** Total (left), germline (middle) and somatic (right) sSNVs observed in DNA repair genes from NoAD diploid neurons (grey), AD diploid neurons (blue) and AD tetraploid neurons (red). **(B)** As is A for base excision repair genes. **(C)** As is A for mismatch repair genes. **(D)** As is A for nucleotide excision repair genes. **(E)** As is A for double strand break repair genes. *p < 0.05, **p < 0.01 (compared with the NoAD group), ^#^*p* < 0.05, ^##^*p* < 0.01, ^###^*p* < 0.001 (compared with the AD 2C group) (Student’s *t* test).

To explore whether this finding could be extended to all types of genes involved in DNA repair, we focused on genes involved in base excision, mismatch, nucleotide excision, and double-strand break repair (see Supplementary Table S2 for a complete list of these genes). This analysis indicated that tetraploid neurons displayed a significant increase in total SNVs in all these genes, except those involved in double-strand break repair (Figure 7B–E). For sSNVs, the increase in tetraploid neurons of genes involved in base excision, mismatch, and nucleotide excision was 61% (Figure 7B), 52% (Figure 7C), and 28% (Figure 7D), respectively, while no significant increase of sSNVs was observed in genes regulating double-strand breaks (Figure 7E). As described above for other gene sets, gSNVs were significantly reduced in tetraploid neurons for all DNA repair-associated genes in the present study.

Overall, this finding is consistent with the hypothesis that stochastic mutations in DNA repair genes, due to oxidative stress or other aging-associated mechanisms, facilitate the accumulation of additional somatic mutations due to the lack of capacity to repair DNA in these cells. This likely facilitates SNT induction.

### 3.9 Absence of enrichment of sSNVs in cancer-related and senescence-related genes

We have previously reported that the expression of oncogenes in the absence of p53 activity facilitates cell cycle re-entry in neurons and the survival of generated hyperploid neurons (Barrio-Alonso et al., 2018, 2020; Walton et al., 2019). Therefore, we hypothesized that tetraploid neurons contain an excess of sSNVs in cancer-related (CR) genes and that the pathogenicity of these mutations is greater than that of other genes. To test these hypotheses, we focused on different proto-oncogenes and tumor suppressor genes (see Supplementary Table S2 for a complete list of genes). This analysis indicated that the proportion of CR gene-specific sSNVs was significantly higher (23%) in tetraploid neurons than in diploid neurons in both AD patients and NoAD individuals (Figure 8B). As a control, we focused on the 173 genes analyzed by Lodato et al. (2018), which showed a slightly lower proportion of sSNVs in tetraploid neurons than in diploid neurons (20%) (Figure 8A). In contrast, the increase in sSNVs observed in CR genes from tetraploid neurons was lower than that observed in genes from the global genome (Figure 2C). Therefore, the accumulation of CR gene mutations in tetraploid neurons does not appear to be greater than that observed in control genes.

**Figure 8:**
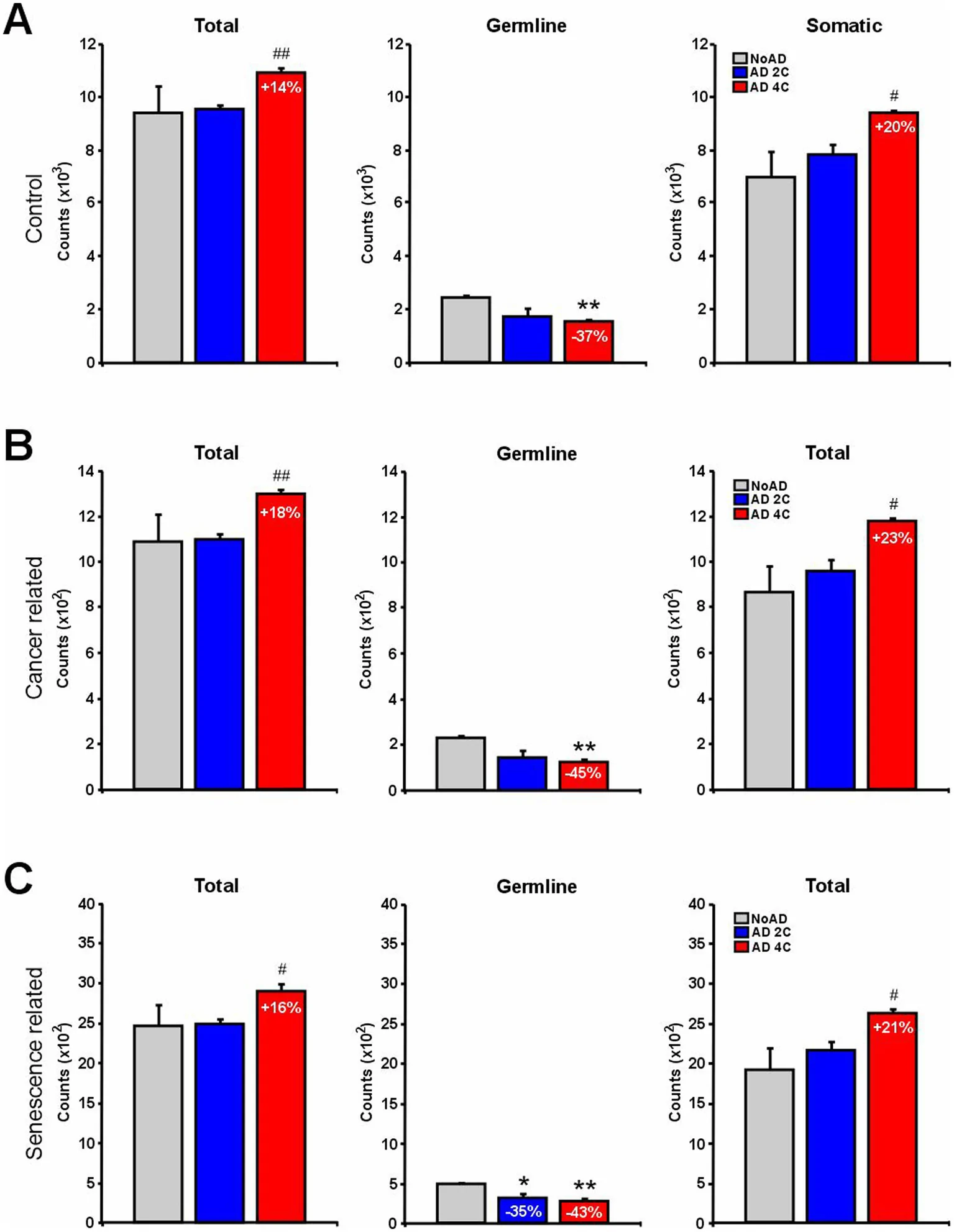
sSNVs counts in CR and SR gene sets of neurons isolated from NoAD individuals and AD patients as well as in tetraploid neurons from AD patients. **(A)** Total (left), germline (middle) and somatic (right) sSNVs observed in control genes from NoAD diploid neurons (grey), AD diploid neurons (blue) and AD tetraploid neurons (red). **(B)** As is A for CR genes. **(C)** As is A for SR genes. *p < 0.05, **p < 0.01 (compared with the NoAD group), ^#^*p* < 0.05, ^##^*p* < 0.01 (compared with the AD 2C group) (Student’s *t* test).

We also focused on senescence-related (SR) genes (Dehkordi et al., 2021) (see Supplementary Table S2 for a complete list of these genes) and found that the proportion of SR gene-specific sSNVs was significantly higher (21%) in tetraploid neurons than in diploid neurons from AD patients (Figure 8C). This proportion of SR gene-specific sSNVs was similar to that found in the control gene sets (Figure 8A) and lower than that observed in genes from the global genome (Figure 2C). Therefore, as in the case of CR genes, the accumulation of SR gene mutations in tetraploid neurons is not enriched compared to that observed in control genes.

### 3.10 Cancer-related and senescence-related gene-associated sSNVs show high MPS

To study the expected molecular pathogenicity of the proteins encoded by the genes that accumulate sSNVs in tetraploid neurons, we used the DG prediction score. This score is calculated from 12 functional prediction algorithms, with 0 being the minimum (least pathogenic variant) and 5 being the maximum (most pathogenic variant). Then, the frequency distribution of the prediction score (FDPS) was estimated in control (Lodato et al., 2018), CR, and SR (Dehkordi et al., 2021) genes, and the regression slope of the FDPS frequency distribution was used as a measure of the MPS (Supplementary Figure S10) (i.e., the greater the slope, the stronger the MPS). When the FDPS for the CR and SR gene-specific pools of SNVs were evaluated in tetraploid neurons, a significant enrichment of highly pathogenic SNVs was observed for both sets of genes compared to all sSNVs, evaluated as the statistical difference between the linear regression slope for the tested gene sets compared to the regression slope for the control genes (see López-Sanchez et al., 2021) (Table 1). This indicates that CR and SR genes are highly susceptible to mutations in the pathogenic form. This may be involved in SNT, since the absolute proportion of sSNVs in both sets of genes was higher in tetraploid neurons (Figure 2C).

**Table 1:** FPDS regression slope comparison in sSNVs from tetraploid neurons of AD patients.

Gene function	Set of genes	All SNVs (regression slope: −3.4993; *R*: 0.901)				
		Reg. slope	*R*	DF	*t*-value	Probability
Generic	Control	−4.2915	0.8983	16	0.8324	0.4128
Cancer	Cancer	0.1867	0.2052	16	5.4579	**0.0001**
Oxidative stress	SOD activ.	1.4815	0.3708	16	3.2900	**0.0046**
	Neuron death	−1.7427	0.4516	16	1.2264	0.2378
	Oxidat. stress	0.8768	0.5680	16	4.9882	**0.0001**
	Hydrogen perox.	−0.1076	0.0938	16	4.7029	**0.0001**
	Reactiv. oxygen	−0.8786	0.1931	16	1.5003	0.1530
	Antiox. activ.	−2.4929	0.6920	16	0.8439	0.4111
DNA repair	DSBR	−5.0157	0.8994	16	1.4467	0.1673
	BER	−0.3989	0.2186	16	3.7771	**0.0017**
	MMR	2.0602	0.6070	16	5.0228	**0.0001**
	NER	3.3651	0.8201	16	6.8094	**4E-06**
	DNA repair	−1.9604	0.5529	16	0.7508	0.4637
Senescence	Senescence	−1.0193	0.6329	16	3.3331	**0.0026**
Neurodegeneration	Alzheimer	−1.3476	0.8320	16	1.1932	0.2502
	Parkinson	−3.588	0.8628	16	0.0464	0.9635

Statistical significance of differences between two linear regression slopes (All SNVs vs. the indicated set of genes) was evaluated using a *t* test based on the slope, standard error, and sample size (López-Sánchez et al., 2021). Reg.: regression. R: Pearson’s r correlation value. DF: Degrees of freedom. Bold: statistically significant differences.

### 3.11 Variable MPS of other sSNVs

We also evaluated the FDPS of sSNVs in tetraploid neurons from other gene groups by applying DG prediction scores. This analysis indicated that oxidative stress-associated genes showed a variable degree of MPS in tetraploid neurons, with genes involved in SOD activity, oxidative stress, and hydrogen peroxide activity displaying a higher degree of MPS (Supplementary Figure S10), which was statistically significant compared to all sSNVs (Table 1).

DNA repair genes, including those involved in base excision, mismatch, and nucleotide excision, showed a dramatic increase in MPS (Supplementary Figure S10), which resulted in significant differences from all sSNVs (Table 1). This contrasted with genes involved in double-strand break repair, which in tetraploid neurons showed a low degree of MPS (consistent with the non-significant increase in sSNVs in these genes). The regression slopes of these genes (Supplementary Figure S10) did not significantly differ from those of the control genes (Table 1).

sSNVs from genes involved in senescence also displayed a greater MPS than those from generic genes in tetraploid neurons (Supplementary Figure S10), an effect that was statistically significant (Table 2021). This supports the notion that the senescent phenotype observed during aging (von Zglinicki et al., 2021) and in AD-affected neurons (Dehkordi et al., 2021) could be caused by DNA damage followed by cell cycle reactivation and tetraploidy in these cells (Jurk et al., 2012).

Finally, this analysis indicated that neither AD- nor PD-associated genes in tetraploid neurons showed increased pathogenic capacity compared to control genes (Supplementary Figure S10; Table 1).

Overall, these findings further support the hypothesis that the lack of capacity for DNA repair facilitates the accumulation of sSNVs in AD neurons in the context of a low capacity to respond to oxidative stress, thus facilitating cancer-like processes in these cells. This mechanism likely participates in the generation of NT in the brains of patients with Alzheimer’s disease.

## 4 Discussion

In this study, we build on our previous work demonstrating the presence and pathological significance of tetraploid neurons in AD (López-Sánchez et al., 2017, 2021, 2022) by providing the first molecular characterization of the somatic mutations present in the genomes of tetraploid neurons isolated from the parietal cortex of patients with AD. We further compare their mutational profiles with those of diploid neurons from both neurologically healthy controls and matched AD individuals. Different criteria were employed to define whether a specific mutation was of somatic origin. We first focused on the frequency at which mutations were detected. In this regard, previous mutational analyses performed in AD neurons focused on either bulk whole exome sequencing from several thousand neurons (Park et al., 2019; Nicolas et al., 2018) or single-cell whole genome sequencing (Li et al., 2023; Miller et al., 2021, 2022). The disadvantage of bulk genome sequencing using genomic DNA from thousands of neurons is the difficulty in detecting low-frequency mutations. We adopted a midway approach between both approaches, as only hundreds of neuronal cells were used for sequencing. This has allowed us to easily identify low-frequency mutations. Since only hundreds of neuronal cell nuclei were whole-genome amplified, those mutations that were present at low frequencies were considered to be of somatic origin. The second criterion for defining whether a defined mutation was somatic was based on whether the mutation was specific for either diploid or tetraploid neurons instead of being common to both cell types. The finding that most low-frequency mutations are unique to diploid or tetraploid neurons supported their somatic origin. Finally, the absence of most low-frequency mutations in existing nucleotide polymorphism databases, known to contain germline mutations, provided additional support for their somatic origin.

An important consideration in this interpretation is that the neuronal genomic DNA was subjected to PicoPLEX whole-genome amplification (WGA) before sequencing. WGA can introduce allelic imbalance, allelic dropout, locus dropout, and polymerase-derived sequence errors, which may affect the observed allele frequencies and therefore the classification of low-frequency variants as sSNVs (de Bourcy et al., 2014; Chen et al., 2018; Estévez-Gómez et al., 2025). We therefore considered the possibility that part of the low-frequency variant population could reflect WGA-associated technical errors rather than biological somatic mutations. To address this concern, we examined the substitution spectra of variants shared between matched 2C and 4C neuronal populations from the same individuals and of variants unique to either neuronal population (Supplementary Figure S7). Notably, T > C substitutions represented approximately 64% of the variants unique to 4C neurons, compared with approximately 37% of variants shared between 2C and 4C neurons and approximately 34% of variants unique to 2C neurons. Thus, the T > C enrichment was particularly pronounced among the 4C-specific variant population rather than being a uniform feature of all variants detected within the same individuals. Although this analysis cannot exclude stochastic WGA-derived errors occurring independently during individual amplification reactions, the distinct substitution spectrum of 4C-specific variants argues against a simple, uniform WGA-associated substitution bias being sufficient to explain the observed 4C-associated mutational pattern.

Two types of somatic mutations were considered in this study: sSNVs and INDELs, although only the former were enriched in tetraploid neurons. This indicates the specificity of the analysis for identifying somatic mutations in these neurons. In general, sSNV mutations were detected in chromosomal regions similar to those of gSNVs, suggesting the presence of mutational hotspots in the genome, as previously demonstrated (Nesta et al., 2021). When gene-including sequences were studied, sSNVs were found to be enriched in coding exons and promoters. As most sSNVs were missense variants with low stop-gaining and reduced effects on splicing, we can conclude that the mutations enriched in tetraploid neurons lead to altered protein expression and protein malfunction while maintaining intact protein functional domains.

The mutational-signature profiles suggest that tetraploidy may be associated with a distinct mutational-signature profile in the AD background. Although SBS123 was the predominant signature across all groups and was therefore not exclusive to the tetraploid state, its relative contribution was consistently higher in the AD 4C samples. Notably, both AD 4C samples displayed a highly similar combination of SBS123, SBS116, and SBS5, whereas the diploid AD samples were characterized predominantly by SBS123 together with SBS102. The reproducibility of this pattern across the two available AD 4C samples is consistent with a shift in the relative contribution of mutational processes associated with the tetraploid state. However, given the small number of AD-4C samples, this observation should be considered hypothesis-generating.

The biological characteristics of these signatures provide a plausible context for the observed shift in mutational profiles. SBS123 is classified by Degasperi et al. (2022) as a transcriptional-strand-biased (TSB) signature, whereas SBS116 is associated with replication-strand bias (RSB); SBS102 is also classified as TSB. These strand biases may reflect differences in the generation, processing, or repair of DNA lesions, although the present data do not establish the underlying molecular mechanisms. The recurrent contribution of SBS116 in the AD 4C samples is of particular interest, as replication-strand-biased mutagenesis could be relevant to cells with altered genome maintenance or cell-cycle regulation associated with tetraploidy. This interpretation remains exploratory and should not be taken as evidence of a specific DNA replication or DNA repair defect.

By contrast, although SBS5 contributed modestly to the AD 4C profiles (13%–14%), its presence in the NoAD control group (27% in NoAD 65) indicates that it is not specific to the tetraploid state. SBS5 is generally considered a clock-like signature and has been reported across a wide range of normal human tissues, including post-mitotic neurons, where its accumulation appears to occur independently of cell division. However, the precise molecular processes underlying SBS5 remain incompletely understood, and its biological interpretation in post-mitotic neural cells remains uncertain.

DNA damage is an early hallmark of AD (Lin et al., 2020). This is consistent with the finding that somatic DNA mutations are usually found in neurons from patients with sporadic AD (Li et al., 2023; Miller et al., 2022; Park et al., 2019). In our study, no significant increase in sSNVs was detected in diploid neurons from patients with AD, in contrast to the study by Li et al. (2023), which focused on the whole neuronal population of the prefrontal cortex. Therefore, an increased sSNV number seems to be specific to SNT. Diploid neurons from control and AD patients were enriched in C > A, C > T, and T > C sSNVs, in agreement with the findings of Miller et al. (2022) and Li et al. (2023), who found two mutational oxidative stress-associated signatures enriched in these mutations in aged individuals as well as in AD patients. Nevertheless, our analysis using the LoFreq variant caller suggested that the single mutations with the lowest frequency observed in diploid (and tetraploid) neurons were largely T > C transitions.

Other studies on AD-associated mutagenesis have focused on somatic mutations affecting known AD risk factor genes (Bushman et al., 2015; Nicolas et al., 2018). Our results confirmed the presence of mutations in AD-risk genes. Nevertheless, mutations associated with PD were also observed to become enriched in the parietal cortex of AD patients. This indicates that the presence of mutations in risk genes does not necessarily result in the generation of the disease to which these genes are associated.

Somatic mutagenesis occurs randomly and can affect any single gene in the genome, accumulating with age. In this regard, Lodato et al. (2018) demonstrated that aging is associated with increased sSNVs in single human neurons from both the cerebral cortex and hippocampus. These authors found that cytosine deamination influences sSNV patterns, resulting in C > T mutations, which account for most variants in the youngest prefrontal cortex samples. This fraction decreases with age, from above 80% in the infant cortex to approximately 50% in the aged cortex. In contrast, T > C variants increased with age, from less than 5% in the infant cortex to approximately 20% in the aged cortex. We found that T > C mutations are enriched in tetraploid neurons from the parietal cortex, suggesting that these aging-associated somatic variants participate in pathological NT.

To further characterize this T > C-enriched spectrum, we performed mutational-signature analysis of the neuronal sSNVs. The observed substitution spectra showed high cosine similarity to established single-base substitution (SBS) reference signatures, with SBS123 and SBS102 accounting for the major contributions in most samples, together with contributions from SBS116 and, in some samples, SBS5. Importantly, these reference signatures were established independently of the PicoPLEX amplification procedure used in the present study. SBS123 and SBS102 were identified by Degasperi et al. (2022) from large-scale WGS datasets comprising 18,640 cancer samples, and SBS102 is represented in the Signal database as SBS102. Thus, the overall substitution spectra observed in our neuronal populations showed similarity to mutational patterns independently identified in WGS datasets that did not use PicoPLEX amplification.

These findings provide population-level evidence that the mutational spectrum observed in tetraploid neurons is structured and is not readily explained by a single nonspecific WGA-associated substitution bias. However, mutational-signature similarity does not establish the authenticity of individual low-frequency sSNVs. Likewise, the 2C/4C comparison cannot exclude stochastic errors introduced during individual WGA reactions. Therefore, the present analyses support the interpretation of a distinct sSNV mutational pattern in tetraploid neurons but do not constitute orthogonal validation of individual candidate variants.

T > C transitions likely represent DNA damage linked to fatty acid oxidation (lipid peroxidation), triggered by intermediates such as 4-hydroxynonenal (4-HNE) or malondialdehyde (MDA) (De Bont and van Larebeke, 2004). This suggests that lipid peroxidation is the major cause of age-associated sSNVs in neurons, consistent with the observation that ROS are a major source of DNA damage in differentiated neurons (Canugovi et al., 2013). In this regard, the accumulation of T > C transitions in the genome of tetraploid neurons is likely associated with the accumulation of sSNVs in BER genes, as the latter are known to encode proteins that repair oxidized bases (Hegde et al., 2012). The observation that BER genes accumulate sSNVs in tetraploid neurons is consistent with the decreased capacity of DNA repair that has been described in the AD brain (Jones et al., 1989; Obulesu et al., 2010; Lillenes et al., 2016), which is manifested through the increase in sSNVs in other types of DNA repair genes. Specific mutagenesis in DNA repair genes may have facilitated the observed accumulation of other somatic DNA mutations in tetraploid neurons. Nevertheless, the enrichment of predicted pathogenic sSNVs in BER, MMR, and NER genes, which likely identifies DNA repair as a genomic pathway preferentially affected by somatic variation in tetraploid neurons, does not necessarily demonstrate impaired DNA repair activity. It is important to point out that transcriptional changes in DNA repair genes would not necessarily reflect the functional consequences of the identified SNVs, since individual variants may affect protein function or other post-transcriptional mechanisms. Moreover, altered DNA repair gene expression in 4C neurons could represent a downstream or compensatory response to genomic damage rather than a direct consequence of the identified mutations. Although our findings do not establish the functional consequences of individual sSNVs for DNA repair activity, they identify DNA repair as a genomic pathway preferentially affected by somatic variation in tetraploid neurons.

As indicated above, our results suggest that tetraploid neurons have been subjected to high levels of oxidative stress, which might have triggered SNT, consistent with the finding that oxidative stress can induce neuronal polyploidy in the Drosophila central nervous system (Nandakumar et al., 2020). We cannot rule out that the accumulation of sSNVs in genes regulating oxidative stress and DNA repair, and the subsequent SNT, could be specific for neurons highly susceptible to oxidative stress (Lee et al., 2015). These neurons would thus acquire protection against oxidation due to genome duplication, as proposed to occur in the brain of Drosophila (Nandakumar et al., 2020) and vertebrates (Yang and Herrup, 2007).

An important limitation of the present study is that all AD cases analyzed were at advanced stages of neuropathology (Braak V/VI), and therefore our data cannot establish whether the accumulation of sSNVs precedes and contributes to neuronal tetraploidization or instead represents a secondary consequence of severe AD-associated cellular stress and genome instability. The increased sSNV burden observed specifically in tetraploid neurons is compatible with a model in which somatic mutations affecting oxidative-stress responses and DNA-repair pathways occur in diploid neurons before or during neuronal cell-cycle re-entry and thereby contribute to the generation of pathological tetraploidy. This interpretation is consistent with our observation that sSNVs are enriched in genes involved in oxidative stress and DNA repair and with previous evidence linking DNA damage, oxidative stress, and aberrant cell-cycle activation in AD neurons. However, an alternative explanation is that neuronal tetraploidization or the severe cellular stress associated with advanced AD pathology occurs first, leading subsequently to increased genomic instability and accumulation of sSNVs. The present cross-sectional analysis cannot distinguish between these possibilities because the neuronal populations were obtained exclusively from late-stage AD brains and no early- or mild-stage AD samples were examined.

Importantly, the absence of an increased sSNV burden in diploid neurons from the AD cases analyzed here does not by itself establish that sSNVs precede tetraploidization. If sSNVs were an initiating event, one might expect some mutations to be detectable in diploid neurons before the acquisition of tetraploidy; however, such mutations could be rare, transient, or restricted to the neuronal subpopulations that subsequently undergo tetraploidization and therefore may not be detectable in the present sample. Conversely, the enrichment of sSNVs in already-tetraploid neurons could reflect the increased genomic stress associated with the tetraploid state. Thus, our findings are consistent with, but do not demonstrate, a model in which somatic mutagenesis contributes to the initiation of SNT. Studies including early- and mild-stage AD, as well as longitudinal or temporally resolved analyses of neuronal ploidy and somatic mutagenesis, will be necessary to determine the temporal relationship between sSNV accumulation and neuronal tetraploidization.

Neurons are postmitotic cells that use cell cycle machinery to regulate neuronal migration, maturation, and synaptic plasticity (Frank and Tsai, 2009). Therefore, it is mandatory for these cells to maintain exquisite control of this machinery to avoid the orchestration of a functional neuronal cell cycle (Frade and Ovejero-Benito, 2015; Walton et al., 2019). Oxidative stress has been postulated to deregulate the control of the cell cycle machinery in neurons, leading to a classical two-hit, oncogenic-like cell cycle (Moh et al., 2011; Zhu et al., 2004, 2007) that may be the basis of SNT and neuronal senescence (Wu et al., 2024). This view is consistent with studies from our laboratory demonstrating that oncogene expression in the absence of functional p53 can induce cell cycle progression in neurons, followed by neuronal hyperploidy (Barrio-Alonso et al., 2018) and neuronal proliferation when the mitotic checkpoints are pharmacologically abolished (Walton et al., 2019).

In this study, we provide evidence consistent with the two-hit oncogenic hypothesis initially postulated by Zhu et al. (2004, 2007) for the generation of pathological NT. We found that sSNVs are highly enriched in genes involved in oxidative stress response and DNA repair in tetraploid neurons. Therefore, random affection of these genes would potentiate the accumulation of oxidative stress in the affected neurons, which would be exacerbated by the lack of DNA repair in these cells. This would enhance the observed accumulation of sSNVs in other genes, including proto-oncogenes and tumor suppressor genes. This view is consistent with the DNA repair deficiency known to occur in neurodegeneration (Jeppesen et al., 2011; Yu et al., 2018). This is also consistent with the increase in sSNVs in neurons from individuals with Cockayne syndrome or xeroderma pigmentosum, two neurodegenerative phenotypes linked to deficient nucleotide excision repair (Lodato et al., 2018). In this context, our results indicate that sSNVs with enhanced MPS are enriched in proto-oncogenes and tumor suppressor genes in tetraploid neurons. This oncogenic-like process would likely participate in the triggering of cell cycle re-entry in AD-affected neurons, as previously observed in differentiated neurons (Barrio-Alonso et al., 2018, 2020; Walton et al., 2019). Therefore, we propose a hypothetical model for the generation of tetraploid neurons in the Alzheimer’s brain based on the mutation of CR genes in diploid neurons due to the inactivation of DNA repair genes, thus facilitating an oncogenic-like cell cycle and SNT (Figure 9).

**Figure 9:**
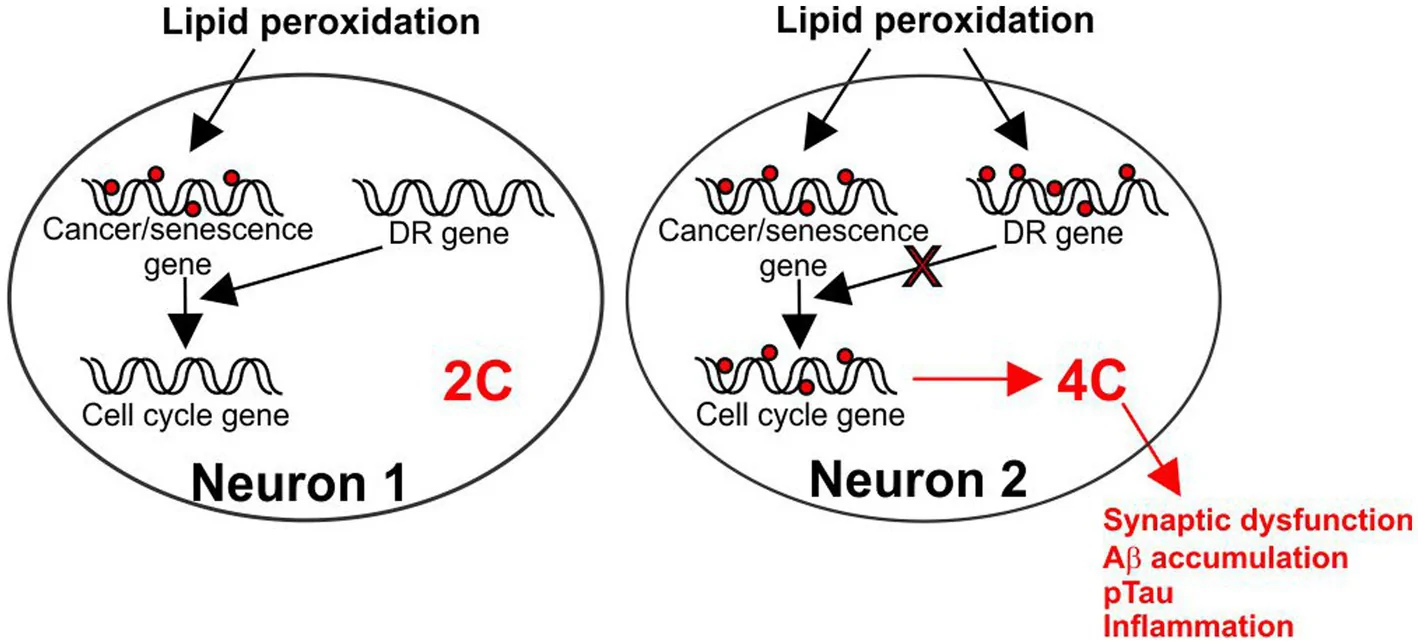
Scheme of the proposed mechanism that triggers the accumulation of sSNVs in diploid neurons, which then become susceptible to become tetraploid. Lipid peroxidation in neurons can trigger random mutations in cancer/senescence genes, which are removed by DNA repair (DR) proteins. Neurons that stochastically accumulate mutations in DR genes become enriched in pathogenic mutations affecting cell cycle/senescence genes. This may facilitate a cancer-like process ending up in tetraploidy (4C) and senescence and their associated neuropathology.

Our results are consistent with the hypothesis that neuronal cell cycle re-entry is triggered by the DNA damage response, ultimately leading to neuronal senescence during both brain aging and Alzheimer's disease (Wong et al., 2023). Supporting this hypothesis, Wu et al. (2024) reported a reduction in DNA repair-associated pathways in neurons that reactivate the cell cycle and subsequently become senescent. Consistent with these findings, we found that senescence-related (SR) genes exhibited higher molecular pathogenicity scores (MPS) than control genes in tetraploid neurons. This hypothetical equivalence between NT and neuronal senescence has profound therapeutic implications, as the multifactorial gene therapy based on E2F4, which is known to prevent NT among other hallmarks of AD (López-Sánchez et al., 2021, 2022; Ramón-Landreau et al., 2022), should be considered to possess senolytic capacity.

Nonetheless, understanding the mechanism leading to neuronal cell cycle re-entry and SNT in AD will facilitate the design of effective therapeutic approaches to prevent and even cure this devastating neuropathological condition.

### 4.1 Limitations of this study

Several limitations of this study should be considered. First, the small sample size (*n* = 3 per group) limits the statistical power and generalizability of our findings. Validation in larger cohorts will be necessary to determine the reproducibility and consistency of the observed mutation patterns across individuals. Second, our analysis was restricted to a small cohort of late-stage AD cases (*n* = 3; Braak V/VI), together with three non-diseased controls, and no early- or mild-stage AD samples were available. Consequently, the present study cannot determine the temporal relationship between sSNV accumulation and neuronal tetraploidization. The enrichment of sSNVs in tetraploid neurons could reflect somatic mutations that contribute to DNA-repair dysfunction, oxidative stress, and subsequent cell-cycle re-entry and tetraploidization; alternatively, it could represent a downstream consequence of the genomic instability and cellular stress associated with established tetraploidy and advanced AD neuropathology. Although our findings are compatible with the former model, they do not establish causality or temporal precedence. Larger cohorts including early and mild stages of AD, ideally combined with analyses of neuronal ploidy and somatic mutations across disease progression, will be required to determine whether sSNVs are an initiating event, a consequence of neuronal tetraploidization, or part of a reciprocal process in which genomic damage and tetraploidization reinforce one another. Third, the identified somatic variants were not validated using orthogonal sequencing approaches or functional assays. Because the detection of low-frequency somatic mutations is technically challenging and whole-genome amplification can introduce artifacts, independent validation of key variants will be important to confirm their authenticity. In particular, PicoPLEX WGA may introduce allelic imbalance, allelic dropout, locus dropout, and polymerase-derived sequence errors, potentially affecting the allele frequencies used to distinguish gSNVs from sSNVs. The additional analyses performed in this study, including the comparison of 2C- and 4C-specific substitution spectra and mutational-signature analysis, provide population-level evidence that the observed T > C enrichment is not readily explained by a uniform WGA-associated mutational bias. Nevertheless, these analyses cannot formally exclude stochastic WGA-derived errors affecting individual low-frequency variant calls. Thus, the possibility that a fraction of the reported low-frequency sSNVs represents technical artefacts cannot be completely excluded.

Orthogonal validation by targeted deep sequencing or digital PCR would provide stronger evidence for individual candidate sSNVs. However, such validation would require independent biological material containing the same rare neuronal variants. Because the relevant variants occur at low frequencies and the neuronal populations represent only a small fraction of bulk tissue, their detection in bulk DNA would be substantially affected by dilution from non-mutant and non-neuronal cells. Single-cell sequencing without WGA would similarly require an alternative experimental workflow and independent biological material. We therefore consider independent orthogonal validation of key candidate variants an important objective for future studies.

Current genomic data only found statistical enrichment of predicted pathogenic variants within BER, MMR and NER genes, but failed to provide any transcriptional functional evidence to prove these SNVs disrupt DNA repair pathway activity in human neurons. Matched RNA-seq of sorted 2C and 4C neurons was not available for the present cohort and would require a dedicated experimental strategy using independent human brain material. Functional studies measuring DNA repair activity and assessing the consequences of specific candidate variants will be required to determine whether the identified sSNVs have a direct functional impact on DNA repair and contribute causally to neuronal tetraploidization.

Currently available brain somatic mosaicism datasets remain limited in terms of the number of individuals, brain regions, and neuronal populations examined, and developmental mosaic variants may be highly individual- and cell-type-specific. Therefore, the absence of a variant from these reference datasets cannot exclude the possibility that it arose through early developmental mosaicism in our samples. Conversely, identification of a variant in a published brain mosaicism dataset would indicate that the same variant has previously been observed in another individual or cellular context, but would not by itself establish that the variant arose during early development in our samples. Thus, while filtering against available brain mosaicism datasets provides an important means of excluding previously reported developmental mosaic variants, it cannot definitively resolve the developmental origin of variants that are not represented in these datasets.

Finally, although our study identifies associations between somatic mutation patterns and neuronal tetraploidy, it does not establish a causal relationship. Functional studies will be required to determine whether these mutations impair DNA repair pathways, promote tetraploidization, or instead arise as a consequence of genome instability associated with the tetraploid state.

## Data Availability

The datasets presented in this study can be found in online repositories. The names of the repository/repositories and accession number(s) can be found below: https://www.ncbi.nlm.nih.gov/bioproject, PRJNA1067670.

## Footnotes

1
https://www.bioinformatics.babraham.ac.uk/projects/fastqc

2
https://www.dreamgenics.com

3
https://fathmm.biocompute.org.uk

5
https://signal.mutationalsignatures.com

6
https://eu.edit.paperpal.com

7
https://cancer.sanger.ac.uk/cosmic

8
https://dcc.icgc.org/search/m

9
https://www.ncbi.nlm.nih.gov/snp
