# Supplementary material for: Specific enrichment of somatic single nucleotide variants in Alzheimer-associated tetraploid neurons
Source: Front Mol Neurosci. 2026 Oct 21;19:1929505. doi: 10.3389/fnmol.2026.1929505 (PMC13731259; doi:10.3389/fnmol.2026.1929505)
Supplement: Supplementary file 1 [file Data_Sheet_1.docx]

Supplementary Material

**Supplementary Figure 1.** Relative frequency distribution of the SNVs within the same AD individuals (AD409 and AD423), classified as either common to both diploid and tetraploid neurons (i.e. germline variants) or specific for either diploid or tetraploid neurons (i.e. somatic mutations). Box: zoom of the graph indicating percentages ranging from 0 to 9.

 Total SNVs	Germline	SNVs	Somatic SNVs

**Supplementary Figure 2.** Absence of most low-frequency SNVs (i.e. sSNVs) in databases containing somatic nucleotide polymorphism. Total SNVs, germline SNVs (i.e. high-frequency SNVs) and somatic SNVs (i.e. low-frequency SNVs) counts with or without identification number (ID) in the catalogue of somatic mutations in cancer (COSMIC), the ICGC data portal, and the reference single nucleotide polymorphism database (dbSNP). *p<0.05, **p<0.01 (comparison NoAD vs AD 4C), #p<0.05, ##p<0.01 (comparison NoAD vs AD 4C) (Student’s t test).

**A**	**B**	**C**

**D**

**Supplementary Figure 3.** Indels from germline and somatic origin. A. Relative frequency distribution of the Indels in NoAD diploid neurons (grey), AD diploid neurons (blue) and AD tetraploid neurons (red). Dotted line represents the threshold that was used to differentiate indels from germline (right) and somatic (left) origin. Box: zoom of the graph indicating percentages ranging from 0 to 9. B. Germline indels observed in NoAD diploid neurons (grey), AD diploid neurons (blue) and AD tetraploid neurons (red). C. Somatic indels observed in NoAD diploid neurons (grey), AD diploid neurons (blue) and AD tetraploid neurons (red). D. Germline (left) and somatic (right) indels counts per chromosomal Mb. Numbers identify the chromosomes. *p<0.05, **p<0.01; *p<005 (comparison NoAD vs AD 4C), #p<0.05 (comparison NoAD vs AD 4C) (Student’s t test).

 Total SNVs	Germline	SNVs	Somatic SNVs

**Supplementary Figure 4.** Total SNVs, germline SNVs (i.e. high-frequency SNVs) and somatic SNVs (i.e. low-frequency SNVs) percentages located in either coding or non-coding regions. *p<0.05, **p<0.01 (comparison NoAD vs AD 4C) (Student’s t test).

**A**

 Total SNVs	Germline	SNVs	Somatic SNVs

**B**

**C**

**Supplementary Figure 5.** Total SNVs, germline SNVs (gSNVs), and somatic SNVs (sSNVs), in the indicated chromosomal regions from all types of neurons. A. Percentages of total SNVs, gSNVs (i.e. high-frequency SNVs) and sSNVs (i.e. low-frequency SNVs) located in either exonic or intronic regions from either diploid (2C) or tetraploid (4C) neurons of control and AD patients. B. Percentages of total SNVs, gSNVs and sSNVs located in either promoters, 5’ untranslated regions (UTR5’) or UTR3’ from either diploid (2C) or tetraploid (4C) neurons of control and AD patients. C. Percentages of total SNVs, gSNVs and sSNVs located in either exon donor, exon acceptor, intron donor or intron acceptor regions from either diploid (2C) or tetraploid (4C) neurons of control and AD patients. *p<0.05, **p<0.01 (comparison NoAD vs AD 4C) (Student’s t test).

**A**

 Total SNVs	Germline	SNVs	Somatic SNVs

**B**

**C**

**Supplementary Figure 6.** Total SNVs, germline SNVs (gSNVs), and somatic SNVs (sSNVs), with the indicated characteristics in all types of neurons. A. Percentages of total SNVs, gSNVs (i.e. high-frequency SNVs) and sSNVs (i.e. low-frequency SNVs) located in splicing regions from either diploid (2C) or tetraploid (4C) neurons of control and AD patients. B. Percentages of total SNVs, gSNVs and sSNVs showing either stop codon gaining or loss in diploid (2C) or tetraploid (4C) neurons of control and AD patients. C. Percentages of total SNVs, gSNVs and sSNVs representing synonymous (syn) or missense mutations in either diploid (2C) or tetraploid (4C) neurons of control and AD patients. *p<0.05, **p<0.01 (comparison NoAD vs AD 4C), #p<0.05, ##p<0.01 (comparison NoAD vs AD 4C) (Student’s t test).

**Supplementary Figure 7.** Percentage of the identified sSNVs in diploid neurons from NoAD individuals and AD patients as well as in tetraploid neurons from AD patients. A. Percentage of the sSNVs common to diploid and tetraploid neurons (grey) or unique to diploid (blue) or tetraploid neurons (red). C/A (C>A, G>T), C/G (C>G, G>C), C/T (C>T, G>A), T/A (T>A, A>T), T/C (T>C, A>G), T/G (T>G, A>C). *p<0.05, **p<0.01 (comparison NoAD vs AD 4C), #p<0.05 (comparison NoAD vs AD 4C) (Student’s t test).

**Supplementary Figure 8.** Mutational signature profiles across samples. Mutational signatures were analyzed from SNVs identified using the highly sensitive LoFreq variant-calling approach with AF<0.05 threshold. A. Aggregated 96 single-base substitution (SBS) mutational profile for the NoAD, AD 2C, and AD 4C experimental groups. B. Left: Individual-sample contributions (%) of the assigned SBS signatures (SBS123, SBS5, SBS116, and SBS102) and cosine similarity scores. Right: Mean mutational signature contributions for each experimental group. Only signatures assigned by the model are shown; the remaining mutations were classified as unassigned and accounted for 0–4% of total mutations. Data are presented as mean ± SEM. Group differences in SBS123 contribution were significant by one-way ANOVA (F(2,5) = 8.10, p = 0.027), with a significant difference between NoAD and AD 4C groups identified by Tukey’s HSD post hoc test (p = 0.025). NoAD, n = 3; AD 2C, n = 3; AD 4C, n = 2. Cosine similarity ranges from 0 to 1, with values closer to 1 indicating greater similarity between the observed and reconstructed mutational profiles. C. Reference mutational signature profiles for the individual SBS signatures (SBS5, SBS102, SBS116, and SBS123) indicated in B.

Counts

|  | NoAD | AD 2C | AD 4C |
| --- | --- | --- | --- |
| ABCA7 | 47.6667 | 67 | 121 |
| ADAM10 | 48 | 45.3333 | 43 |
| APOE | 5 | 6.66667 | 11.5 |
| APP | 80 | 76.3333 | 75 |
| BIN1 | 21.6667 | 33.6667 | 63.5 |
| CD2AP | 23 | 16 | 9 |
| CD33 | 13.3333 | 20.6667 | 32.5 |
| CLU | 11.6667 | 16.6667 | 20.5 |
| CR1 | 65.6667 | 58.3333 | 43 |
| EPHA1 | 25.6667 | 41 | 73 |
| MAPT | 50 | 73.6667 | 97.5 |
| MS4A4A | 61.6667 | 42.3333 | 34.5 |
| MS4A6A | 29.3333 | 27.6667 | 23 |
| PICALM | 37.6667 | 33.6667 | 29 |
| PSEN1 | 27 | 20.3333 | 12 |
| PSEN2 | 8.33333 | 11.3333 | 20 |
| SORL1 | 129.667 | 160.667 | 167.5 |
| TREM2 | 8.66667 | 9.66667 | 17 |
| SMARCA4 | 70.6667 | 79 | 132.5 |
| FERMT2 | 26.3333 | 20.6667 | 10 |
| PTK2B | 81.3333 | 81 | 107.5 |
| CASS4 | 25.3333 | 20.6667 | 29.5 |
| PLD3 | 37.3333 | 44.6667 | 103 |
| TM2D3 | 13 | 12.6667 | 20 |

**Supplementary Figure 9.** sSNVs counts in the indicated AD-risk genes in diploid neurons from NoAD individuals or AD patients as well as in tetraploid neurons from AD patients. *p<0.05, **p<0.01, **p<0.001 (comparison NoAD vs AD 4C) (Student’s t test).

Cancer AD 4C

**Supplementary Figure 10.** Distribution of pathogenicity scores in sSNVs from the indicated gene sets of tetraploid neurons isolated from AD patients. Regression curves and coefficient of regression (R^2^) for the relative frequency distribution of the pathogenicity of the sSNVs is shown.

| SNV Type | Comparison | Mean Difference | Lower 95% CI | Upper 95% CI | Significance (95% CI) |
| --- | --- | --- | --- | --- | --- |
| Total | AD 2C - NoAD | -13456 | -79861.667 | 90932.333 | Not significant |
| Total | AD 4C - NoAD | 87016.667 | 20611 | 194264 | Significant |
| Total | AD 4C - AD 2C | 100472.667 | 89143.333 | 111802 | Significant |
| SNVs | AD 2C - NoAD | -8879 | -72565.333 | 91241 | Not significant |
| SNVs | AD 4C - NoAD | 92925.833 | 29239.5 | 196835.5 | Significant |
| SNVs | AD 4C - AD 2C | 101804.833 | 89435.5 | 112935.333 | Significant |
| INDELs | AD 2C - NoAD | -4241.667 | -7156.333 | -1086.333 | Significant |
| INDELs | AD 4C - NoAD | -5169.833 | -7736.5 | -2014.5 | Significant |
| INDELs | AD 4C - AD 2C | -928.167 | -2884.5 | 199.667 | Not significant |

**Supplementary Table 3.** Summary table derived from non-parametric bootstrapping using the results from Fig. 1A,C,D indicating the SNV types, the mean differences of the specified comparisons, the lower and upper boundaries of the 95% confidence intervals (CI) and the statistical significance (intervals completely excluding zero indicate statistically significant differences).

| SNV Type | Comparison | Mean Difference | Lower 95% CI | Upper 95% CI | Significance (95% CI) |
| --- | --- | --- | --- | --- | --- |
| Germline | AD 2C - NoAD | -59356 | -84494.667 | -28008.983 | Significant |
| Germline | AD 4C - NoAD | -74223.167 | -83186.333 | -64053.5 | Significant |
| Germline | AD 4C - AD 2C | -14867.167 | -50939.5 | 8979.5 | Not significant |
| Somatic | AD 2C - NoAD | 50477 | -25743.667 | 137554.333 | Not significant |
| Somatic | AD 4C - NoAD | 167149 | 108305 | 260889 | Significant |
| Somatic | AD 4C - AD 2C | 116672 | 80456 | 156534 | Significant |

**Supplementary Table 4.** Summary table derived from non-parametric bootstrapping using the results from Fig. 2B,C indicating the SNV types, the mean differences of the specified comparisons, the lower and upper boundaries of the 95% confidence intervals (CI) and the statistical significance (intervals completely excluding zero indicate statistically significant differences).

| SNV Type | Comparison | Mean Difference | Lower 95% CI | Upper 95% CI | Significance (95% CI) |
| --- | --- | --- | --- | --- | --- |
| Chr 1 | AD 2C - NoAD | -19.486 | -28.918 | -7.992 | Significant |
| Chr 1 | AD 4C - NoAD | -24.726 | -28.038 | -20.427 | Significant |
| Chr 1 | AD 4C - AD 2C | -5.24 | -18.282 | 3.203 | Not significant |
| Chr 2 | AD 2C - NoAD | -20.575 | -29.119 | -9.876 | Significant |
| Chr 2 | AD 4C - NoAD | -25.049 | -28.812 | -20.773 | Significant |
| Chr 2 | AD 4C - AD 2C | -4.474 | -15.892 | 3.552 | Not significant |
| Chr 3 | AD 2C - NoAD | -19.946 | -28.302 | -9.036 | Significant |
| Chr 3 | AD 4C - NoAD | -25.207 | -27.406 | -21.783 | Significant |
| Chr 3 | AD 4C - AD 2C | -5.261 | -17.794 | 2.09 | Not significant |
| Chr 4 | AD 2C - NoAD | -18.812 | -26.576 | -8.317 | Significant |
| Chr 4 | AD 4C - NoAD | -23.992 | -26.517 | -21.239 | Significant |
| Chr 4 | AD 4C - AD 2C | -5.18 | -16.933 | 2.299 | Not significant |
| Chr 5 | AD 2C - NoAD | -17.834 | -25.814 | -7.671 | Significant |
| Chr 5 | AD 4C - NoAD | -22.379 | -25.414 | -19.268 | Significant |
| Chr 5 | AD 4C - AD 2C | -4.544 | -15.881 | 3.121 | Not significant |
| Chr 6 | AD 2C - NoAD | -20.356 | -29.647 | -8.818 | Significant |
| Chr 6 | AD 4C - NoAD | -25.905 | -29.329 | -22.203 | Significant |
| Chr 6 | AD 4C - AD 2C | -5.549 | -18.872 | 3.45 | Not significant |
| Chr 7 | AD 2C - NoAD | -19.762 | -28.765 | -7.118 | Significant |
| Chr 7 | AD 4C - NoAD | -25.677 | -28.401 | -22.954 | Significant |
| Chr 7 | AD 4C - AD 2C | -5.916 | -19.598 | 3.131 | Not significant |
| Chr 8 | AD 2C - NoAD | -19.034 | -27.686 | -8.391 | Significant |
| Chr 8 | AD 4C - NoAD | -24.068 | -26.976 | -19.833 | Significant |
| Chr 8 | AD 4C - AD 2C | -5.033 | -17.097 | 2.043 | Not significant |
| Chr 9 | AD 2C - NoAD | -18.037 | -25.602 | -8.138 | Significant |
| Chr 9 | AD 4C - NoAD | -22.926 | -25.482 | -20.079 | Significant |
| Chr 9 | AD 4C - AD 2C | -4.889 | -15.477 | 1.915 | Not significant |
| Chr 10 | AD 2C - NoAD | -21.794 | -31.228 | -10.18 | Significant |
| Chr 10 | AD 4C - NoAD | -27.389 | -30.645 | -22.93 | Significant |
| Chr 10 | AD 4C - AD 2C | -5.595 | -18.7 | 2.661 | Not significant |
| Chr 11 | AD 2C - NoAD | -21.334 | -31.646 | -8.724 | Significant |
| Chr 11 | AD 4C - NoAD | -26.787 | -31.271 | -22.304 | Significant |
| Chr 11 | AD 4C - AD 2C | -5.453 | -19.209 | 4.224 | Not significant |
| Chr 12 | AD 2C - NoAD | -23.644 | -34.649 | -10.276 | Significant |
| Chr 12 | AD 4C - NoAD | -29.989 | -34.036 | -25.023 | Significant |
| Chr 12 | AD 4C - AD 2C | -6.345 | -21.631 | 3.451 | Not significant |
| Chr 13 | AD 2C - NoAD | -16.783 | -24.394 | -7.561 | Significant |
| Chr 13 | AD 4C - NoAD | -21.308 | -24.752 | -17.865 | Significant |
| Chr 13 | AD 4C - AD 2C | -4.525 | -14.564 | 2.431 | Not significant |
| Chr 14 | AD 2C - NoAD | -18.04 | -26.062 | -8.238 | Significant |
| Chr 14 | AD 4C - NoAD | -22.462 | -25.557 | -18.395 | Significant |
| Chr 14 | AD 4C - AD 2C | -4.422 | -14.938 | 2.251 | Not significant |
| Chr 15 | AD 2C - NoAD | -19.956 | -28.902 | -7.869 | Significant |
| Chr 15 | AD 4C - NoAD | -25.102 | -28.503 | -21.701 | Significant |
| Chr 15 | AD 4C - AD 2C | -5.146 | -18.085 | 4.056 | Not significant |
| Chr 16 | AD 2C - NoAD | -21.814 | -32.784 | -8.194 | Significant |
| Chr 16 | AD 4C - NoAD | -28.55 | -32.695 | -23.683 | Significant |
| Chr 16 | AD 4C - AD 2C | -6.736 | -21.768 | 2.845 | Not significant |
| Chr 17 | AD 2C - NoAD | -26.211 | -37.965 | -12.147 | Significant |
| Chr 17 | AD 4C - NoAD | -32.046 | -36.876 | -26.838 | Significant |
| Chr 17 | AD 4C - AD 2C | -5.835 | -20.952 | 4.918 | Not significant |
| Chr 18 | AD 2C - NoAD | -18.249 | -26.154 | -8.39 | Significant |
| Chr 18 | AD 4C - NoAD | -22.353 | -25.793 | -17.936 | Significant |
| Chr 18 | AD 4C - AD 2C | -4.104 | -14.153 | 2.314 | Not significant |
| Chr 19 | AD 2C - NoAD | -33.856 | -49.579 | -15.66 | Significant |
| Chr 19 | AD 4C - NoAD | -42.176 | -49.92 | -34.431 | Significant |
| Chr 19 | AD 4C - AD 2C | -8.319 | -28.233 | 7.637 | Not significant |
| Chr 20 | AD 2C - NoAD | -22.067 | -31.797 | -10.09 | Significant |
| Chr 20 | AD 4C - NoAD | -27.773 | -30.23 | -23.386 | Significant |
| Chr 20 | AD 4C - AD 2C | -5.706 | -19.569 | 2.778 | Not significant |
| Chr 21 | AD 2C - NoAD | -20.067 | -28.987 | -9.042 | Significant |
| Chr 21 | AD 4C - NoAD | -25.259 | -28.474 | -20.67 | Significant |
| Chr 21 | AD 4C - AD 2C | -5.192 | -17.18 | 2.087 | Not significant |
| Chr 22 | AD 2C - NoAD | -21.015 | -34.33 | -7.7 | Significant |
| Chr 22 | AD 4C - NoAD | -26.171 | -35.97 | -17.244 | Significant |
| Chr 22 | AD 4C - AD 2C | -5.155 | -18.044 | 6.395 | Not significant |

**Supplementary Table 5.** Summary table derived from non-parametric bootstrapping using the results from Fig. 2D top (germline) indicating the SNV types, the mean differences of the specified comparisons, the lower and upper boundaries of the 95% confidence intervals (CI) and the statistical significance (intervals completely excluding zero indicate statistically significant differences).

| SNV Type | Comparison | Mean Difference | Lower 95% CI | Upper 95% CI | Significance (95% CI) |
| --- | --- | --- | --- | --- | --- |
| Chr 1 | AD 2C - NoAD | 21.953 | -9.944 | 59.757 | Not significant |
| Chr 1 | AD 4C - NoAD | 72.325 | 49.02 | 110.194 | Significant |
| Chr 1 | AD 4C - AD 2C | 50.372 | 33.234 | 66.926 | Significant |
| Chr 2 | AD 2C - NoAD | 7.604 | -15.068 | 37.076 | Not significant |
| Chr 2 | AD 4C - NoAD | 26.753 | 5.004 | 57.488 | Significant |
| Chr 2 | AD 4C - AD 2C | 19.149 | 11.041 | 25.984 | Significant |
| Chr 3 | AD 2C - NoAD | 12 | -10.257 | 39.484 | Not significant |
| Chr 3 | AD 4C - NoAD | 39.539 | 21.162 | 68.704 | Significant |
| Chr 3 | AD 4C - AD 2C | 27.538 | 17.395 | 36.029 | Significant |
| Chr 4 | AD 2C - NoAD | -0.328 | -15.621 | 19.194 | Significant |
| Chr 4 | AD 4C - NoAD | -2.406 | -17.722 | 17.138 | Significant |
| Chr 4 | AD 4C - AD 2C | -2.078 | -3.484 | 0.394 | Significant |
| Chr 5 | AD 2C - NoAD | 5.76 | -12.758 | 29.384 | Not significant |
| Chr 5 | AD 4C - NoAD | 19.504 | 0.986 | 44.767 | Significant |
| Chr 5 | AD 4C - AD 2C | 13.744 | 8.615 | 18.767 | Significant |
| Chr 6 | AD 2C - NoAD | 11.68 | -9.66 | 39.393 | Not significant |
| Chr 6 | AD 4C - NoAD | 32.845 | 13.53 | 62.467 | Significant |
| Chr 6 | AD 4C - AD 2C | 21.166 | 13.02 | 28.101 | Significant |
| Chr 7 | AD 2C - NoAD | 10.802 | -9.997 | 37.691 | Significant |
| Chr 7 | AD 4C - NoAD | 38.109 | 19.297 | 67.148 | Not significant |
| Chr 7 | AD 4C - AD 2C | 27.307 | 20.307 | 34.854 | Not significant |
| Chr 8 | AD 2C - NoAD | 3.727 | -12.891 | 26.044 | Significant |
| Chr 8 | AD 4C - NoAD | 16.336 | -0.854 | 38.9 | Significant |
| Chr 8 | AD 4C - AD 2C | 12.609 | 8.516 | 15.737 | Not significant |
| Chr 9 | AD 2C - NoAD | 14.911 | -8.117 | 41.409 | Significant |
| Chr 9 | AD 4C - NoAD | 52.262 | 34.87 | 80.477 | Not significant |
| Chr 9 | AD 4C - AD 2C | 37.351 | 26.055 | 49.53 | Not significant |
| Chr 10 | AD 2C - NoAD | 10.997 | -11.335 | 39.464 | Significant |
| Chr 10 | AD 4C - NoAD | 31.127 | 12.388 | 62.313 | Not significant |
| Chr 10 | AD 4C - AD 2C | 20.131 | 11.723 | 28.793 | Not significant |
| Chr 11 | AD 2C - NoAD | 28.917 | -6.608 | 69.993 | Significant |
| Chr 11 | AD 4C - NoAD | 97.805 | 73.259 | 138.881 | Not significant |
| Chr 11 | AD 4C - AD 2C | 68.889 | 49.489 | 90.44 | Not significant |
| Chr 12 | AD 2C - NoAD | 20.476 | -10.899 | 57.463 | Significant |
| Chr 12 | AD 4C - NoAD | 64.014 | 38.532 | 103.842 | Not significant |
| Chr 12 | AD 4C - AD 2C | 43.539 | 30.241 | 57.777 | Not significant |
| Chr 13 | AD 2C - NoAD | 0.723 | -12.356 | 16.705 | Significant |
| Chr 13 | AD 4C - NoAD | 1.058 | -12.12 | 17.139 | Significant |
| Chr 13 | AD 4C - AD 2C | 0.335 | -0.915 | 1.586 | Significant |
| Chr 14 | AD 2C - NoAD | 14.521 | -10.8 | 43.18 | Significant |
| Chr 14 | AD 4C - NoAD | 47.423 | 25.481 | 78.966 | Not significant |
| Chr 14 | AD 4C - AD 2C | 32.902 | 22.071 | 44.549 | Not significant |
| Chr 15 | AD 2C - NoAD | 16.995 | -10.276 | 48.449 | Significant |
| Chr 15 | AD 4C - NoAD | 52.595 | 29.292 | 87.69 | Not significant |
| Chr 15 | AD 4C - AD 2C | 35.6 | 24.154 | 47.568 | Not significant |
| Chr 16 | AD 2C - NoAD | 35.518 | -1.638 | 75.736 | Significant |
| Chr 16 | AD 4C - NoAD | 123.718 | 103.328 | 163.682 | Not significant |
| Chr 16 | AD 4C - AD 2C | 88.2 | 65.176 | 116.239 | Not significant |
| Chr 17 | AD 2C - NoAD | 53.707 | -8.556 | 121.807 | Significant |
| Chr 17 | AD 4C - NoAD | 194.745 | 157.897 | 261.194 | Not significant |
| Chr 17 | AD 4C - AD 2C | 141.038 | 102.324 | 184.164 | Not significant |
| Chr 18 | AD 2C - NoAD | 2.028 | -13.591 | 20.48 | Significant |
| Chr 18 | AD 4C - NoAD | 6.644 | -9.207 | 25.681 | Significant |
| Chr 18 | AD 4C - AD 2C | 4.616 | 1.754 | 6.661 | Not significant |
| Chr 19 | AD 2C - NoAD | 101.558 | 19.533 | 185.693 | Not significant |
| Chr 19 | AD 4C - NoAD | 370.573 | 316.047 | 454.708 | Not significant |
| Chr 19 | AD 4C - AD 2C | 269.015 | 219.851 | 336.267 | Not significant |
| Chr 20 | AD 2C - NoAD | 29.837 | -9.523 | 75.569 | Significant |
| Chr 20 | AD 4C - NoAD | 98.091 | 72.615 | 142.07 | Not significant |
| Chr 20 | AD 4C - AD 2C | 68.255 | 45.624 | 92.536 | Not significant |
| Chr 21 | AD 2C - NoAD | 10.519 | -5.159 | 32.227 | Significant |
| Chr 21 | AD 4C - NoAD | 31.135 | 15.457 | 53.308 | Not significant |
| Chr 21 | AD 4C - AD 2C | 20.617 | 15.75 | 25.483 | Not significant |
| Chr 22 | AD 2C - NoAD | 40.05 | 0.092 | 81.746 | Not significant |
| Chr 22 | AD 4C - NoAD | 146.202 | 123.14 | 187.525 | Not significant |
| Chr 22 | AD 4C - AD 2C | 106.152 | 80.992 | 136.108 | Not significant |

**Supplementary Table 6.** Summary table derived from non-parametric bootstrapping using the results from Fig. 2D bottom (somatic) indicating the SNV types, the mean differences of the specified comparisons, the lower and upper boundaries of the 95% confidence intervals (CI) and the statistical significance (intervals completely excluding zero indicate statistically significant differences).

| SNV Type | Comparison | Mean Difference | Lower 95% CI | Upper 95% CI | Significance (95% CI) |
| --- | --- | --- | --- | --- | --- |
| C>A | AD 2C - NoAD | 3.121 | 1.943 | 4.442 | Significant |
| C>A | AD 4C - NoAD | 0.9 | -0.39 | 2.221 | Not significant |
| C>A | AD 4C - AD 2C | -2.221 | -3.044 | -1.398 | Significant |
| C>G | AD 2C - NoAD | -1.338 | -2.757 | -0.175 | Significant |
| C>G | AD 4C - NoAD | -1.829 | -2.203 | -1.399 | Significant |
| C>G | AD 4C - AD 2C | -0.49 | -1.653 | 1.031 | Not significant |
| C>T | AD 2C - NoAD | -2.512 | -5.257 | 0.308 | Not significant |
| C>T | AD 4C - NoAD | -7.37 | -9.229 | -5.864 | Significant |
| C>T | AD 4C - AD 2C | -4.858 | -7.689 | -2.83 | Significant |
| T>A | AD 2C - NoAD | -1.006 | -2.237 | 0.225 | Not significant |
| T>A | AD 4C - NoAD | -2.464 | -3.23 | -1.418 | Significant |
| T>A | AD 4C - AD 2C | -1.458 | -2.488 | -0.6 | Significant |
| T>C | AD 2C - NoAD | 1.535 | -3.548 | 6.618 | Not significant |
| T>C | AD 4C - NoAD | 11.767 | 8.85 | 14.684 | Significant |
| T>C | AD 4C - AD 2C | 10.232 | 5.273 | 15.276 | Significant |
| T>G | AD 2C - NoAD | 0.2 | -0.65 | 0.914 | Not significant |
| T>G | AD 4C - NoAD | -1.005 | -1.855 | -0.294 | Significant |
| T>G | AD 4C - AD 2C | -1.205 | -1.583 | -0.965 | Significant |

**Supplementary Table 7.** Summary table derived from non-parametric bootstrapping using the results from Fig. 3 (Total) indicating the SNV types, the mean differences of the specified comparisons, the lower and upper boundaries of the 95% confidence intervals (CI) and the statistical significance (intervals completely excluding zero indicate statistically significant differences).

| SNV Type | Comparison | Mean Difference | Lower 95% CI | Upper 95% CI | Significance (95% CI) |
| --- | --- | --- | --- | --- | --- |
| C>A | AD 2C - NoAD | 0.111 | -0.011 | 0.207 | Not significant |
| C>A | AD 4C - NoAD | -0.206 | -0.386 | -0.027 | Not significant |
| C>A | AD 4C - AD 2C | -0.317 | -0.553 | -0.081 | Significant |
| C>G | AD 2C - NoAD | 0.452 | 0.185 | 0.661 | Significant |
| C>G | AD 4C - NoAD | 0.457 | 0.345 | 0.57 | Significant |
| C>G | AD 4C - AD 2C | 0.005 | -0.202 | 0.295 | Not significant |
| C>T | AD 2C - NoAD | 0.617 | 0.252 | 0.938 | Significant |
| C>T | AD 4C - NoAD | -0.704 | -1.01 | -0.383 | Significant |
| C>T | AD 4C - AD 2C | -1.321 | -1.464 | -1.177 | Significant |
| T>A | AD 2C - NoAD | -1 | -1.608 | -0.356 | Significant |
| T>A | AD 4C - NoAD | -1.338 | -1.768 | -0.908 | Significant |
| T>A | AD 4C - AD 2C | -0.338 | -0.982 | 0.198 | Not significant |
| T>C | AD 2C - NoAD | -0.152 | -0.546 | 0.242 | Not significant |
| T>C | AD 4C - NoAD | 2.177 | 1.814 | 2.539 | Significant |
| T>C | AD 4C - AD 2C | 2.328 | 1.865 | 2.792 | Significant |
| T>G | AD 2C - NoAD | -0.028 | -0.288 | 0.251 | Not significant |
| T>G | AD 4C - NoAD | -0.386 | -0.599 | -0.168 | Significant |
| T>G | AD 4C - AD 2C | -0.358 | -0.637 | -0.106 | Significant |

**Supplementary Table 8.** Summary table derived from non-parametric bootstrapping using the results from Fig. 3 (Germline) indicating the SNV types, the mean differences of the specified comparisons, the lower and upper boundaries of the 95% confidence intervals (CI) and the statistical significance (intervals completely excluding zero indicate statistically significant differences).

| SNV Type | Comparison | Mean Difference | Lower 95% CI | Upper 95% CI | Significance (95% CI) |
| --- | --- | --- | --- | --- | --- |
| C>A | AD 2C - NoAD | 1.596 | -0.643 | 4.087 | Not significant |
| C>A | AD 4C - NoAD | -2.013 | -3.877 | 0.493 | Not significant |
| C>A | AD 4C - AD 2C | -3.609 | -5.182 | -1.914 | Significant |
| C>G | AD 2C - NoAD | -0.418 | -0.974 | 0.139 | Not significant |
| C>G | AD 4C - NoAD | -1.01 | -1.372 | -0.542 | Significant |
| C>G | AD 4C - AD 2C | -0.593 | -1.061 | -0.223 | Significant |
| C>T | AD 2C - NoAD | -1.379 | -3.958 | 1.201 | Not significant |
| C>T | AD 4C - NoAD | -5.786 | -7.696 | -4.288 | Significant |
| C>T | AD 4C - AD 2C | -4.408 | -6.78 | -2.583 | Significant |
| T>A | AD 2C - NoAD | -1.563 | -3.272 | 0.145 | Not significant |
| T>A | AD 4C - NoAD | -3.415 | -4.448 | -1.856 | Significant |
| T>A | AD 4C - AD 2C | -1.851 | -3.326 | -0.611 | Significant |
| T>C | AD 2C - NoAD | 1.664 | -4.718 | 8.046 | Not significant |
| T>C | AD 4C - NoAD | 13.514 | 9.736 | 17.292 | Significant |
| T>C | AD 4C - AD 2C | 11.85 | 6.354 | 17.937 | Significant |
| T>G | AD 2C - NoAD | 0.1 | -1.033 | 1.093 | Not significant |
| T>G | AD 4C - NoAD | -1.29 | -2.422 | -0.354 | Significant |
| T>G | AD 4C - AD 2C | -1.389 | -1.935 | -1.023 | Significant |

**Supplementary Table 9.** Summary table derived from non-parametric bootstrapping using the results from Fig. 3 (Somatic) indicating the SNV types, the mean differences of the specified comparisons, the lower and upper boundaries of the 95% confidence intervals (CI) and the statistical significance (intervals completely excluding zero indicate statistically significant differences).

| SNV Type | Comparison | Mean Difference | Lower 95% CI | Upper 95% CI | Significance (95% CI) |
| --- | --- | --- | --- | --- | --- |
| SNVs | AD 2C - NoAD | -46196.33 | -199853.00 | 107460.33 | Not significant |
| SNVs | AD 4C - NoAD | 525728.33 | 352795.00 | 698661.67 | Significant |
| SNVs | AD 4C - AD 2C | 571924.67 | 416175.33 | 727674.00 | Significant |

**Supplementary Table 10.** Summary table derived from non-parametric bootstrapping using the results from Fig. 4A indicating the SNV types, the mean differences of the specified comparisons, the lower and upper boundaries of the 95% confidence intervals (CI) and the statistical significance (intervals completely excluding zero indicate statistically significant differences).

| SNV Type | Comparison | Mean Difference | Lower 95% CI | Upper 95% CI | Significance (95% CI) |
| --- | --- | --- | --- | --- | --- |
| C>A | AD 2C - NoAD | 0.143 | -1.49 | 1.775 | Not significant |
| C>A | AD 4C - NoAD | -3.553 | -4.722 | -2.52 | Significant |
| C>A | AD 4C - AD 2C | -3.695 | -5.045 | -2.349 | Significant |
| C>G | AD 2C - NoAD | -0.08 | -0.915 | 0.754 | Not significant |
| C>G | AD 4C - NoAD | -1.611 | -2.36 | -0.862 | Significant |
| C>G | AD 4C - AD 2C | -1.531 | -2.331 | -0.73 | Significant |
| C>T | AD 2C - NoAD | 0.39 | -0.613 | 1.553 | Not significant |
| C>T | AD 4C - NoAD | -5.63 | -6.414 | -4.433 | Significant |
| C>T | AD 4C - AD 2C | -6.021 | -6.523 | -5.495 | Significant |
| T>A | AD 2C - NoAD | -0.178 | -1.898 | 1.542 | Not significant |
| T>A | AD 4C - NoAD | -1.959 | -3.566 | -0.352 | Significant |
| T>A | AD 4C - AD 2C | -1.781 | -3.519 | -0.043 | Significant |
| T>C | AD 2C - NoAD | -0.167 | -4.694 | 4.36 | Not significant |
| T>C | AD 4C - NoAD | 13.497 | 10.472 | 16.523 | Significant |
| T>C | AD 4C - AD 2C | 13.664 | 9.577 | 17.268 | Significant |
| T>G | AD 2C - NoAD | -0.107 | -0.272 | 0.069 | Not significant |
| T>G | AD 4C - NoAD | -0.744 | -0.909 | -0.579 | Significant |
| T>G | AD 4C - AD 2C | -0.637 | -0.751 | -0.522 | Significant |

**Supplementary Table 11.** Summary table derived from non-parametric bootstrapping using the results from Fig. 4C indicating the SNV types, the mean differences of the specified comparisons, the lower and upper boundaries of the 95% confidence intervals (CI) and the statistical significance (intervals completely excluding zero indicate statistically significant differences).

| SNV Type | Comparison | Mean Difference | Lower 95% CI | Upper 95% CI | Significance (95% CI) |
| --- | --- | --- | --- | --- | --- |
| Total | AD 2C - NoAD | 71.67 | -72.67 | 269.00 | Not significant |
| Total | AD 4C - NoAD | 347.00 | 218.00 | 566.00 | Significant |
| Total | AD 4C - AD 2C | 275.33 | 222.00 | 307.67 | Significant |
| Germline | AD 2C - NoAD | -68.67 | -111.67 | -26.97 | Significant |
| Germline | AD 4C - NoAD | -86.50 | -120.00 | -53.00 | Significant |
| Germline | AD 4C - AD 2C | -17.83 | -58.33 | 22.67 | Not significant |
| Somatic | AD 2C - NoAD | 140.33 | -22.00 | 334.33 | Not significant |
| Somatic | AD 4C - NoAD | 433.50 | 300.50 | 616.00 | Significant |
| Somatic | AD 4C - AD 2C | 293.17 | 199.50 | 359.50 | Significant |

**Supplementary Table 12.** Summary table derived from non-parametric bootstrapping using the results from Fig. 5A indicating the SNV types, the mean differences of the specified comparisons, the lower and upper boundaries of the 95% confidence intervals (CI) and the statistical significance (intervals completely excluding zero indicate statistically significant differences).

| SNV Type | Comparison | Mean Difference | Lower 95% CI | Upper 95% CI | Significance (95% CI) |
| --- | --- | --- | --- | --- | --- |
| Total | AD 2C - NoAD | -0.33 | -62.67 | 109.00 | Not significant |
| Total | AD 4C - NoAD | 180.33 | 118.00 | 293.00 | Significant |
| Total | AD 4C - AD 2C | 180.67 | 174.67 | 186.67 | Significant |
| Germline | AD 2C - NoAD | -63.67 | -98.33 | -39.00 | Significant |
| Germline | AD 4C - NoAD | -70.00 | -97.33 | -42.67 | Significant |
| Germline | AD 4C - AD 2C | -6.33 | -47.67 | 35.00 | Not significant |
| Somatic | AD 2C - NoAD | 63.33 | -14.00 | 162.67 | Not significant |
| Somatic | AD 4C - NoAD | 250.33 | 172.67 | 337.00 | Significant |
| Somatic | AD 4C - AD 2C | 187.00 | 147.33 | 226.67 | Significant |

**Supplementary Table 13.** Summary table derived from non-parametric bootstrapping using the results from Fig. 5B indicating the SNV types, the mean differences of the specified comparisons, the lower and upper boundaries of the 95% confidence intervals (CI) and the statistical significance (intervals completely excluding zero indicate statistically significant differences).

| SNV Type | Comparison | Mean Difference | Lower 95% CI | Upper 95% CI | Significance (95% CI) |
| --- | --- | --- | --- | --- | --- |
| Total | AD 2C - NoAD | 118.33 | -187.67 | 448.00 | Not significant |
| Total | AD 4C - NoAD | 756.50 | 463.50 | 1098.50 | Significant |
| Total | AD 4C - AD 2C | 638.17 | 511.33 | 814.50 | Significant |
| Germline | AD 2C - NoAD | -172.33 | -229.00 | -105.00 | Significant |
| Germline | AD 4C - NoAD | -189.50 | -217.00 | -162.00 | Significant |
| Germline | AD 4C - AD 2C | -17.17 | -90.50 | 39.00 | Not significant |
| Somatic | AD 2C - NoAD | 291.00 | -65.67 | 654.67 | Not significant |
| Somatic | AD 4C - NoAD | 946.33 | 651.00 | 1310.00 | Significant |
| Somatic | AD 4C - AD 2C | 655.33 | 494.00 | 905.00 | Significant |

**Supplementary Table 14.** Summary table derived from non-parametric bootstrapping using the results from Fig. 6A indicating the SNV types, the mean differences of the specified comparisons, the lower and upper boundaries of the 95% confidence intervals (CI) and the statistical significance (intervals completely excluding zero indicate statistically significant differences).

| SNV Type | Comparison | Mean Difference | Lower 95% CI | Upper 95% CI | Significance (95% CI) |
| --- | --- | --- | --- | --- | --- |
| Total | AD 2C - NoAD | 79.67 | -114.67 | 305.00 | Not significant |
| Total | AD 4C - NoAD | 447.17 | 289.50 | 695.50 | Significant |
| Total | AD 4C - AD 2C | 367.50 | 277.50 | 454.50 | Significant |
| Germline | AD 2C - NoAD | -62.33 | -100.33 | -14.33 | Significant |
| Germline | AD 4C - NoAD | -86.83 | -99.67 | -74.00 | Significant |
| Germline | AD 4C - AD 2C | -24.50 | -72.50 | 13.67 | Not significant |
| Somatic | AD 2C - NoAD | 140.00 | -81.00 | 381.00 | Not significant |
| Somatic | AD 4C - NoAD | 532.00 | 373.00 | 766.00 | Significant |
| Somatic | AD 4C - AD 2C | 392.00 | 264.00 | 527.00 | Significant |

**Supplementary Table 15.** Summary table derived from non-parametric bootstrapping using the results from Fig. 6B indicating the SNV types, the mean differences of the specified comparisons, the lower and upper boundaries of the 95% confidence intervals (CI) and the statistical significance (intervals completely excluding zero indicate statistically significant differences).

| SNV Type | Comparison | Mean Difference | Lower 95% CI | Upper 95% CI | Significance (95% CI) |
| --- | --- | --- | --- | --- | --- |
| Total | AD 2C - NoAD | 101.33 | -316.00 | 667.67 | Not significant |
| Total | AD 4C - NoAD | 941.50 | 568.50 | 1544.50 | Significant |
| Total | AD 4C - AD 2C | 840.17 | 711.50 | 987.50 | Significant |
| Germline | AD 2C - NoAD | -204.67 | -303.33 | -83.33 | Significant |
| Germline | AD 4C - NoAD | -264.33 | -301.67 | -227.00 | Significant |
| Germline | AD 4C - AD 2C | -59.67 | -187.00 | 39.00 | Not significant |
| Somatic | AD 2C - NoAD | 306.00 | -180.67 | 852.67 | Not significant |
| Somatic | AD 4C - NoAD | 1205.83 | 827.50 | 1777.00 | Significant |
| Somatic | AD 4C - AD 2C | 899.83 | 672.50 | 1174.50 | Significant |

**Supplementary Table 16.** Summary table derived from non-parametric bootstrapping using the results from Fig. 6C indicating the SNV types, the mean differences of the specified comparisons, the lower and upper boundaries of the 95% confidence intervals (CI) and the statistical significance (intervals completely excluding zero indicate statistically significant differences).

| SNV Type | Comparison | Mean Difference | Lower 95% CI | Upper 95% CI | Significance (95% CI) |
| --- | --- | --- | --- | --- | --- |
| Total | AD 2C - NoAD | 72.00 | -329.00 | 604.00 | Not significant |
| Total | AD 4C - NoAD | 747.67 | 388.00 | 1324.00 | Significant |
| Total | AD 4C - AD 2C | 675.67 | 542.00 | 809.00 | Significant |
| Germline | AD 2C - NoAD | -207.33 | -309.67 | -80.67 | Significant |
| Germline | AD 4C - NoAD | -260.67 | -308.33 | -213.00 | Significant |
| Germline | AD 4C - AD 2C | -53.33 | -190.00 | 52.33 | Not significant |
| Somatic | AD 2C - NoAD | 279.33 | -191.33 | 817.33 | Not significant |
| Somatic | AD 4C - NoAD | 1008.33 | 647.00 | 1553.00 | Significant |
| Somatic | AD 4C - AD 2C | 729.00 | 493.00 | 999.00 | Significant |

**Supplementary Table 17.** Summary table derived from non-parametric bootstrapping using the results from Fig. 6D indicating the SNV types, the mean differences of the specified comparisons, the lower and upper boundaries of the 95% confidence intervals (CI) and the statistical significance (intervals completely excluding zero indicate statistically significant differences).

| SNV Type | Comparison | Mean Difference | Lower 95% CI | Upper 95% CI | Significance (95% CI) |
| --- | --- | --- | --- | --- | --- |
| Total | AD 2C - NoAD | -36.33 | -154.33 | 136.33 | Not significant |
| Total | AD 4C - NoAD | 1.67 | -118.00 | 176.00 | Not significant |
| Total | AD 4C - AD 2C | 38.00 | 15.67 | 60.33 | Significant |
| Germline | AD 2C - NoAD | -91.33 | -139.33 | -43.33 | Significant |
| Germline | AD 4C - NoAD | -112.33 | -148.00 | -76.00 | Significant |
| Germline | AD 4C - AD 2C | -21.00 | -66.00 | 6.00 | Not significant |
| Somatic | AD 2C - NoAD | 55.00 | -64.00 | 188.33 | Not significant |
| Somatic | AD 4C - NoAD | 114.00 | -5.00 | 252.00 | Not significant |
| Somatic | AD 4C - AD 2C | 59.00 | 14.67 | 121.00 | Significant |

**Supplementary Table 18.** Summary table derived from non-parametric bootstrapping using the results from Fig. 6E indicating the SNV types, the mean differences of the specified comparisons, the lower and upper boundaries of the 95% confidence intervals (CI) and the statistical significance (intervals completely excluding zero indicate statistically significant differences).

| SNV Type | Comparison | Mean Difference | Lower 95% CI | Upper 95% CI | Significance (95% CI) |
| --- | --- | --- | --- | --- | --- |
| Total | AD 2C - NoAD | 3.67 | -10.00 | 17.67 | Not significant |
| Total | AD 4C - NoAD | 20.67 | 1.00 | 40.33 | Significant |
| Total | AD 4C - AD 2C | 17.00 | -7.00 | 41.00 | Not significant |
| Germline | AD 2C - NoAD | -7.33 | -11.67 | -3.00 | Significant |
| Germline | AD 4C - NoAD | -7.67 | -13.00 | -2.33 | Significant |
| Germline | AD 4C - AD 2C | -0.33 | -5.33 | 4.67 | Not significant |
| Somatic | AD 2C - NoAD | 11.00 | -5.67 | 27.67 | Not significant |
| Somatic | AD 4C - NoAD | 28.33 | 9.33 | 47.33 | Significant |
| Somatic | AD 4C - AD 2C | 17.33 | -4.33 | 39.00 | Not significant |

**Supplementary Table 19.** Summary table derived from non-parametric bootstrapping using the results from Fig. 6F indicating the SNV types, the mean differences of the specified comparisons, the lower and upper boundaries of the 95% confidence intervals (CI) and the statistical significance (intervals completely excluding zero indicate statistically significant differences).

| SNV Type | Comparison | Mean Difference | Lower 95% CI | Upper 95% CI | Significance (95% CI) |
| --- | --- | --- | --- | --- | --- |
| Total | AD 2C - NoAD | -111.50 | -840.50 | 1001.50 | Not significant |
| Total | AD 4C - NoAD | 761.00 | 32.00 | 1874.00 | Significant |
| Total | AD 4C - AD 2C | 872.50 | 781.00 | 964.00 | Significant |
| Germline | AD 2C - NoAD | -449.33 | -688.67 | -168.67 | Significant |
| Germline | AD 4C - NoAD | -555.17 | -654.00 | -456.33 | Significant |
| Germline | AD 4C - AD 2C | -105.83 | -404.50 | 133.50 | Not significant |
| Somatic | AD 2C - NoAD | 347.33 | -399.33 | 1323.33 | Not significant |
| Somatic | AD 4C - NoAD | 1316.17 | 602.50 | 2344.50 | Significant |
| Somatic | AD 4C - AD 2C | 968.83 | 750.50 | 1248.50 | Significant |

**Supplementary Table 20.** Summary table derived from non-parametric bootstrapping using the results from Fig. 7A indicating the SNV types, the mean differences of the specified comparisons, the lower and upper boundaries of the 95% confidence intervals (CI) and the statistical significance (intervals completely excluding zero indicate statistically significant differences).

| SNV Type | Comparison | Mean Difference | Lower 95% CI | Upper 95% CI | Significance (95% CI) |
| --- | --- | --- | --- | --- | --- |
| Total | AD 2C - NoAD | 111.00 | -95.00 | 317.00 | Not significant |
| Total | AD 4C - NoAD | 469.50 | 355.50 | 659.00 | Significant |
| Total | AD 4C - AD 2C | 358.50 | 221.50 | 526.50 | Significant |
| Germline | AD 2C - NoAD | -74.00 | -115.08 | -33.00 | Significant |
| Germline | AD 4C - NoAD | -89.50 | -123.50 | -63.67 | Significant |
| Germline | AD 4C - AD 2C | -15.50 | -48.50 | 19.50 | Not significant |
| Somatic | AD 2C - NoAD | 185.00 | -31.33 | 401.33 | Not significant |
| Somatic | AD 4C - NoAD | 559.00 | 450.00 | 720.00 | Significant |
| Somatic | AD 4C - AD 2C | 374.00 | 202.00 | 575.00 | Significant |

**Supplementary Table 21.** Summary table derived from non-parametric bootstrapping using the results from Fig. 7B indicating the SNV types, the mean differences of the specified comparisons, the lower and upper boundaries of the 95% confidence intervals (CI) and the statistical significance (intervals completely excluding zero indicate statistically significant differences).

| SNV Type | Comparison | Mean Difference | Lower 95% CI | Upper 95% CI | Significance (95% CI) |
| --- | --- | --- | --- | --- | --- |
| Total | AD 2C - NoAD | 38.67 | -16.33 | 103.67 | Not significant |
| Total | AD 4C - NoAD | 128.00 | 88.00 | 193.00 | Significant |
| Total | AD 4C - AD 2C | 89.33 | 56.00 | 116.00 | Significant |
| Germline | AD 2C - NoAD | -17.33 | -32.33 | 0.00 | Not significant |
| Germline | AD 4C - NoAD | -24.33 | -34.00 | -16.00 | Significant |
| Germline | AD 4C - AD 2C | -7.00 | -25.00 | 3.00 | Not significant |
| Somatic | AD 2C - NoAD | 56.00 | -10.33 | 122.33 | Not significant |
| Somatic | AD 4C - NoAD | 152.33 | 104.00 | 216.00 | Significant |
| Somatic | AD 4C - AD 2C | 96.33 | 54.00 | 141.00 | Significant |

**Supplementary Table 22.** Summary table derived from non-parametric bootstrapping using the results from Fig. 7C indicating the SNV types, the mean differences of the specified comparisons, the lower and upper boundaries of the 95% confidence intervals (CI) and the statistical significance (intervals completely excluding zero indicate statistically significant differences).

| SNV Type | Comparison | Mean Difference | Lower 95% CI | Upper 95% CI | Significance (95% CI) |
| --- | --- | --- | --- | --- | --- |
| Total | AD 2C - NoAD | -21.00 | -183.00 | 188.05 | Not significant |
| Total | AD 4C - NoAD | 216.00 | 54.00 | 430.00 | Significant |
| Total | AD 4C - AD 2C | 237.00 | 202.00 | 272.00 | Significant |
| Germline | AD 2C - NoAD | -99.33 | -152.00 | -34.14 | Significant |
| Germline | AD 4C - NoAD | -127.17 | -151.00 | -101.50 | Significant |
| Germline | AD 4C - AD 2C | -27.83 | -101.50 | 22.00 | Not significant |
| Somatic | AD 2C - NoAD | 78.33 | -73.00 | 264.67 | Not significant |
| Somatic | AD 4C - NoAD | 343.17 | 195.50 | 531.50 | Significant |
| Somatic | AD 4C - AD 2C | 264.83 | 208.00 | 341.50 | Significant |

**Supplementary Table 23.** Summary table derived from non-parametric bootstrapping using the results from Fig. 7D indicating the SNV types, the mean differences of the specified comparisons, the lower and upper boundaries of the 95% confidence intervals (CI) and the statistical significance (intervals completely excluding zero indicate statistically significant differences).

| SNV Type | Comparison | Mean Difference | Lower 95% CI | Upper 95% CI | Significance (95% CI) |
| --- | --- | --- | --- | --- | --- |
| Total | AD 2C - NoAD | -231.67 | -1006.00 | 782.33 | Not significant |
| Total | AD 4C - NoAD | 66.67 | -720.00 | 1093.00 | Not significant |
| Total | AD 4C - AD 2C | 298.33 | 142.33 | 454.33 | Significant |
| Germline | AD 2C - NoAD | -406.67 | -583.00 | -197.80 | Significant |
| Germline | AD 4C - NoAD | -477.00 | -566.00 | -388.00 | Significant |
| Germline | AD 4C - AD 2C | -70.33 | -297.00 | 115.00 | Not significant |
| Somatic | AD 2C - NoAD | 175.00 | -563.67 | 1083.00 | Not significant |
| Somatic | AD 4C - NoAD | 543.67 | -195.00 | 1504.00 | Not significant |
| Somatic | AD 4C - AD 2C | 368.67 | 177.00 | 555.67 | Significant |

**Supplementary Table 24.** Summary table derived from non-parametric bootstrapping using the results from Fig. 7E indicating the SNV types, the mean differences of the specified comparisons, the lower and upper boundaries of the 95% confidence intervals (CI) and the statistical significance (intervals completely excluding zero indicate statistically significant differences).

| SNV Type | Comparison | Mean Difference | Lower 95% CI | Upper 95% CI | Significance (95% CI) |
| --- | --- | --- | --- | --- | --- |
| Total | AD 2C - NoAD | 131.00 | -1177.00 | 1941.33 | Not significant |
| Total | AD 4C - NoAD | 1512.00 | 204.00 | 3376.00 | Significant |
| Total | AD 4C - AD 2C | 1381.00 | 1093.67 | 1668.33 | Significant |
| Germline | AD 2C - NoAD | -720.67 | -1109.00 | -172.00 | Significant |
| Germline | AD 4C - NoAD | -912.50 | -1028.00 | -797.00 | Significant |
| Germline | AD 4C - AD 2C | -191.83 | -755.50 | 196.50 | Not significant |
| Somatic | AD 2C - NoAD | 851.67 | -552.67 | 2460.00 | Not significant |
| Somatic | AD 4C - NoAD | 2424.50 | 1197.50 | 4225.50 | Significant |
| Somatic | AD 4C - AD 2C | 1572.83 | 975.50 | 2160.50 | Significant |

**Supplementary Table 25.** Summary table derived from non-parametric bootstrapping using the results from Fig. 8A indicating the SNV types, the mean differences of the specified comparisons, the lower and upper boundaries of the 95% confidence intervals (CI) and the statistical significance (intervals completely excluding zero indicate statistically significant differences).

| SNV Type | Comparison | Mean Difference | Lower 95% CI | Upper 95% CI | Significance (95% CI) |
| --- | --- | --- | --- | --- | --- |
| Total | AD 2C - NoAD | 142.67 | -2007.00 | 3319.67 | Not significant |
| Total | AD 4C - NoAD | 2976.50 | 880.50 | 6277.50 | Significant |
| Total | AD 4C - AD 2C | 2833.83 | 2256.50 | 3383.00 | Significant |
| Germline | AD 2C - NoAD | -1190.33 | -1720.00 | -496.03 | Significant |
| Germline | AD 4C - NoAD | -1473.00 | -1634.67 | -1284.00 | Significant |
| Germline | AD 4C - AD 2C | -282.67 | -1060.00 | 236.00 | Not significant |
| Somatic | AD 2C - NoAD | 1333.00 | -1157.33 | 4136.67 | Not significant |
| Somatic | AD 4C - NoAD | 4449.50 | 2438.50 | 7561.50 | Significant |
| Somatic | AD 4C - AD 2C | 3116.50 | 2020.50 | 4368.50 | Significant |

**Supplementary Table 26.** Summary table derived from non-parametric bootstrapping using the results from Fig. 8B indicating the SNV types, the mean differences of the specified comparisons, the lower and upper boundaries of the 95% confidence intervals (CI) and the statistical significance (intervals completely excluding zero indicate statistically significant differences).

| SNV Type | Comparison | Mean Difference | Lower 95% CI | Upper 95% CI | Significance (95% CI) |
| --- | --- | --- | --- | --- | --- |
| Total | AD 2C - NoAD | 45.33 | -298.67 | 552.33 | Not significant |
| Total | AD 4C - NoAD | 460.00 | 95.33 | 967.00 | Significant |
| Total | AD 4C - AD 2C | 414.67 | 285.00 | 544.33 | Significant |
| Germline | AD 2C - NoAD | -195.00 | -268.33 | -94.67 | Significant |
| Germline | AD 4C - NoAD | -237.67 | -278.00 | -197.33 | Significant |
| Germline | AD 4C - AD 2C | -42.67 | -147.00 | 48.33 | Not significant |
| Somatic | AD 2C - NoAD | 240.33 | -157.33 | 733.00 | Not significant |
| Somatic | AD 4C - NoAD | 697.67 | 339.00 | 1206.00 | Significant |
| Somatic | AD 4C - AD 2C | 457.33 | 298.67 | 659.00 | Significant |

**Supplementary Table 27.** Summary table derived from non-parametric bootstrapping using the results from Fig. 8C indicating the SNV types, the mean differences of the specified comparisons, the lower and upper boundaries of the 95% confidence intervals (CI) and the statistical significance (intervals completely excluding zero indicate statistically significant differences).

|  | **SNV Type** | **Comparison** | **Mean Difference** | **Lower 95% CI** | **Upper 95% CI** | **Significance (95% CI)** |
| --- | --- | --- | --- | --- | --- | --- |
| **With COSMIC ID** | **Total** | **AD 2C - NoAD** | **-4787.00** | **-9931.00** | **2163.67** | **Not significant** |
|  | **Total** | **AD 4C - NoAD** | **-7112.67** | **-9790.33** | **-4435.00** | **Significant** |
|  | **Total** | **AD 4C - AD 2C** | **-2325.67** | **-9682.00** | **3210.67** | **Not significant** |
|  | **Germline** | **AD 2C - NoAD** | **-7114.67** | **-12290.00** | **-620.00** | **Significant** |
|  | **Germline** | **AD 4C - NoAD** | **-10293.33** | **-12705.00** | **-7881.67** | **Significant** |
|  | **Germline** | **AD 4C - AD 2C** | **-3178.67** | **-9768.00** | **2837.67** | **Not significant** |
|  | **Somatic** | **AD 2C - NoAD** | **2312.67** | **37.67** | **4620.00** | **Significant** |
|  | **Somatic** | **AD 4C - NoAD** | **3180.67** | **1157.00** | **5231.00** | **Significant** |
|  | **Somatic** | **AD 4C - AD 2C** | **868.00** | **-379.67** | **2339.00** | **Not significant** |
| **Without COSMIC ID** | **Total** | **AD 2C - NoAD** | **-4092.00** | **-68202.33** | **92067.00** | **Not significant** |
|  | **Total** | **AD 4C - NoAD** | **100038.50** | **37900.50** | **202068.50** | **Significant** |
|  | **Total** | **AD 4C - AD 2C** | **104130.50** | **87113.50** | **116130.67** | **Significant** |
|  | **Germline** | **AD 2C - NoAD** | **-52240.67** | **-73680.67** | **-25351.18** | **Significant** |
|  | **Germline** | **AD 4C - NoAD** | **-63929.83** | **-71148.00** | **-53093.50** | **Significant** |
|  | **Germline** | **AD 4C - AD 2C** | **-11689.17** | **-41173.50** | **6791.50** | **Not significant** |
|  | **Somatic** | **AD 2C - NoAD** | **21763.33** | **-43839.00** | **102619.33** | **Not significant** |
|  | **Somatic** | **AD 4C - NoAD** | **163968.33** | **107148.00** | **255162.00** | **Significant** |
|  | **Somatic** | **AD 4C - AD 2C** | **142205.00** | **110687.00** | **159480.00** | **Significant** |

**Supplementary Table 28.** Summary table derived from non-parametric bootstrapping using the results from Supplementary Fig. 2A (COSMIC) indicating the SNV types, the mean differences of the specified comparisons, the lower and upper boundaries of the 95% confidence intervals (CI) and the statistical significance (intervals completely excluding zero indicate statistically significant differences).

|  | SNV Type | Comparison | Mean Difference | Lower 95% CI | Upper 95% CI | Significance (95% CI) |
| --- | --- | --- | --- | --- | --- | --- |
| With ICGC ID | Total | AD 2C - NoAD | -17337.00 | -28285.33 | -3216.92 | Significant |
|  | Total | AD 4C - NoAD | -24460.17 | -29342.00 | -19578.33 | Significant |
|  | Total | AD 4C - AD 2C | -7123.17 | -23259.50 | 4322.33 | Not significant |
|  | Germline | AD 2C - NoAD | -20038.67 | -30257.00 | -5819.67 | Significant |
|  | Germline | AD 4C - NoAD | -27228.83 | -31412.67 | -23045.00 | Significant |
|  | Germline | AD 4C - AD 2C | -7190.17 | -21803.50 | 4498.00 | Not significant |
|  | Somatic | AD 2C - NoAD | 2701.67 | -156.33 | 5609.00 | Not significant |
|  | Somatic | AD 4C - NoAD | 2768.67 | 496.33 | 5086.00 | Significant |
|  | Somatic | AD 4C - AD 2C | 67.00 | -1698.67 | 1832.67 | Not significant |
| Without ICGC ID | Total | AD 2C - NoAD | 8458.00 | -59357.33 | 102262.33 | Not significant |
|  | Total | AD 4C - NoAD | 117386.00 | 55955.00 | 216974.00 | Significant |
|  | Total | AD 4C - AD 2C | 108928.00 | 86427.00 | 128854.00 | Significant |
|  | Germline | AD 2C - NoAD | -39317.33 | -55375.67 | -18975.00 | Significant |
|  | Germline | AD 4C - NoAD | -46994.33 | -52006.33 | -38239.00 | Significant |
|  | Germline | AD 4C - AD 2C | -7677.00 | -29136.00 | 5557.00 | Not significant |
|  | Somatic | AD 2C - NoAD | 47775.33 | -27110.33 | 132689.33 | Not significant |
|  | Somatic | AD 4C - NoAD | 164380.33 | 107676.00 | 255213.00 | Significant |
|  | Somatic | AD 4C - AD 2C | 116605.00 | 80870.00 | 157990.00 | Significant |

**Supplementary Table 29.** Summary table derived from non-parametric bootstrapping using the results from Supplementary Fig. 2A (ICGC) indicating the SNV types, the mean differences of the specified comparisons, the lower and upper boundaries of the 95% confidence intervals (CI) and the statistical significance (intervals completely excluding zero indicate statistically significant differences).

|  | SNV Type | Comparison | Mean Difference | Lower 95% CI | Upper 95% CI | Significance (95% CI) |
| --- | --- | --- | --- | --- | --- | --- |
| With dbSNP ID | Total | AD 2C - NoAD | -41885.33 | -68154.00 | -12121.00 | Significant |
|  | Total | AD 4C - NoAD | -44021.50 | -58760.33 | -22775.50 | Significant |
|  | Total | AD 4C - AD 2C | -2136.17 | -31900.50 | 17232.67 | Not significant |
|  | Germline | AD 2C - NoAD | 436.67 | -34341.67 | 35215.00 | Significant |
|  | Germline | AD 4C - NoAD | -15187.17 | -46644.50 | 7297.67 | Significant |
|  | Germline | AD 4C - AD 2C | -15623.83 | -46871.50 | 6660.67 | Significant |
|  | Somatic | AD 2C - NoAD | 8477.00 | -2043.67 | 21494.00 | Not significant |
|  | Somatic | AD 4C - NoAD | 21528.00 | 11837.00 | 35162.00 | Significant |
|  | Somatic | AD 4C - AD 2C | 13051.00 | 8624.00 | 16514.00 | Significant |
| Without dbSNP ID | Total | AD 2C - NoAD | 33006.33 | -32875.33 | 107552.67 | Not significant |
|  | Total | AD 4C - NoAD | 136947.33 | 84473.00 | 219611.00 | Significant |
|  | Total | AD 4C - AD 2C | 103941.00 | 74988.00 | 137495.00 | Significant |
|  | Germline | AD 2C - NoAD | 1880.67 | -2734.33 | 6324.33 | Not significant |
|  | Germline | AD 4C - NoAD | 320.00 | -4295.00 | 4001.67 | Not significant |
|  | Germline | AD 4C - AD 2C | -1560.67 | -4718.67 | 1597.33 | Not significant |
|  | Somatic | AD 2C - NoAD | 42000.00 | -24047.00 | 116516.67 | Not significant |
|  | Somatic | AD 4C - NoAD | 145621.00 | 96468.00 | 224651.00 | Significant |
|  | Somatic | AD 4C - AD 2C | 103621.00 | 71832.00 | 141790.00 | Significant |

**Supplementary Table 30.** Summary table derived from non-parametric bootstrapping using the results from Supplementary Fig. 2A (dbSNP) indicating the SNV types, the mean differences of the specified comparisons, the lower and upper boundaries of the 95% confidence intervals (CI) and the statistical significance (intervals completely excluding zero indicate statistically significant differences).

| SNV Type | Comparison | Mean Difference | Lower 95% CI | Upper 95% CI | Significance (95% CI) |
| --- | --- | --- | --- | --- | --- |
| Germinal | AD 2C - NoAD | -3581.67 | -4926.00 | -1842.29 | Significant |
| Germinal | AD 4C - NoAD | -4647.50 | -5222.33 | -4072.67 | Significant |
| Germinal | AD 4C - AD 2C | -1065.83 | -3037.50 | 338.67 | Not significant |
| Somatic | AD 2C - NoAD | -660.33 | -2988.67 | 1867.67 | Not significant |
| Somatic | AD 4C - NoAD | -522.67 | -2851.00 | 1931.00 | Not significant |
| Somatic | AD 4C - AD 2C | 137.67 | -320.00 | 595.33 | Not significant |

**Supplementary Table 31.** Summary table derived from non-parametric bootstrapping using the results from Supplementary Fig. 4B (Germline) and 4C (Somatic) indicating the SNV types, the mean differences of the specified comparisons, the lower and upper boundaries of the 95% confidence intervals (CI) and the statistical significance (intervals completely excluding zero indicate statistically significant differences).

| SNV Type | Comparison | Mean Difference | Lower 95% CI | Upper 95% CI | Significance (95% CI) |
| --- | --- | --- | --- | --- | --- |
| Chr 1 | AD 2C - NoAD | -19.486 | -28.918 | -7.992 | Significant |
| Chr 1 | AD 4C - NoAD | -24.726 | -28.038 | -20.427 | Significant |
| Chr 1 | AD 4C - AD 2C | -5.24 | -18.282 | 3.203 | Not Significant |
| Chr 2 | AD 2C - NoAD | -20.575 | -29.119 | -9.876 | Significant |
| Chr 2 | AD 4C - NoAD | -25.049 | -28.812 | -20.773 | Significant |
| Chr 2 | AD 4C - AD 2C | -4.474 | -15.892 | 3.552 | Not Significant |
| Chr 3 | AD 2C - NoAD | -19.946 | -28.302 | -9.036 | Significant |
| Chr 3 | AD 4C - NoAD | -25.207 | -27.406 | -21.783 | Significant |
| Chr 3 | AD 4C - AD 2C | -5.261 | -17.794 | 2.09 | Not Significant |
| Chr 4 | AD 2C - NoAD | -18.812 | -26.576 | -8.317 | Significant |
| Chr 4 | AD 4C - NoAD | -23.992 | -26.517 | -21.239 | Significant |
| Chr 4 | AD 4C - AD 2C | -5.18 | -16.933 | 2.299 | Not Significant |
| Chr 5 | AD 2C - NoAD | -17.834 | -25.814 | -7.671 | Significant |
| Chr 5 | AD 4C - NoAD | -22.379 | -25.414 | -19.268 | Significant |
| Chr 5 | AD 4C - AD 2C | -4.544 | -15.881 | 3.121 | Not Significant |
| Chr 6 | AD 2C - NoAD | -20.356 | -29.647 | -8.818 | Significant |
| Chr 6 | AD 4C - NoAD | -25.905 | -29.329 | -22.203 | Significant |
| Chr 6 | AD 4C - AD 2C | -5.549 | -18.872 | 3.45 | Not Significant |
| Chr 7 | AD 2C - NoAD | -19.762 | -28.765 | -7.118 | Significant |
| Chr 7 | AD 4C - NoAD | -25.677 | -28.401 | -22.954 | Significant |
| Chr 7 | AD 4C - AD 2C | -5.916 | -19.598 | 3.131 | Not Significant |
| Chr 8 | AD 2C - NoAD | -19.034 | -27.686 | -8.391 | Significant |
| Chr 8 | AD 4C - NoAD | -24.068 | -26.976 | -19.833 | Significant |
| Chr 8 | AD 4C - AD 2C | -5.033 | -17.097 | 2.043 | Not Significant |
| Chr 9 | AD 2C - NoAD | -18.037 | -25.602 | -8.138 | Significant |
| Chr 9 | AD 4C - NoAD | -22.926 | -25.482 | -20.079 | Significant |
| Chr 9 | AD 4C - AD 2C | -4.889 | -15.477 | 1.915 | Not Significant |
| Chr 10 | AD 2C - NoAD | -21.794 | -31.228 | -10.18 | Significant |
| Chr 10 | AD 4C - NoAD | -27.389 | -30.645 | -22.93 | Significant |
| Chr 10 | AD 4C - AD 2C | -5.595 | -18.7 | 2.661 | Not Significant |
| Chr 11 | AD 2C - NoAD | -21.334 | -31.646 | -8.724 | Significant |
| Chr 11 | AD 4C - NoAD | -26.787 | -31.271 | -22.304 | Significant |
| Chr 11 | AD 4C - AD 2C | -5.453 | -19.209 | 4.224 | Not Significant |
| Chr 12 | AD 2C - NoAD | -23.644 | -34.649 | -10.276 | Significant |
| Chr 12 | AD 4C - NoAD | -29.989 | -34.036 | -25.023 | Significant |
| Chr 12 | AD 4C - AD 2C | -6.345 | -21.631 | 3.451 | Not Significant |
| Chr 13 | AD 2C - NoAD | -16.783 | -24.394 | -7.561 | Significant |
| Chr 13 | AD 4C - NoAD | -21.308 | -24.752 | -17.865 | Significant |
| Chr 13 | AD 4C - AD 2C | -4.525 | -14.564 | 2.431 | Not Significant |
| Chr 14 | AD 2C - NoAD | -18.04 | -26.062 | -8.238 | Significant |
| Chr 14 | AD 4C - NoAD | -22.462 | -25.557 | -18.395 | Significant |
| Chr 14 | AD 4C - AD 2C | -4.422 | -14.938 | 2.251 | Not Significant |
| Chr 15 | AD 2C - NoAD | -19.956 | -28.902 | -7.869 | Significant |
| Chr 15 | AD 4C - NoAD | -25.102 | -28.503 | -21.701 | Significant |
| Chr 15 | AD 4C - AD 2C | -5.146 | -18.085 | 4.056 | Not Significant |
| Chr 16 | AD 2C - NoAD | -21.814 | -32.784 | -8.194 | Significant |
| Chr 16 | AD 4C - NoAD | -28.55 | -32.695 | -23.683 | Significant |
| Chr 16 | AD 4C - AD 2C | -6.736 | -21.768 | 2.845 | Not Significant |
| Chr 17 | AD 2C - NoAD | -26.211 | -37.965 | -12.147 | Significant |
| Chr 17 | AD 4C - NoAD | -32.046 | -36.876 | -26.838 | Significant |
| Chr 17 | AD 4C - AD 2C | -5.835 | -20.952 | 4.918 | Not Significant |
| Chr 18 | AD 2C - NoAD | -18.249 | -26.154 | -8.39 | Significant |
| Chr 18 | AD 4C - NoAD | -22.353 | -25.793 | -17.936 | Significant |
| Chr 18 | AD 4C - AD 2C | -4.104 | -14.153 | 2.314 | Not Significant |
| Chr 19 | AD 2C - NoAD | -33.856 | -49.579 | -15.66 | Significant |
| Chr 19 | AD 4C - NoAD | -42.176 | -49.92 | -34.431 | Significant |
| Chr 19 | AD 4C - AD 2C | -8.319 | -28.233 | 7.637 | Not Significant |
| Chr 20 | AD 2C - NoAD | -22.067 | -31.797 | -10.09 | Significant |
| Chr 20 | AD 4C - NoAD | -27.773 | -30.23 | -23.386 | Significant |
| Chr 20 | AD 4C - AD 2C | -5.706 | -19.569 | 2.778 | Not Significant |
| Chr 21 | AD 2C - NoAD | -20.067 | -28.987 | -9.042 | Significant |
| Chr 21 | AD 4C - NoAD | -25.259 | -28.474 | -20.67 | Significant |
| Chr 21 | AD 4C - AD 2C | -5.192 | -17.18 | 2.087 | Not Significant |
| Chr 22 | AD 2C - NoAD | -21.015 | -34.33 | -7.7 | Significant |
| Chr 22 | AD 4C - NoAD | -26.171 | -35.97 | -17.244 | Significant |
| Chr 22 | AD 4C - AD 2C | -5.155 | -18.044 | 6.395 | Not Significant |

**Supplementary Table 32.** Summary table derived from non-parametric bootstrapping using the results from Supplementary Fig. 4D (Germline) indicating the SNV types, the mean differences of the specified comparisons, the lower and upper boundaries of the 95% confidence intervals (CI) and the statistical significance (intervals completely excluding zero indicate statistically significant differences).

| SNV Type | Comparison | Mean Difference | Lower 95% CI | Upper 95% CI | Significance (95% CI) |
| --- | --- | --- | --- | --- | --- |
| Chr 1 | AD 2C - NoAD | 21.953 | -9.944 | 59.757 | Not significant |
| Chr 1 | AD 4C - NoAD | 72.325 | 49.02 | 110.194 | Significant |
| Chr 1 | AD 4C - AD 2C | 50.372 | 33.234 | 66.926 | Significant |
| Chr 2 | AD 2C - NoAD | 7.604 | -15.068 | 37.076 | Not significant |
| Chr 2 | AD 4C - NoAD | 26.753 | 5.004 | 57.488 | Significant |
| Chr 2 | AD 4C - AD 2C | 19.149 | 11.041 | 25.984 | Significant |
| Chr 3 | AD 2C - NoAD | 12 | -10.257 | 39.484 | Not significant |
| Chr 3 | AD 4C - NoAD | 39.539 | 21.162 | 68.704 | Significant |
| Chr 3 | AD 4C - AD 2C | 27.538 | 17.395 | 36.029 | Significant |
| Chr 4 | AD 2C - NoAD | -0.328 | -15.621 | 19.194 | Not significant |
| Chr 4 | AD 4C - NoAD | -2.406 | -17.722 | 17.138 | Not significant |
| Chr 4 | AD 4C - AD 2C | -2.078 | -3.484 | 0.394 | Not significant |
| Chr 5 | AD 2C - NoAD | 5.76 | -12.758 | 29.384 | Not significant |
| Chr 5 | AD 4C - NoAD | 19.504 | 0.986 | 44.767 | Significant |
| Chr 5 | AD 4C - AD 2C | 13.744 | 8.615 | 18.767 | Significant |
| Chr 6 | AD 2C - NoAD | 11.68 | -9.66 | 39.393 | Not significant |
| Chr 6 | AD 4C - NoAD | 32.845 | 13.53 | 62.467 | Significant |
| Chr 6 | AD 4C - AD 2C | 21.166 | 13.02 | 28.101 | Significant |
| Chr 7 | AD 2C - NoAD | 10.802 | -9.997 | 37.691 | Not significant |
| Chr 7 | AD 4C - NoAD | 38.109 | 19.297 | 67.148 | Significant |
| Chr 7 | AD 4C - AD 2C | 27.307 | 20.307 | 34.854 | Significant |
| Chr 8 | AD 2C - NoAD | 3.727 | -12.891 | 26.044 | Not significant |
| Chr 8 | AD 4C - NoAD | 16.336 | -0.854 | 38.9 | Not significant |
| Chr 8 | AD 4C - AD 2C | 12.609 | 8.516 | 15.737 | Significant |
| Chr 9 | AD 2C - NoAD | 14.911 | -8.117 | 41.409 | Not significant |
| Chr 9 | AD 4C - NoAD | 52.262 | 34.87 | 80.477 | Significant |
| Chr 9 | AD 4C - AD 2C | 37.351 | 26.055 | 49.53 | Significant |
| Chr 10 | AD 2C - NoAD | 10.997 | -11.335 | 39.464 | Not significant |
| Chr 10 | AD 4C - NoAD | 31.127 | 12.388 | 62.313 | Significant |
| Chr 10 | AD 4C - AD 2C | 20.131 | 11.723 | 28.793 | Significant |
| Chr 11 | AD 2C - NoAD | 28.917 | -6.608 | 69.993 | Not significant |
| Chr 11 | AD 4C - NoAD | 97.805 | 73.259 | 138.881 | Significant |
| Chr 11 | AD 4C - AD 2C | 68.889 | 49.489 | 90.44 | Significant |
| Chr 12 | AD 2C - NoAD | 20.476 | -10.899 | 57.463 | Not significant |
| Chr 12 | AD 4C - NoAD | 64.014 | 38.532 | 103.842 | Significant |
| Chr 12 | AD 4C - AD 2C | 43.539 | 30.241 | 57.777 | Significant |
| Chr 13 | AD 2C - NoAD | 0.723 | -12.356 | 16.705 | Not significant |
| Chr 13 | AD 4C - NoAD | 1.058 | -12.12 | 17.139 | Not significant |
| Chr 13 | AD 4C - AD 2C | 0.335 | -0.915 | 1.586 | Not significant |
| Chr 14 | AD 2C - NoAD | 14.521 | -10.8 | 43.18 | Not significant |
| Chr 14 | AD 4C - NoAD | 47.423 | 25.481 | 78.966 | Significant |
| Chr 14 | AD 4C - AD 2C | 32.902 | 22.071 | 44.549 | Significant |
| Chr 15 | AD 2C - NoAD | 16.995 | -10.276 | 48.449 | Not significant |
| Chr 15 | AD 4C - NoAD | 52.595 | 29.292 | 87.69 | Significant |
| Chr 15 | AD 4C - AD 2C | 35.6 | 24.154 | 47.568 | Significant |
| Chr 16 | AD 2C - NoAD | 35.518 | -1.638 | 75.736 | Not significant |
| Chr 16 | AD 4C - NoAD | 123.718 | 103.328 | 163.682 | Significant |
| Chr 16 | AD 4C - AD 2C | 88.2 | 65.176 | 116.239 | Significant |
| Chr 17 | AD 2C - NoAD | 53.707 | -8.556 | 121.807 | Not significant |
| Chr 17 | AD 4C - NoAD | 194.745 | 157.897 | 261.194 | Significant |
| Chr 17 | AD 4C - AD 2C | 141.038 | 102.324 | 184.164 | Significant |
| Chr 18 | AD 2C - NoAD | 2.028 | -13.591 | 20.48 | Not significant |
| Chr 18 | AD 4C - NoAD | 6.644 | -9.207 | 25.681 | Not significant |
| Chr 18 | AD 4C - AD 2C | 4.616 | 1.754 | 6.661 | Significant |
| Chr 19 | AD 2C - NoAD | 101.558 | 19.533 | 185.693 | Significant |
| Chr 19 | AD 4C - NoAD | 370.573 | 316.047 | 454.708 | Significant |
| Chr 19 | AD 4C - AD 2C | 269.015 | 219.851 | 336.267 | Significant |
| Chr 20 | AD 2C - NoAD | 29.837 | -9.523 | 75.569 | Not significant |
| Chr 20 | AD 4C - NoAD | 98.091 | 72.615 | 142.07 | Significant |
| Chr 20 | AD 4C - AD 2C | 68.255 | 45.624 | 92.536 | Significant |
| Chr 21 | AD 2C - NoAD | 10.519 | -5.159 | 32.227 | Not significant |
| Chr 21 | AD 4C - NoAD | 31.135 | 15.457 | 53.308 | Significant |
| Chr 21 | AD 4C - AD 2C | 20.617 | 15.75 | 25.483 | Significant |
| Chr 22 | AD 2C - NoAD | 40.05 | 0.092 | 81.746 | Significant |
| Chr 22 | AD 4C - NoAD | 146.202 | 123.14 | 187.525 | Significant |
| Chr 22 | AD 4C - AD 2C | 106.152 | 80.992 | 136.108 | Significant |

**Supplementary Table 33.** Summary table derived from non-parametric bootstrapping using the results from Supplementary Fig. 4D (Somatic) indicating the SNV types, the mean differences of the specified comparisons, the lower and upper boundaries of the 95% confidence intervals (CI) and the statistical significance (intervals completely excluding zero indicate statistically significant differences).

|  | **SNV Type** | **Comparison** | **Mean Difference** | **Lower 95% CI** | **Upper 95% CI** | **Significance (95% CI)** |
| --- | --- | --- | --- | --- | --- | --- |
| **Coding** | **Total** | **AD 2C - NoAD** | **3.057** | **-0.834** | **6.760** | **Not significant** |
|  | **Total** | **AD 4C - NoAD** | **6.610** | **4.407** | **8.592** | **Significant** |
|  | **Total** | **AD 4C - AD 2C** | **3.553** | **0.422** | **7.444** | **Significant** |
|  | **Germline** | **AD 2C - NoAD** | **3.415** | **1.472** | **4.844** | **Significant** |
|  | **Germline** | **AD 4C - NoAD** | **4.732** | **4.208** | **5.392** | **Significant** |
|  | **Germline** | **AD 4C - AD 2C** | **1.316** | **0.148** | **3.383** | **Significant** |
|  | **Somatic** | **AD 2C - NoAD** | **-0.452** | **-3.217** | **2.314** | **Not significant** |
|  | **Somatic** | **AD 4C - NoAD** | **2.137** | **0.059** | **3.859** | **Significant** |
|  | **Somatic** | **AD 4C - AD 2C** | **2.588** | **0.276** | **5.104** | **Significant** |
| **Non Coding** | **Total** | **AD 2C - NoAD** | **-3.06** | **-6.76** | **0.83** | **Not significant** |
|  | **Total** | **AD 4C - NoAD** | **-6.61** | **-8.59** | **-4.41** | **Significant** |
|  | **Total** | **AD 4C - AD 2C** | **-3.55** | **-7.44** | **-0.42** | **Significant** |
|  | **Germline** | **AD 2C - NoAD** | **-3.42** | **-4.84** | **-1.47** | **Significant** |
|  | **Germline** | **AD 4C - NoAD** | **-4.73** | **-5.39** | **-4.21** | **Significant** |
|  | **Germline** | **AD 4C - AD 2C** | **-1.32** | **-3.38** | **-0.15** | **Significant** |
|  | **Somatic** | **AD 2C - NoAD** | **0.45** | **-2.31** | **3.22** | **Not significant** |
|  | **Somatic** | **AD 4C - NoAD** | **-2.14** | **-3.86** | **-0.06** | **Significant** |
|  | **Somatic** | **AD 4C - AD 2C** | **-2.59** | **-5.10** | **-0.28** | **Significant** |

**Supplementary Table 34.** Summary table derived from non-parametric bootstrapping using the results from Supplementary Fig. 5 indicating the SNV types, the mean differences of the specified comparisons, the lower and upper boundaries of the 95% confidence intervals (CI) and the statistical significance (intervals completely excluding zero indicate statistically significant differences).

|  | **SNV Type** | **Comparison** | **Mean Difference** | **Lower 95% CI** | **Upper 95% CI** | **Significance (95% CI)** |
| --- | --- | --- | --- | --- | --- | --- |
| **Exon** | **Total** | **AD 2C - NoAD** | **2.547** | **-1.125** | **6.163** | **Not significant** |
|  | **Total** | **AD 4C - NoAD** | **5.788** | **3.527** | **7.826** | **Significant** |
|  | **Total** | **AD 4C - AD 2C** | **3.241** | **0.186** | **6.913** | **Significant** |
|  | **Germinal** | **AD 2C - NoAD** | **4.556** | **1.710** | **6.535** | **Significant** |
|  | **Germinal** | **AD 4C - NoAD** | **6.295** | **5.559** | **7.162** | **Significant** |
|  | **Germinal** | **AD 4C - AD 2C** | **1.739** | **-0.037** | **4.657** | **Not significant** |
|  | **Somatic** | **AD 2C - NoAD** | **-0.298** | **-3.081** | **2.485** | **Not significant** |
|  | **Somatic** | **AD 4C - NoAD** | **2.148** | **0.049** | **3.937** | **Significant** |
|  | **Somatic** | **AD 4C - AD 2C** | **2.446** | **0.094** | **4.933** | **Significant** |
| **Intron** | **Total** | **AD 2C - NoAD** | **-1.81** | **-4.90** | **1.29** | **Not significant** |
|  | **Total** | **AD 4C - NoAD** | **-4.42** | **-6.26** | **-2.38** | **Significant** |
|  | **Total** | **AD 4C - AD 2C** | **-2.62** | **-5.65** | **-0.02** | **Significant** |
|  | **Germline** | **AD 2C - NoAD** | **-3.51** | **-5.12** | **-1.07** | **Significant** |
|  | **Germline** | **AD 4C - NoAD** | **-4.88** | **-5.43** | **-4.37** | **Significant** |
|  | **Germline** | **AD 4C - AD 2C** | **-1.37** | **-3.87** | **0.14** | **Not significant** |
|  | **Somatic** | **AD 2C - NoAD** | **0.27** | **-2.29** | **2.83** | **Not significant** |
|  | **Somatic** | **AD 4C - NoAD** | **-1.82** | **-3.54** | **0.20** | **Not significant** |
|  | **Somatic** | **AD 4C - AD 2C** | **-2.09** | **-4.30** | **0.04** | **Not significant** |

**Supplementary Table 35.** Summary table derived from non-parametric bootstrapping using the results from Supplementary Fig. 6A indicating the SNV types, the mean differences of the specified comparisons, the lower and upper boundaries of the 95% confidence intervals (CI) and the statistical significance (intervals completely excluding zero indicate statistically significant differences).

|  | **SNV Type** | **Comparison** | **Mean Difference** | **Lower 95% CI** | **Upper 95% CI** | **Significance (95% CI)** |
| --- | --- | --- | --- | --- | --- | --- |
| **Promoter** | **Total** | **AD 2C - NoAD** | **0.094** | **0.045** | **0.160** | **Significant** |
|  | **Total** | **AD 4C - NoAD** | **0.173** | **0.098** | **0.247** | **Significant** |
|  | **Total** | **AD 4C - AD 2C** | **0.079** | **0.027** | **0.131** | **Significant** |
|  | **Germline** | **AD 2C - NoAD** | **0.225** | **0.067** | **0.334** | **Significant** |
|  | **Germline** | **AD 4C - NoAD** | **0.271** | **0.209** | **0.332** | **Significant** |
|  | **Germline** | **AD 4C - AD 2C** | **0.046** | **-0.085** | **0.207** | **Not significant** |
|  | **Somatic** | **AD 2C - NoAD** | **0.090** | **0.042** | **0.164** | **Significant** |
|  | **Somatic** | **AD 4C - NoAD** | **0.182** | **0.107** | **0.256** | **Significant** |
|  | **Somatic** | **AD 4C - AD 2C** | **0.092** | **0.045** | **0.139** | **Significant** |
| **UTR 5’** | **Total** | **AD 2C - NoAD** | **0.07** | **0.03** | **0.10** | **Significant** |
|  | **Total** | **AD 4C - NoAD** | **0.12** | **0.09** | **0.14** | **Significant** |
|  | **Total** | **AD 4C - AD 2C** | **0.05** | **0.01** | **0.09** | **Significant** |
|  | **Germline** | **AD 2C - NoAD** | **0.18** | **0.06** | **0.26** | **Significant** |
|  | **Germline** | **AD 4C - NoAD** | **0.24** | **0.23** | **0.27** | **Significant** |
|  | **Germline** | **AD 4C - AD 2C** | **0.07** | **-0.02** | **0.19** | **Not significant** |
|  | **Somatic** | **AD 2C - NoAD** | **0.04** | **0.02** | **0.07** | **Significant** |
|  | **Somatic** | **AD 4C - NoAD** | **0.09** | **0.06** | **0.12** | **Significant** |
|  | **Somatic** | **AD 4C - AD 2C** | **0.05** | **0.01** | **0.08** | **Significant** |
| **UTR 3’** | **Total** | **AD 2C - NoAD** | **0.21** | **0.10** | **0.33** | **Significant** |
|  | **Total** | **AD 4C - NoAD** | **0.20** | **0.09** | **0.33** | **Significant** |
|  | **Total** | **AD 4C - AD 2C** | **-0.01** | **-0.10** | **0.08** | **Not significant** |
|  | **Germline** | **AD 2C - NoAD** | **0.24** | **0.10** | **0.32** | **Significant** |
|  | **Germline** | **AD 4C - NoAD** | **0.38** | **0.36** | **0.40** | **Significant** |
|  | **Germline** | **AD 4C - AD 2C** | **0.14** | **0.05** | **0.29** | **Significant** |
|  | **Somatic** | **AD 2C - NoAD** | **0.15** | **0.01** | **0.33** | **Significant** |
|  | **Somatic** | **AD 4C - NoAD** | **0.09** | **-0.06** | **0.27** | **Not significant** |
|  | **Somatic** | **AD 4C - AD 2C** | **-0.06** | **-0.16** | **0.05** | **Not significant** |

**Supplementary Table 36.** Summary table derived from non-parametric bootstrapping using the results from Supplementary Fig. 6B indicating the SNV types, the mean differences of the specified comparisons, the lower and upper boundaries of the 95% confidence intervals (CI) and the statistical significance (intervals completely excluding zero indicate statistically significant differences).

|  | **SNV Type** | **Comparison** | **Mean Difference** | **Lower 95% CI** | **Upper 95% CI** | **Significance (95% CI)** |
| --- | --- | --- | --- | --- | --- | --- |
| **Exon Donor** | **Total** | **AD 2C - NoAD** | **-0.009** | **-0.080** | **0.062** | **Not significant** |
|  | **Total** | **AD 4C - NoAD** | **0.066** | **0.010** | **0.123** | **Significant** |
|  | **Total** | **AD 4C - AD 2C** | **0.075** | **0.012** | **0.139** | **Significant** |
|  | **Germline** | **AD 2C - NoAD** | **0.022** | **0.010** | **0.036** | **Significant** |
|  | **Germline** | **AD 4C - NoAD** | **0.020** | **0.007** | **0.034** | **Significant** |
|  | **Germline** | **AD 4C - AD 2C** | **-0.002** | **-0.016** | **0.011** | **Not significant** |
|  | **Somatic** | **AD 2C - NoAD** | **-0.022** | **-0.109** | **0.066** | **Not significant** |
|  | **Somatic** | **AD 4C - NoAD** | **0.065** | **-0.006** | **0.135** | **Not significant** |
|  | **Somatic** | **AD 4C - AD 2C** | **0.087** | **0.014** | **0.160** | **Significant** |
| **Exon Acceptor** | **Total** | **AD 2C - NoAD** | **-0.01** | **-0.03** | **0.02** | **Not significant** |
|  | **Total** | **AD 4C - NoAD** | **0.01** | **-0.01** | **0.03** | **Not significant** |
|  | **Total** | **AD 4C - AD 2C** | **0.02** | **0.00** | **0.04** | **Not significant** |
|  | **Germline** | **AD 2C - NoAD** | **0.02** | **0.01** | **0.03** | **Significant** |
|  | **Germline** | **AD 4C - NoAD** | **0.02** | **0.01** | **0.03** | **Significant** |
|  | **Germline** | **AD 4C - AD 2C** | **0.00** | **-0.01** | **0.02** | **Not significant** |
|  | **Somatic** | **AD 2C - NoAD** | **-0.01** | **-0.04** | **0.02** | **Not significant** |
|  | **Somatic** | **AD 4C - NoAD** | **0.01** | **-0.02** | **0.04** | **Not significant** |
|  | **Somatic** | **AD 4C - AD 2C** | **0.02** | **0.00** | **0.04** | **Not significant** |
| **Intron Donor** | **Total** | **AD 2C - NoAD** | **-0.01** | **-0.06** | **0.03** | **Not significant** |
|  | **Total** | **AD 4C - NoAD** | **0.02** | **-0.02** | **0.06** | **Not significant** |
|  | **Total** | **AD 4C - AD 2C** | **0.03** | **0.00** | **0.07** | **Not significant** |
|  | **Germline** | **AD 2C - NoAD** | **0.00** | **-0.01** | **0.01** | **Not significant** |
|  | **Germline** | **AD 4C - NoAD** | **0.01** | **-0.01** | **0.02** | **Not significant** |
|  | **Germline** | **AD 4C - AD 2C** | **0.01** | **0.00** | **0.02** | **Not significant** |
|  | **Somatic** | **AD 2C - NoAD** | **-0.03** | **-0.08** | **0.02** | **Not significant** |
|  | **Somatic** | **AD 4C - NoAD** | **0.00** | **-0.04** | **0.05** | **Not significant** |
|  | **Somatic** | **AD 4C - AD 2C** | **0.03** | **-0.01** | **0.07** | **Not significant** |
| **Intron Acceptor** | **Total** | **AD 2C - NoAD** | **-0.06** | **-0.13** | **0.02** | **Not significant** |
|  | **Total** | **AD 4C - NoAD** | **-0.16** | **-0.19** | **-0.12** | **Significant** |
|  | **Total** | **AD 4C - AD 2C** | **-0.10** | **-0.18** | **-0.05** | **Significant** |
|  | **Germline** | **AD 2C - NoAD** | **-0.02** | **-0.05** | **0.00** | **Not significant** |
|  | **Germline** | **AD 4C - NoAD** | **0.03** | **-0.01** | **0.07** | **Not significant** |
|  | **Germline** | **AD 4C - AD 2C** | **0.05** | **0.02** | **0.08** | **Significant** |
|  | **Somatic** | **AD 2C - NoAD** | **-0.13** | **-0.25** | **0.00** | **Significant** |
|  | **Somatic** | **AD 4C - NoAD** | **-0.28** | **-0.36** | **-0.21** | **Significant** |
|  | **Somatic** | **AD 4C - AD 2C** | **-0.15** | **-0.28** | **-0.07** | **Significant** |

**Supplementary Table 37.** Summary table derived from non-parametric bootstrapping using the results from Supplementary Fig. 6C indicating the SNV types, the mean differences of the specified comparisons, the lower and upper boundaries of the 95% confidence intervals (CI) and the statistical significance (intervals completely excluding zero indicate statistically significant differences).

| **SNV Type** | **Comparison** | **Mean Difference** | **Lower 95% CI** | **Upper 95% CI** | **Significance (95% CI)** |
| --- | --- | --- | --- | --- | --- |
| **Total** | **AD 2C - NoAD** | **-0.423** | **-0.603** | **-0.222** | **Significant** |
| **Total** | **AD 4C - NoAD** | **-0.585** | **-0.747** | **-0.423** | **Significant** |
| **Total** | **AD 4C - AD 2C** | **-0.162** | **-0.367** | **0.042** | **Not Significant** |
| **Germline** | **AD 2C - NoAD** | **-0.430** | **-0.680** | **-0.174** | **Significant** |
| **Germline** | **AD 4C - NoAD** | **-0.339** | **-0.540** | **-0.188** | **Significant** |
| **Germline** | **AD 4C - AD 2C** | **0.091** | **-0.165** | **0.273** | **Not Significant** |
| **Somatic** | **AD 2C - NoAD** | **-0.438** | **-0.618** | **-0.239** | **Significant** |
| **Somatic** | **AD 4C - NoAD** | **-0.627** | **-0.788** | **-0.467** | **Significant** |
| **Somatic** | **AD 4C - AD 2C** | **-0.189** | **-0.400** | **0.023** | **Not Significant** |

**Supplementary Table 38.** Summary table derived from non-parametric bootstrapping using the results from Supplementary Fig. 7A indicating the SNV types, the mean differences of the specified comparisons, the lower and upper boundaries of the 95% confidence intervals (CI) and the statistical significance (intervals completely excluding zero indicate statistically significant differences).

|  | **SNV Type** | **Comparison** | **Mean Difference** | **Lower 95% CI** | **Upper 95% CI** | **Significance (95% CI)** |
| --- | --- | --- | --- | --- | --- | --- |
| **Stop Gained** | **Total** | **AD 2C - NoAD** | **-0.204** | **-0.376** | **-0.074** | **Significant** |
|  | **Total** | **AD 4C - NoAD** | **-0.546** | **-0.718** | **-0.427** | **Significant** |
|  | **Total** | **AD 4C - AD 2C** | **-0.342** | **-0.383** | **-0.295** | **Not significant** |
|  | **Germline** | **AD 2C - NoAD** | **-0.089** | **-0.228** | **0.067** | **Not significant** |
|  | **Germline** | **AD 4C - NoAD** | **-0.127** | **-0.266** | **0.038** | **Not significant** |
|  | **Germline** | **AD 4C - AD 2C** | **-0.038** | **-0.064** | **-0.020** | **Significant** |
|  | **Somatic** | **AD 2C - NoAD** | **-0.275** | **-0.413** | **-0.143** | **Significant** |
|  | **Somatic** | **AD 4C - NoAD** | **-0.681** | **-0.820** | **-0.600** | **Significant** |
|  | **Somatic** | **AD 4C - AD 2C** | **-0.407** | **-0.499** | **-0.317** | **Significant** |
| **Stop Lost** | **Total** | **AD 2C - NoAD** | **0.00** | **-0.01** | **0.01** | **Not significant** |
|  | **Total** | **AD 4C - NoAD** | **0.01** | **0.00** | **0.01** | **Significant** |
|  | **Total** | **AD 4C - AD 2C** | **0.01** | **0.00** | **0.02** | **Significant** |
|  | **Germline** | **AD 2C - NoAD** | **-0.03** | **-0.04** | **-0.01** | **Significant** |
|  | **Germline** | **AD 4C - NoAD** | **-0.04** | **-0.06** | **-0.03** | **Significant** |
|  | **Germline** | **AD 4C - AD 2C** | **-0.01** | **-0.03** | **0.01** | **Not significant** |
|  | **Somatic** | **AD 2C - NoAD** | **0.00** | **-0.01** | **0.01** | **Not significant** |
|  | **Somatic** | **AD 4C - NoAD** | **0.01** | **0.00** | **0.01** | **Significant** |
|  | **Somatic** | **AD 4C - AD 2C** | **0.01** | **0.00** | **0.02** | **Significant** |

**Supplementary Table 39.** Summary table derived from non-parametric bootstrapping using the results from Supplementary Fig. 7B indicating the SNV types, the mean differences of the specified comparisons, the lower and upper boundaries of the 95% confidence intervals (CI) and the statistical significance (intervals completely excluding zero indicate statistically significant differences).

|  | **SNV Type** | **Comparison** | **Mean Difference** | **Lower 95% CI** | **Upper 95% CI** | **Significance (95% CI)** |
| --- | --- | --- | --- | --- | --- | --- |
| **Syn** | **Total** | **AD 2C - NoAD** | **-0.582** | **-1.261** | **0.049** | **Not significant** |
|  | **Total** | **AD 4C - NoAD** | **-1.846** | **-2.527** | **-1.410** | **Significant** |
|  | **Total** | **AD 4C - AD 2C** | **-1.264** | **-1.751** | **-0.994** | **Significant** |
|  | **Germline** | **AD 2C - NoAD** | **2.439** | **0.820** | **4.016** | **Significant** |
|  | **Germline** | **AD 4C - NoAD** | **1.496** | **-0.470** | **3.461** | **Not significant** |
|  | **Germline** | **AD 4C - AD 2C** | **-0.944** | **-2.457** | **0.570** | **Not significant** |
|  | **Somatic** | **AD 2C - NoAD** | **-0.077** | **-0.362** | **0.208** | **Not significant** |
|  | **Somatic** | **AD 4C - NoAD** | **-0.706** | **-0.963** | **-0.449** | **Significant** |
|  | **Somatic** | **AD 4C - AD 2C** | **-0.629** | **-0.914** | **-0.362** | **Significant** |
| **Missense** | **Total** | **AD 2C - NoAD** | **1.13** | **0.27** | **1.95** | **Significant** |
|  | **Total** | **AD 4C - NoAD** | **3.07** | **2.67** | **3.72** | **Significant** |
|  | **Total** | **AD 4C - AD 2C** | **1.94** | **1.40** | **2.81** | **Significant** |
|  | **Germline** | **AD 2C - NoAD** | **-1.91** | **-3.15** | **-0.66** | **Significant** |
|  | **Germline** | **AD 4C - NoAD** | **-1.08** | **-2.73** | **0.57** | **Not significant** |
|  | **Germline** | **AD 4C - AD 2C** | **0.83** | **-0.52** | **2.18** | **Not significant** |
|  | **Somatic** | **AD 2C - NoAD** | **0.72** | **0.37** | **1.08** | **Significant** |
|  | **Somatic** | **AD 4C - NoAD** | **2.15** | **1.86** | **2.43** | **Significant** |
|  | **Somatic** | **AD 4C - AD 2C** | **1.43** | **1.06** | **1.79** | **Significant** |

**Supplementary Table 40.** Summary table derived from non-parametric bootstrapping using the results from Supplementary Fig. 7C indicating the SNV types, the mean differences of the specified comparisons, the lower and upper boundaries of the 95% confidence intervals (CI) and the statistical significance (intervals completely excluding zero indicate statistically significant differences).

| **SNV Type** | **Comparison** | **Mean Difference** | **Lower 95% CI** | **Upper 95% CI** | **Significance (95% CI)** |
| --- | --- | --- | --- | --- | --- |
| **C>A** | **Unique 2C - Common 2C-4C** | **-0.772** | **-3.545** | **2** | **Not significant** |
| **C>A** | **Unique 4C - Common 2C-4C** | **-10.587** | **-14.09** | **-7.085** | **Significant** |
| **C>A** | **Unique 4C - Unique 2C** | **-9.815** | **-15.498** | **-4.132** | **Significant** |
| **C>G** | **Unique 2C - Common 2C-4C** | **-0.862** | **-1.397** | **-0.327** | **Significant** |
| **C>G** | **Unique 4C - Common 2C-4C** | **-2.063** | **-2.296** | **-1.83** | **Significant** |
| **C>G** | **Unique 4C - Unique 2C** | **-1.201** | **-1.625** | **-0.776** | **Significant** |
| **C>T** | **Unique 2C - Common 2C-4C** | **2.497** | **1.536** | **3.457** | **Significant** |
| **C>T** | **Unique 4C - Common 2C-4C** | **-9.399** | **-11.675** | **-7.123** | **Significant** |
| **C>T** | **Unique 4C - Unique 2C** | **-11.896** | **-13.213** | **-10.578** | **Significant** |
| **T>A** | **Unique 2C - Common 2C-4C** | **2.442** | **1.318** | **3.567** | **Significant** |
| **T>A** | **Unique 4C - Common 2C-4C** | **-1.569** | **-2.452** | **-0.686** | **Significant** |
| **T>A** | **Unique 4C - Unique 2C** | **-4.011** | **-5.264** | **-2.759** | **Significant** |
| **T>C** | **Unique 2C - Common 2C-4C** | **-2.929** | **-8.837** | **2.978** | **Not significant** |
| **T>C** | **Unique 4C - Common 2C-4C** | **27.047** | **23.789** | **30.304** | **Significant** |
| **T>C** | **Unique 4C - Unique 2C** | **29.976** | **24.339** | **35.613** | **Significant** |
| **T>G** | **Unique 2C - Common 2C-4C** | **-0.375** | **-0.973** | **0.222** | **Not significant** |
| **T>G** | **Unique 4C - Common 2C-4C** | **-3.429** | **-3.64** | **-3.218** | **Significant** |
| **T>G** | **Unique 4C - Unique 2C** | **-3.053** | **-3.779** | **-2.327** | **Significant** |

**Supplementary Table 41.** Summary table derived from non-parametric bootstrapping using the results from Supplementary Fig. 8 indicating the SNV types, the mean differences of the specified comparisons, the lower and upper boundaries of the 95% confidence intervals (CI) and the statistical significance (intervals completely excluding zero indicate statistically significant differences).

| **SNV Type** | **Comparison** | **Mean Difference** | **Lower 95% CI** | **Upper 95% CI** | **Significance (95% CI)** |
| --- | --- | --- | --- | --- | --- |
| **ABCA7** | **AD 2C - NoAD** | **19.667** | **8.000** | **31.333** | **Significant** |
|  | **AD 4C - NoAD** | **75.667** | **62.000** | **89.333** | **Significant** |
|  | **AD 4C - AD 2C** | **56.000** | **44.000** | **68.000** | **Significant** |
| **ADAM10** | **AD 2C - NoAD** | **-3.000** | **-19.000** | **11.333** | **Not significant** |
|  | **AD 4C - NoAD** | **-3.167** | **-19.500** | **11.000** | **Not significant** |
|  | **AD 4C - AD 2C** | **-0.167** | **-1.667** | **1.500** | **Not significant** |
| **APOE** | **AD 2C - NoAD** | **1.333** | **-2.000** | **4.000** | **Not significant** |
|  | **AD 4C - NoAD** | **6.500** | **6.000** | **7.000** | **Significant** |
|  | **AD 4C - AD 2C** | **5.167** | **2.500** | **8.500** | **Significant** |
| **APP** | **AD 2C - NoAD** | **4.667** | **-7.000** | **16.333** | **Not significant** |
|  | **AD 4C - NoAD** | **4.000** | **-7.000** | **15.000** | **Not significant** |
|  | **AD 4C - AD 2C** | **-0.667** | **-12.000** | **10.000** | **Not significant** |
| **BIN1** | **AD 2C - NoAD** | **14.000** | **5.667** | **22.667** | **Significant** |
|  | **AD 4C - NoAD** | **41.833** | **32.000** | **51.667** | **Significant** |
|  | **AD 4C - AD 2C** | **27.833** | **18.000** | **37.667** | **Significant** |
| **CD2AP** | **AD 2C - NoAD** | **-3.333** | **-7.000** | **0.333** | **Not significant** |
|  | **AD 4C - NoAD** | **-8.833** | **-12.500** | **-5.500** | **Significant** |
|  | **AD 4C - AD 2C** | **-5.500** | **-7.500** | **-3.500** | **Significant** |
| **CD33** | **AD 2C - NoAD** | **6.667** | **1.000** | **12.000** | **Significant** |
|  | **AD 4C - NoAD** | **18.667** | **13.000** | **24.333** | **Significant** |
|  | **AD 4C - AD 2C** | **12.000** | **8.333** | **15.667** | **Significant** |
| **CLU** | **AD 2C - NoAD** | **5.000** | **4.000** | **6.333** | **Significant** |
|  | **AD 4C - NoAD** | **9.333** | **6.000** | **12.667** | **Significant** |
|  | **AD 4C - AD 2C** | **4.333** | **0.667** | **8.000** | **Significant** |
| **CR1** | **AD 2C - NoAD** | **1.333** | **-15.667** | **14.000** | **Not significant** |
|  | **AD 4C - NoAD** | **-13.000** | **-30.000** | **-3.000** | **Significant** |
|  | **AD 4C - AD 2C** | **-14.333** | **-21.000** | **-9.667** | **Significant** |
| **EPHA1** | **AD 2C - NoAD** | **16.000** | **7.667** | **27.000** | **Significant** |
|  | **AD 4C - NoAD** | **49.667** | **39.333** | **60.000** | **Significant** |
|  | **AD 4C - AD 2C** | **33.667** | **18.000** | **49.333** | **Significant** |
| **MAPT** | **AD 2C - NoAD** | **16.000** | **1.667** | **30.333** | **Significant** |
|  | **AD 4C - NoAD** | **35.500** | **15.667** | **55.333** | **Significant** |
|  | **AD 4C - AD 2C** | **19.500** | **0.000** | **39.000** | **Not significant** |
| **MS4A4A** | **AD 2C - NoAD** | **-15.333** | **-28.667** | **0.000** | **Not significant** |
|  | **AD 4C - NoAD** | **-21.667** | **-34.000** | **-6.000** | **Significant** |
|  | **AD 4C - AD 2C** | **-6.333** | **-14.000** | **-1.333** | **Significant** |
| **MS4A6A** | **AD 2C - NoAD** | **-1.667** | **-10.333** | **5.000** | **Not significant** |
|  | **AD 4C - NoAD** | **-5.833** | **-14.500** | **1.500** | **Not significant** |
|  | **AD 4C - AD 2C** | **-4.167** | **-5.500** | **-3.000** | **Significant** |
| **PICALM** | **AD 2C - NoAD** | **1.000** | **-7.333** | **11.333** | **Not significant** |
|  | **AD 4C - NoAD** | **-0.167** | **-8.500** | **10.500** | **Not significant** |
|  | **AD 4C - AD 2C** | **-1.167** | **-3.667** | **2.500** | **Not significant** |
| **PSEN1** | **AD 2C - NoAD** | **-4.667** | **-9.333** | **0.667** | **Not significant** |
|  | **AD 4C - NoAD** | **-12.000** | **-14.333** | **-9.667** | **Significant** |
|  | **AD 4C - AD 2C** | **-7.333** | **-13.000** | **-3.000** | **Significant** |
| **PSEN2** | **AD 2C - NoAD** | **3.667** | **1.667** | **6.667** | **Significant** |
|  | **AD 4C - NoAD** | **12.667** | **9.333** | **16.000** | **Significant** |
|  | **AD 4C - AD 2C** | **9.000** | **4.333** | **13.667** | **Significant** |
| **SORL1** | **AD 2C - NoAD** | **35.333** | **15.667** | **60.000** | **Significant** |
|  | **AD 4C - NoAD** | **43.167** | **21.000** | **70.500** | **Significant** |
|  | **AD 4C - AD 2C** | **7.833** | **-6.667** | **22.333** | **Not significant** |
| **TREM2** | **AD 2C - NoAD** | **1.000** | **-7.333** | **9.333** | **Not significant** |
|  | **AD 4C - NoAD** | **7.833** | **1.500** | **14.000** | **Significant** |
|  | **AD 4C - AD 2C** | **6.833** | **-0.500** | **14.000** | **Not significant** |
| **SMARCA4** | **AD 2C - NoAD** | **15.667** | **-6.333** | **36.333** | **Not significant** |
|  | **AD 4C - NoAD** | **72.333** | **57.333** | **89.000** | **Significant** |
|  | **AD 4C - AD 2C** | **56.667** | **39.000** | **80.000** | **Significant** |
| **FERMT2** | **AD 2C - NoAD** | **-2.000** | **-11.667** | **9.000** | **Not significant** |
|  | **AD 4C - NoAD** | **-11.000** | **-19.333** | **0.000** | **Not significant** |
|  | **AD 4C - AD 2C** | **-9.000** | **-14.333** | **-3.667** | **Significant** |
| **PTK2B** | **AD 2C - NoAD** | **11.333** | **1.333** | **23.333** | **Significant** |
|  | **AD 4C - NoAD** | **39.000** | **24.333** | **53.667** | **Significant** |
|  | **AD 4C - AD 2C** | **27.667** | **18.333** | **37.000** | **Significant** |
| **CASS4** | **AD 2C - NoAD** | **1.667** | **-3.000** | **6.333** | **Not significant** |
|  | **AD 4C - NoAD** | **10.667** | **2.333** | **19.000** | **Significant** |
|  | **AD 4C - AD 2C** | **9.000** | **0.667** | **17.333** | **Significant** |
| **PLD3** | **AD 2C - NoAD** | **13.000** | **1.333** | **25.000** | **Significant** |
|  | **AD 4C - NoAD** | **72.000** | **57.000** | **87.000** | **Significant** |
|  | **AD 4C - AD 2C** | **59.000** | **42.333** | **75.667** | **Significant** |
| **TM2D3** | **AD 2C - NoAD** | **3.000** | **-1.667** | **7.667** | **Not significant** |
|  | **AD 4C - NoAD** | **10.333** | **6.000** | **15.000** | **Significant** |
|  | **AD 4C - AD 2C** | **7.333** | **4.667** | **10.000** | **Significant** |

**Supplementary Table 42.** Summary table derived from non-parametric bootstrapping using the results from Supplementary Fig. 9 indicating the SNV types, the mean differences of the specified comparisons, the lower and upper boundaries of the 95% confidence intervals (CI) and the statistical significance (intervals completely excluding zero indicate statistically significant differences).
